# Targeting Lactate‐Driven Stromal Autophagy via MCT1 Disrupts the Immunosuppressive Niche and Sensitizes Pancreatic Cancer to PD‐1 Blockade

**DOI:** 10.1002/advs.76008

**Published:** 2026-06-09

**Authors:** Wenfeng Zhuo, Rong Hu, Yuhang Hu, Ping Hu, Shengbo Han, Guozheng Lv, Zhu Zeng, Yong Zhao, Yang Li, Yan Huang, Yingsong Zhao, Hongda Wang, Guangyu Zhao, Eryang Zhao, Gang Zhao

**Affiliations:** ^1^ Department of Emergency Surgery Union Hospital Tongji Medical College Huazhong University of Science and Technology Wuhan China

**Keywords:** autophagy, lactate, monocarboxylate transporter 1, pancreatic cancer, pancreatic stellate cells, PD‐1

## Abstract

Lactate reshapes the tumor microenvironment (TME) through complex communication between cancer and stromal cells. However, it remains undefined whether lactate mediates the interaction between pancreatic cancer (PC) cells and pancreatic stellate cells (PSCs), a significant TME component driving tumorigenesis. This study elucidates the metabolic crosstalk between PC cells and PSCs underlying lactate‐driven tumor progression. Our findings demonstrate that PC cells serve as the primary lactate source in the TME, where lactate induces PSC activation through an autophagy‐dependent mechanism mediated by protein lactylation. This activation cascade subsequently upregulates programmed cell death‐1 (PD‐1) expression in CD8^+^ T cells, promoting immune evasion. Notably, AZD3965, a specific MCT1 inhibitor, sensitizes orthotopic PC to PD‐1 blockade, effectively inhibiting tumor development. Mechanistically, MCT1‐mediated lactate influx activates PSCs by inducing lactylation of lysine residues K356 and K781 on Vps34, a key autophagy regulator. Moreover, activated PSCs secrete CXCL9/CXCL10, which upregulates PD‐1 expression in CD8^+^ T cells via the CXCR3/STAT3 pathway. This study establishes lactate as a crucial TME signaling molecule orchestrating PSC activation and an immunosuppressive microenvironment, providing compelling evidence for combining MCT1 inhibition with immune checkpoint blockade for pancreatic cancer.

## Graphical abstract

Tumor‐derived lactate activates PSCs through MCT1‐mediated Vps34 lactylation and autophagy. These activated PSCs secrete CXCL9/10, upregulating PD‐1 on CD8^+^ T cells via the CXCR3/STAT3 axis to foster immunosuppression. Disrupting this metabolic crosstalk by targeting MCT1 effectively sensitizes pancreatic cancer to PD‐1 blockade, presenting a promising combination therapy.

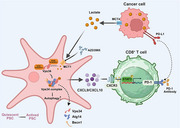

## 1 Introduction

Although cancer immunotherapies have shown significant efficacy in treating various solid tumors, they exhibit notable resistance and poor therapeutic outcomes in PC, a cancer known for its highly immunosuppressive microenvironment. Current immune checkpoint blockade (ICB) therapies, including PD‐1, PD‐L1, and CTLA‐4 inhibitors, have demonstrated limited success in PC [1, 2, 3]. As one of the primary sources of cancer‐associated fibroblasts (CAF), quiescent PSCs play a crucial role in maintaining the normal physiological structure and exocrine function of the pancreas. It is noteworthy that activated PSCs can mediate the formation of an immunosuppressive microenvironment in PC through stromal reprogramming [4], recruiting myeloid‐derived suppressor cells (MDSCs) [5], and induction of M2 macrophage polarization [6]. Therefore, targeting PSCs activation may be a potential strategy to improve the efficacy of immunotherapy in PC.

Research showed that the expression of immune checkpoints on PC cells and stromal cells was extensively regulated by the tumor microenvironmental milieu including cytokine and metabolites [7]. Tumor cells in PC undergo metabolic reprogramming, characterized by aerobic glycolysis, leading to the excessive accumulation of lactate in the TME [8]. Recently, studies have shown that lactate participates in the remodeling of the TME in various solid tumors. Linares et al. reported that lactate reduced p62 level and then activated CAF in prostate cancer through reducing the NAD^+^/NADH ratio [9]. Meanwhile, research showed that lactate promoted M2 polarization of macrophage via the METTL3/OAS3 axis in PC [10]. Moreover, research demonstrated that lactate increased expression of PD‐1 in Treg cells in highly glycolytic TME by promoting NFAT1 translocation to the nucleus [11]. Since these studies intensively indicated that lactate in TME was involved in CAF activation and immune reprogramming in TME, we hypothesized that lactate might be involved in the activation of PSCs and its mediating in immunosuppressed TME of PC.

Recent investigations indicated lactate contributed to the progression and exacerbation of PC by upregulating histone lactylation in H3K18 which consequently increased transcription of target gene including TTK protein kinase (*TTK*) and BUB1 mitotic checkpoint serine/threonine kinase B (*BUB1B*) [12]. Furthermore, Chen et al. demonstrated that lactate prevented NUSAP1 protein degradation through lysine lactylate (Kla) modification, thereby forming a NUSAP1‐LDHA‐glycolysis‐lactate feedforward loop to promote PC metastasis [13]. Additionally, Peng et al. showed that SLC16A1‐mediated lactylation promoted proliferation and migration of PC both in vitro and in vivo. Results also revealed that lactate induced K63 lactylation of Endosulfine Alpha (ENSA‐K63la) and then enhanced tumor‐associated macrophage recruitment to the TME by regulating the STAT3/CCL2 pathway [14]. Given the fact that lactylation is exerting critical roles in the progression of PC, we further investigated whether lactate facilitates PSCs activation via mediating lactylation.

In this study, varying concentrations of lactate were supplemented in PSCs culture medium to evaluate its effects on PSCs activation. Additionally, VPS34 lactylation site mutant plasmids were generated to investigate the mechanisms by which MCT1‐mediated lactylation induces PSCs activation. We then developed an orthotopic PC model in PSCs‐specific MCT1 knockout (PSCs‐MCT1^−/−^) mice to assess the role of MCT1‐mediated lactate influx into PSCs in PC tumor progression. Single‐cell sequencing in MCT1^−/−^ mice with PC was further used to observe the effects of MCT1‐mediated lactate influx in PSCs on immune microenvironment remodeling. Finally, we evaluated the efficacy of a novel therapy combining MCT1 specific inhibitor AZD3965 and PD‐1 monoclonal antibody in PC, aiming to illustrate the importance of targeting PSCs therapy in the context of the dense and immunosuppressive microenvironment of PC, and to provide a promising therapeutic target for PC therapy.

## 2 Results

### 2.1 Lactate is an Important Signaling Molecule Mediating the Crosstalk Between PC Cells and PSCs

**FIGURE 1:**
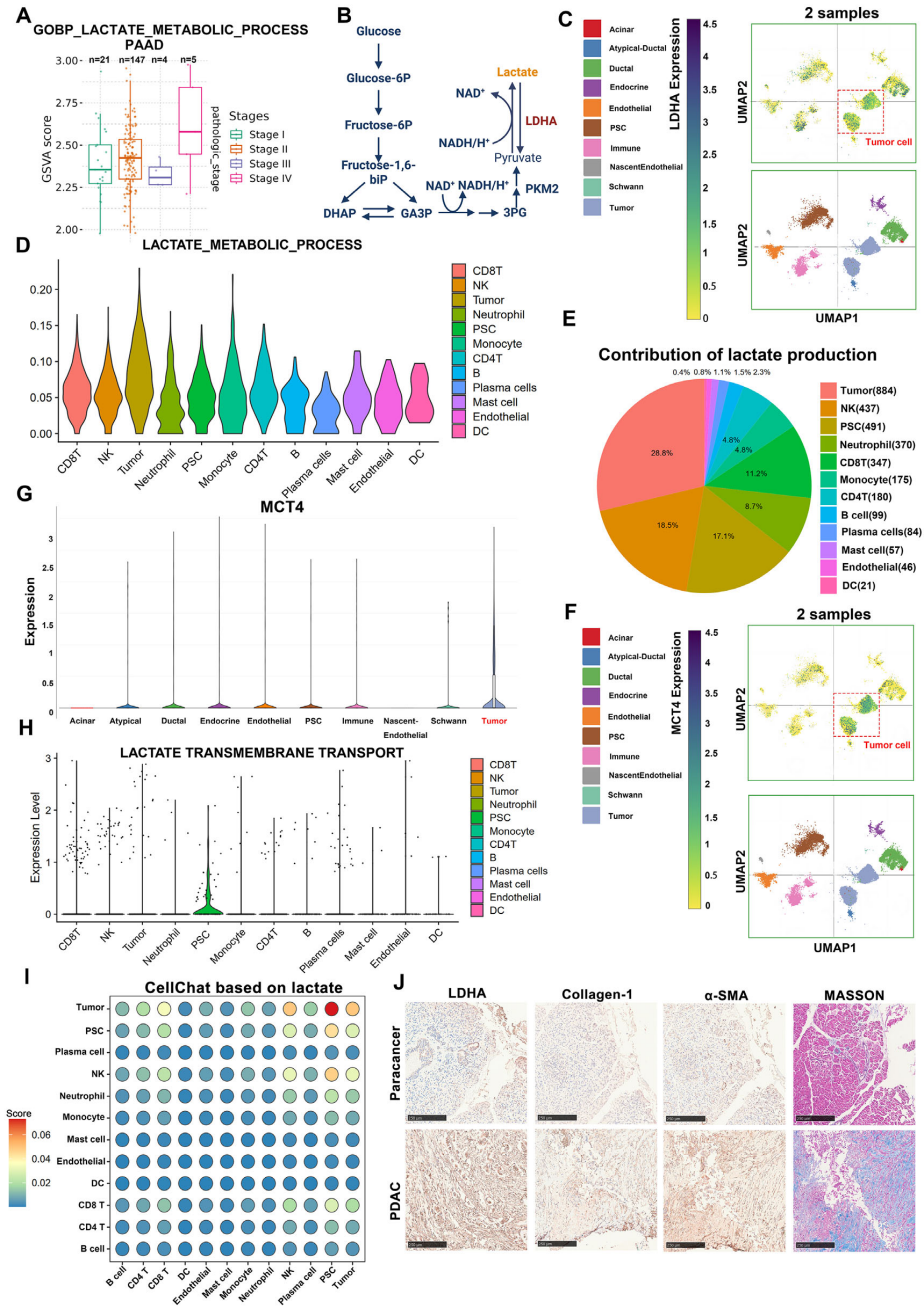
Lactate is an important signaling molecule mediating the crosstalk between PC cells and PSCs. (A) The score of lactate metabolism dataset in different stages of PC based on TCGA data. (B) Lactate production pathway in PC cells. (C) Single‐cell atlas of LDHA expression in human PC patients. (D) The score of lactate metabolism dataset in different cells based on PC patients scRNA‐seq data. (E) Contribution of different cell types to lactate production in the TME based on the weighted scores of lactate metabolism dataset and cell type abundance. (F‐G) Single‐cell atlas and violin plot of MCT4 expression in human PC patients. (H) The score of lactate transmembrane transport dataset in different cells based on PC patients scRNA‐seq data. (I) Lactate metabolic communications between different cell types in the human breast cancer TME. Interaction intensity was calculated based on the score of the lactate metabolism dataset and lactate transmembrane transport dataset. (J) IHC of LDHA, Collagen I, and α‐SMA, as well as Masson's staining of tumor and adjacent normal tissue sections derived from human PDAC patients (*n* = 5). Scale bars: 250 µm.

To investigate the role of lactate metabolism in PC progression, we analyzed the scores of the lactate metabolism pathway and clinical stages of patients with pancreatic adenocarcinoma (PAAD) in the TCGA cohort (Figure 1A). The results showed that the lactate metabolism pathway score increased in a stepwise gradient across stages I, II, and IV, and was negatively correlated with the infiltration of CD4^+^ and CD8^+^ T cells, as well as NK cells (Figure S1A) based on the TCGA database. Additionally, high expression groups of LDHA and PKM2, two key enzymes mediating lactate synthesis, exhibited significantly worse prognoses (Figure S1B), indicating that dysregulated lactate metabolism may play a critical role in PC progression. Under hypoxic conditions, glucose is primarily converted to lactate via the glycolytic pathway (Figure 1B). To map the landscape of lactate production in PC and identify the major contributors to lactate biosynthesis within TME, we analyzed the single‐cell atlas of human PC (PRJCA001063) [15], focusing on the expression and distribution of LDHA and LDHB. The result showed LDHA and LDHB was highest expressed in tumor cells of PC (Figure 1C and Figure S1C). Next, we evaluated the lactate metabolism pathway scores across different cell populations, and used weighted calculations to estimate the contribution percentage of various cell types to lactate production in the TME by integrating the expression levels of key genes involved in lactate biosynthesis and the abundance of each cell type. The results indicated tumor cells had the highest lactate pathway score (Figure 1D), 28.8% of lactate was contributed by tumor cells of PC (Figure 1E). MCT4, a major monocarboxylate transporter mediating intracellular‐to‐extracellular lactate transport, was also found to be predominantly expressed in tumor cells based on its expression and distribution in the single‐cell atlas (Figure 1F,G), further supporting that circulating lactate in the TME is primarily supplied by PC cells. In addition, MCT2 and MCT3 are also highly expressed in the tumor cells, while MCT1 is mainly expressed in the PSCs (Figure S1D).

To elucidate the lactate metabolic interactions between PC cells and other cells in the TME, we performed scoring of lactate transmembrane transport pathways using single cell RNA‐seq data (PRJCA001063) from human PC and analyzed lactate metabolic interactions across different cell types. The results indicated the PSCs were the main transmembrane transporter cells for lactate (Figure 1H), with the strongest lactate metabolic communication between PC cells and PSCs (Figure 1I). TCGA data further revealed that activation markers of PSCs (COL1A1 and ACTA2) were positively correlated with key enzymes of lactate synthesis (LDHA and PKM2) (Figure S1E). Pathological sections from PDAC patients at our hospital also showed that the expression levels of LDHA and fibrosis markers (Collagen I and α‐SMA) in cancer tissues were significantly higher than those in adjacent non‐tumor tissues (Figure 1J and Figure S1F). TCGA data also showed that COL1A1 and ACTA2 exhibited stepwise upregulation across stages I to III in PDAC patients (Figure S1G).

These findings suggest that in the TME of PDAC, lactate is primarily generated by PC cells and likely shuttled in large quantities to PSCs, thereby mediating PSCs activation and tissue fibrosis, which further accelerates PC progression.

### 2.2 Lactate Can Induce Activation of PSCs

**FIGURE 2:**
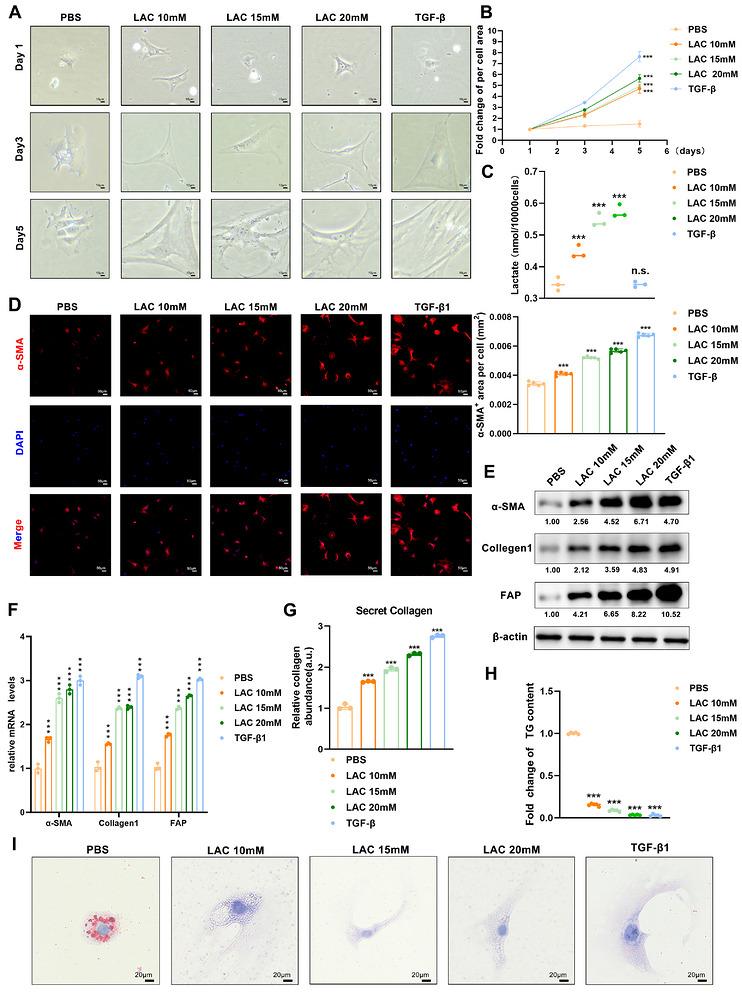
Lactate can induce activation of PSCs. (A) Representative microscopic images of primary mouse stellate cells under PBS, LAC 10 mm, LAC 15 mm, LAC 20 mm, and TGF‐β1 treatment conditions at 1 day, 3 days, and 5 days (*n* = 5), scale bars 10 µm. (B) Fold change of per PSCs area in Figure 2A. (C) Intracellular lactate concentration in PSCs (*n* = 3). (D) Representative α‐SMA fluorescence staining images of primary mouse stellate cells treated with PBS, lactate or TGF‐β1 for 3 days (*n* = 5), with nuclei stained by DAPI. Scale bars: 50 µm. α‐SMA positive area of individual PSCs. (E) Western blot analysis of α‐SMA, Collagen I, and FAP expression in the PSCs (*n* = 3). (F) The mRNA levels of Collagen I, α‐SMA, FAP were measured by quantitative RTPCR assays (*n* = 3). (G) Collagen I in the supernatant of PSCs culture medium was detected using the ELISA kit (*n* = 3). (H) Intracellular TG concentration in PSCs. (I) Lipid droplets in PSC were stained with Oil Red O and observed under a light microscope, with nuclei stained by hematoxylin (*n* = 3). Scale bars: 20 µm. Data are mean and ± SEM. ^***^ *p* < 0.001. Statistical significance was determined by one‐way ANOVA or two‐sided Student's t‐test as appropriate.

To determine whether lactate directly promotes PSCs activation, we isolated primary PSCs and cultured with lactate in various concentration. As anticipated, with increasing concentrations of lactate added to the culture medium, PSCs exhibited significant morphological changes to a myofibroblastic shape with increased cell area (Figure 2A,B). Intracellular lactate measurement showed that as the amount of lactate added extracellularly increased, the intracellular lactate content also increased in a gradient (Figure 2C). Results from IF showed that α‐SMA expression was also increased with lactate treatment (Figure 2D). Meanwhile, Western blot and qPCR analysis further confirmed that both protein and mRNA levels of α‐SMA, Collagen‐1, and FAP in PSCs were increased in a dose‐dependent manner (Figure 2E,F). Additionally, the level of collagen I secretion in PSCs was also increased in lactate supplementation (Figure 2G). One of the distinguishing features of PSCs from other CAF sources is their abundance of lipid droplets in the quiescent state [16]. Determination of TG content and observation under an optical microscope after Oil Red O staining showed that the intracellular TG content and the number of lipid droplets in PSC decreased with increasing lactic acid concentration. (Figure 2H,I). The induction effect of lactate on the PSCs activation phenotype was similar to that of the classical PSCs activation inducer TGF‐β1.

### 2.3 MCT1 is a Specific Transporter Mediating Lactate‐Induced PSCs Activation

**FIGURE 3:**
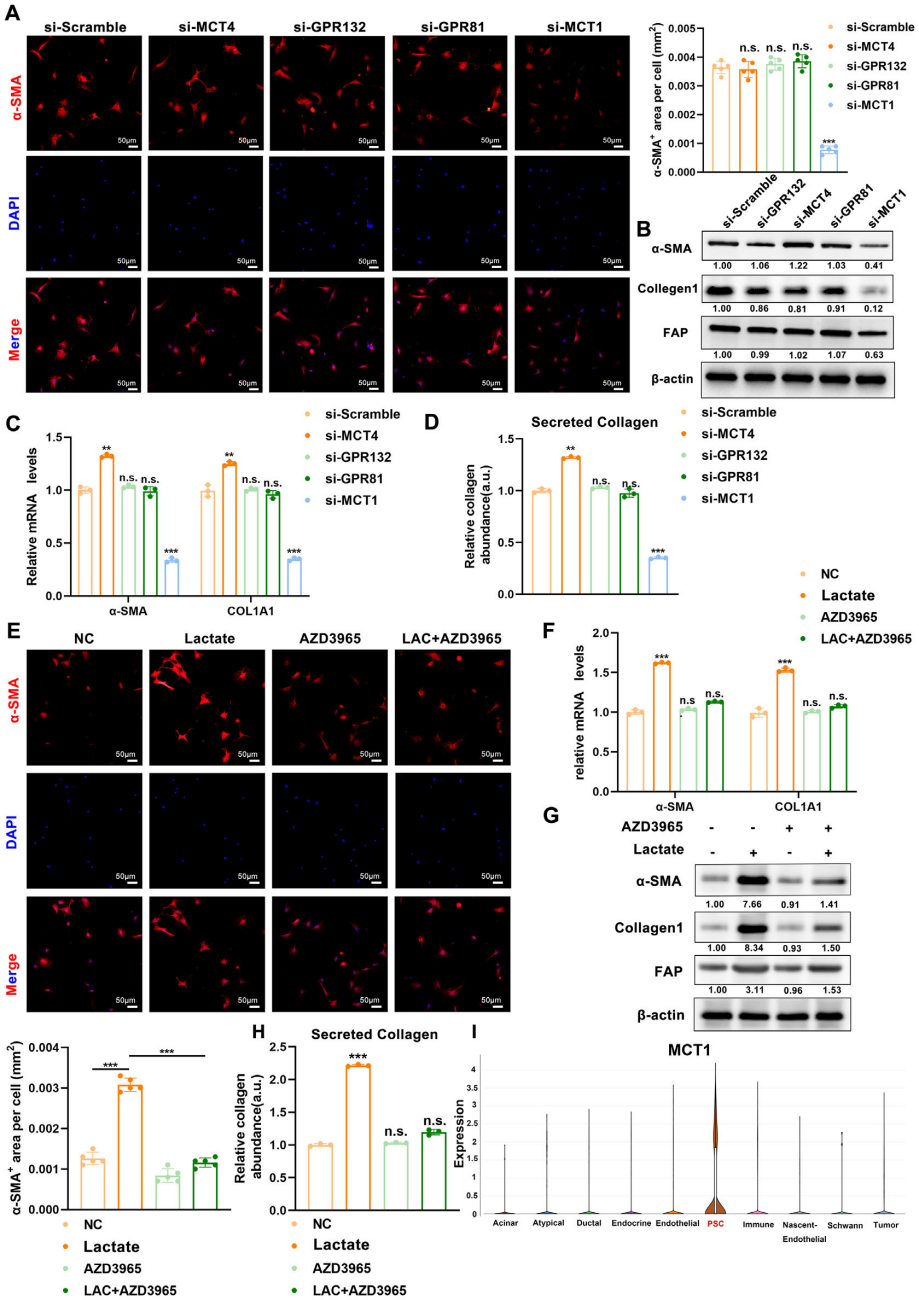
MCT1 is a specific transporter mediating lactate‐induced PSCs activation. (A) Representative α‐SMA fluorescence staining images of primary mouse PSCs treated with si‐Scramble, si‐MCT4, si‐GPR132, si‐GPR81, or si‐MCT1 transfection under LAC 15 mm culture conditions for 3 days. Nuclei stained by DAPI. Scale bars: 50 µm. α‐SMA positive area of individual PSCs (*n* = 5). (B) Western blot analysis of α‐SMA, Collagen I, and FAP expression in the PSCs (*n* = 3). (C) The mRNA levels of α‐SMA and COL1A1 were measured by quantitative RT‐PCR assays (*n* = 3). (D) Collagen I in the supernatant of the culture medium was detected using the ELISA kit (*n* = 3). (E) Representative α‐SMA immunofluorescence staining images of primary mouse PSCs with or without lactate and AZD3965 treated for 3 days. Nuclei was stained by DAPI. Scale bars: 50 µm. α‐SMA positive area of individual PSCs (*n* = 5). (F) The mRNA levels of α‐SMA and COL1A1 of PSCs were measured by quantitative RT‐PCR assays (*n* = 3). (G) Western blot analysis of α‐SMA, Collagen I, and FAP expression in the PSCs (*n* = 3). (H) Collagen I in the supernatant of PSCs culture medium detected by using an ELISA kit (*n* = 3). (I) Single‐cell violin plot of MCT1 expression in human PC patients. Data are mean and ± SEM. ^***^ *p* < 0.001; n.s., not significant. Statistical significance was determined by one‐way ANOVA or two‐sided Student's t‐test as appropriate.

Next, we investigated the mechanism by which lactate activates PSCs. Lactate is sensed via G protein‐coupled receptors 132 and 81 (GPR132 and GPR81) [17, 18]; and monocarboxylate transporter 1 and 4 (MCT1 and MCT4) [19]. To determine the main receptor mediating lactate‐induced activation of PSCs, each receptor was knockdown with small interfering RNA (siRNA) and treated with lactate (15 mm) separately (Figure S2A,B). IF assay and lactate content measurement displayed that only MCT1 knockdown(si‐MCT1) significantly reduced lactate‐induced α‐SMA expression and intracellular lactate content in PSCs (Figure 3A,B). Western blot and qPCR analysis also confirmed the lactate‐induced overexpression of α‐SMA and Collagen I was significantly reversed in MCT1‐knockdown PSCs (Figure 3B,C). Moreover, only knockdown of MCT1 distinctively decreased Collagen I secretion in PSCs induced by lactate (Figure 3D). To further verify whether MCT1 is the critical receptor for lactate‐mediated activation of PSCs, PSCs were treated with selective MCT1 inhibitor AZD3965. Similar to MCT1 knockdown, AZD3965 remarkably inhibited the lactate‐induced PSCs activation and collagen I secretion (Figure 3E–H). Importantly, single‐cell data from PC databases (PRJCA001063) showed that MCT1 is primarily expressed in activated PSCs (Figure 3I).

Moreover, immune analysis of TIMER2.0 and GEPIA database demonstrated that MCT1 expression was negatively correlated with CD8^+^ T cell infiltration and patients’ prognoses, but positively correlated with M2 macrophage and MDSC infiltration, as well as expression of PSCs marker COL1A1, ACTA2 (Figure S2D–F). To further determine the expression localization of MCT1 in PSCs, we performed Western blot and confocal imaging, and found that MCT1 is mainly expressed on the cell membrane (Figure S2G,H). These findings suggest that MCT1 might act as a pro‐tumorigenic factor in PC, associated with immune suppression in the TME, highlighting the importance of targeting MCT1 in PSCs.

In conclusion, lactate primarily enters PSCs through the MCT1 transporter on the cell membrane, leading to their activation.

### 2.4 Lactate Activates PSCs by Inducing Autophagy

**FIGURE 4:**
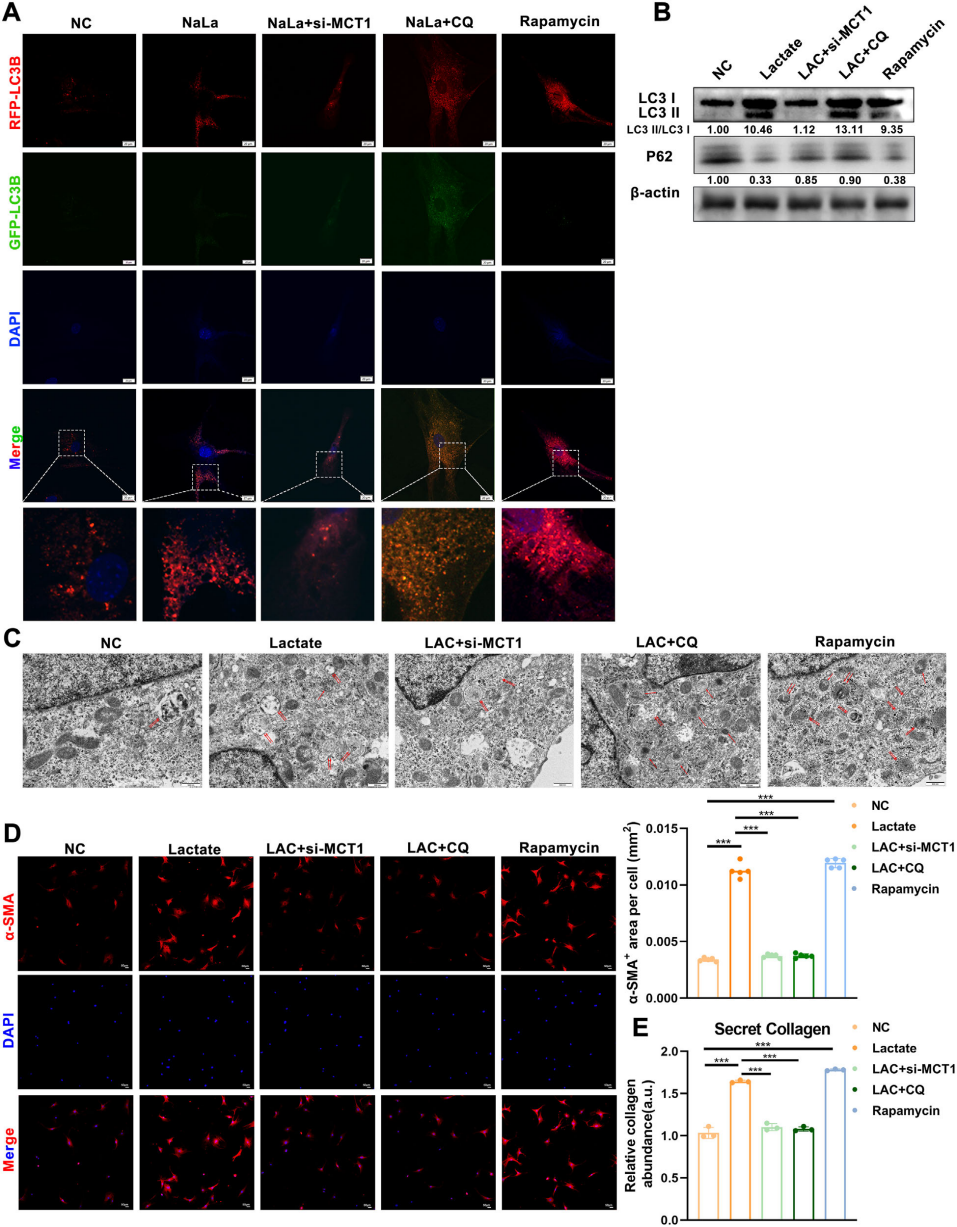
Lactate activates PSCs by inducing autophagy. (A) Representative confocal microscopy images of the red‐only puncta and the yellow puncta in PSCs (*n* = 3). PSCs Nuclei was stained with DAPI. Scale bar: 20 µm. (B) Western blot analysis of LC3 I/II and p62 expression in PSCs (*n* = 3). (C) Transmission electron microscopy of the autophagosome and autolysosomes of PSCs (*n* = 3). Scale bars: 500 nm. Double arrow: autophagosome, single arrow: autolysosomes. (D) Representative fluorescence microscopy images of PSC after α‐SMA immunostaining. Scale bars: 10 µm. And α‐SMA positive area of individual PSC (*n* = 5). (E) Collagen I in the supernatant of the PSCs culture medium detected by using an ELISA kit (*n* = 3). Data are mean and ± SEM. ^***^p < 0.001. Statistical significance was determined by one‐way ANOVA or two‐sided Student's t‐test as appropriate.

Having established that lactate exerts its effects by entering PSCs, we further investigated the specific mechanism of lactate‐induced PSCs activation. Our previous research proved that PSCs can be activated through autophagy [20] and Endo S, et al. found that genetic and chemical autophagy inhibition leads PSCs to a quiescent state [21]. To assess the effects of lactate on PSCs autophagy, we transduced primary PSCs with the stubRFP‐sensGFP‐LC3 reporter lentivirus to monitor changes in autophagic flux [22]. The results showed that sodium lactate (NaLa)‐used to rule out the effect of lactic acid pH on the lentivirus, significantly increased red LC3B‐positive puncta but not green LC3B‐positive puncta in PSCs which was similar to the effect of rapamycin (RAP), an autophagy initiator that inhibits mTOR [23]. However, the pretreatment with chloroquine (CQ), an inhibitor of autophagosome and lysosome fusion, distinctively elevated both red and green LC3B‐positive puncta in PSCs treated with lactate, which was shown as markedly increased yellow puncta in the merged image. Moreover, si‐MCT1 significantly suppressed the lactate‐induced accumulation of red LC3B‐positive puncta (Figure 4A). These results further confirmed that MCT1‐mediated lactate flow induced autophagosome formation in PSCs, similar to the effect of RAP, and this induction was blocked by CQ. Western blot analysis further demonstrated that both lactate and RAP significantly increased the LC3II/LC3I ratio, while significantly reducing the levels of the autophagy substrate p62. Notably, these effects were abolished by MCT1 knockdown, suggesting that MCT1 plays a critical role in mediating the effects of lactate on PSC autophagy (Figure 4B). Moreover, transmission electron microscopy displayed an increased number of autolysosomes in PSCs after lactate and rapamycin treatment, which was markedly inhibited by si‐MCT1. However, pretreatment with CQ apparently decreased the number of autolysosomes, but increased the number of autophagosomes (Figure 4C). Both lactate and RAP significantly increased α‐SMA expression and Collagen I secretion of PSCs, which was reversed by pretreatment with CQ (Figure 4D,E). Together, these results suggested that lactate activates PSCs in an autophagy‐dependent manner.

### 2.5 Lactate Induces Autophagy in PSCs by Regulating Vps34 Lactylation

**FIGURE 5:**
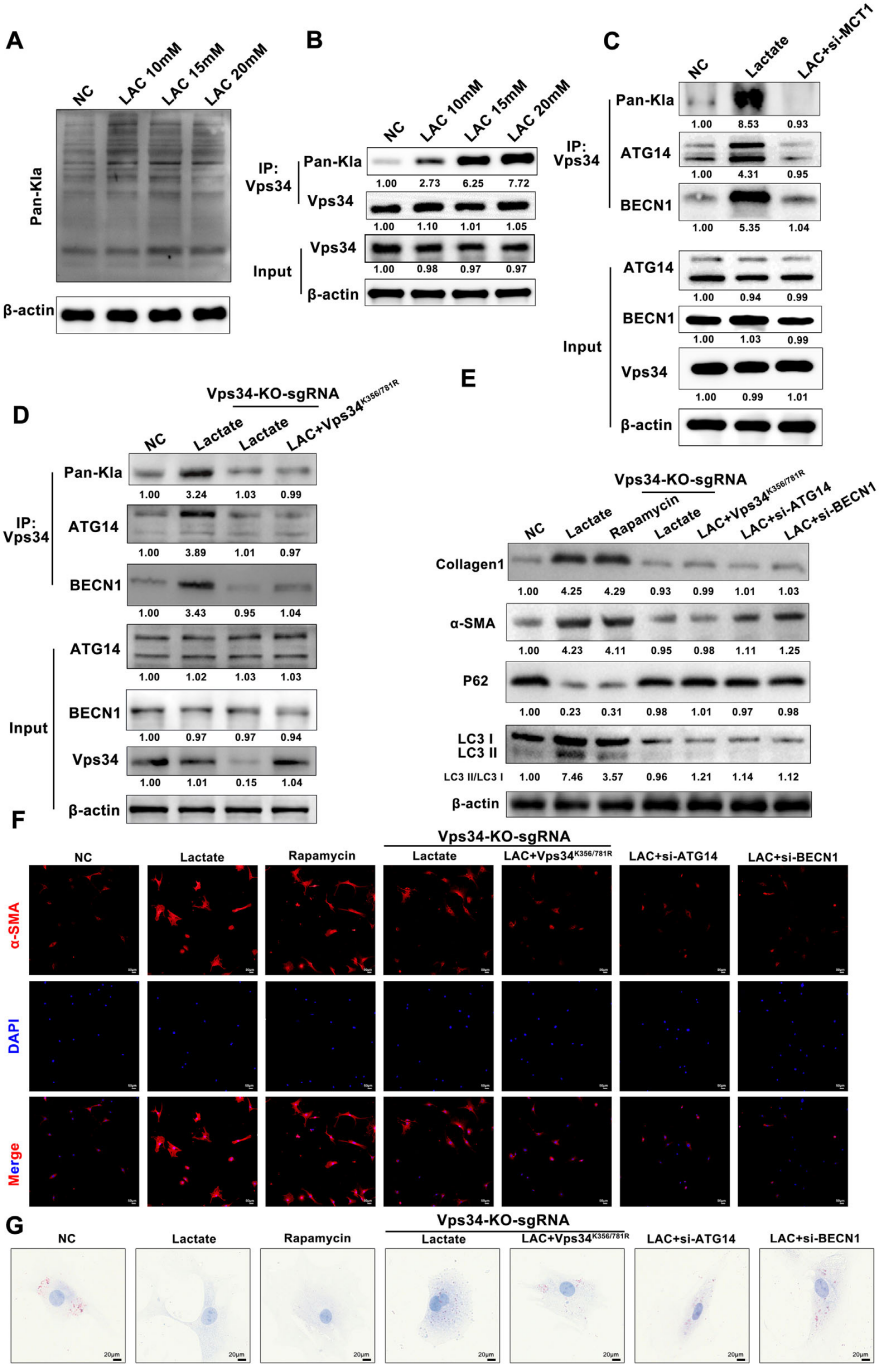
Lactate induces autophagy in PSCs by regulating Vps34 lactylation. (A) Western blot analysis of pan‐Kla levels in PSC under normal condition, LAC 10 mm, LAC 15 mm, or LAC 20 mm for 3 days (*n* = 3). (B) Western blot analysis of Vps34 and Pan‐Kla levels bound to Vps34 in PSCs (*n* = 3). (C) Western blot analysis of ATG14, BECN1, Vps34, and the levels of pan‐Kla, ATG14, and BECN1 bound to Vps34 in PSCs with lactate treated or si‐MCT1 transfected (*n* = 3). (D) Western blot analysis of ATG14, BECN1, Vps34, and the levels of pan‐Kla, ATG14 and BECN1 bound to Vps34 in PSCs with or without lactate and rapamycin treated, Vps34‐knockout‐sgRNA, Vps34‐knockout‐sgRNA + Vps34‐K^356/K781R^, si‐ATG14, si‐BECN1 transfected (*n* = 3). (E) Western blot analysis of Collagen I, α‐SMA, p62, and LC3 expression levels in PSCs with the above treated (*n* = 3). (F) Representative fluorescence microscopy images of PSCs with the above treated (*n* = 3) after α‐SMA immunostaining. Scale bars: 50 µm. (G) Representative images of lipid droplets in PSCs stained with Oil Red O, with nuclei stained by hematoxylin (*n* = 3). Scale bar: 20 µm.

Vps34 is fundamentally a lipid kinase, and its lactylation promotes interactions with BECN1 and ATG14, forming the core autophagy complex, enhancing its lipid kinase activity, and promoting autophagosome formation [24]. Since research demonstrated that lactylation of lysine residues K356 and K781 on Vps34 enhances its recruitment of BECN1 and ATG14 and then initiates the autophagy process [24], therefore, we further explored whether lactate induced autophagy in PSCs via lactylation of Vps34. To evaluate the global lactylation level of PSCs, Western blot analysis with a pan‐lactylation antibody was performed. The results showed that lactate supplementation increased global lactylation level in PSCs (Figure 5A). Next, Co‐IP analysis showed that lactate markedly increased the lactylation level of Vps34 (Figure 5B), as well as promoted the recruitment of Vps34 on ATG14 and BECN1 which was distinctively inhibited by si‐MCT1 (Figure 5C). To further identify the effect of Vps34 lactylation on lactate‐induced autophagy in PSCs, Vps34 was knocked out using CRISPR‐Cas9, followed by lentivirus‐mediated overexpression of Vps34 with mutations at the K356 and K751 sites. The co‐IP results verified the lactate‐enhanced interaction of Vps34 with ATG14 and BECN1 was significantly impaired in the PSCs with Vps34 knockout or Vps34 knockout followed by overexpressed of Vps34^K356/781R^ (Figure 5D). Moreover, the results of Western blot, IF of α‐SMA, ELISA of collagen I, and Oil Red O staining of PSCs lipid droplets also indicated that the lactate‐induced autophagy and activation of those PSCs were distinctively reversed, demonstrating with decreased expression of Collagen I, α‐SMA, LC3II/I, as well as increased the expression of p62 and accumulation of lipid droplets in those pretreated (Figure 5E–G and Figure S3A). Continually, these cells were transduced with stubRFP‐sensGFP‐LC3 reporter lentivirus and observed under confocal microscopy. The results displayed that the NaLa‐induced red LC3B‐positive puncta was also significantly decreased in those pretreated PSCs (Figure S3B).

In summary, we confirmed the importance of Vps34 lactylation in lactate‐induced autophagy initiation and activation in PSCs.

### 2.6 Development of Orthotopic Pancreatic Cancer was Impaired in PSC‐MCT1^−/−^ Mice

**FIGURE 6:**
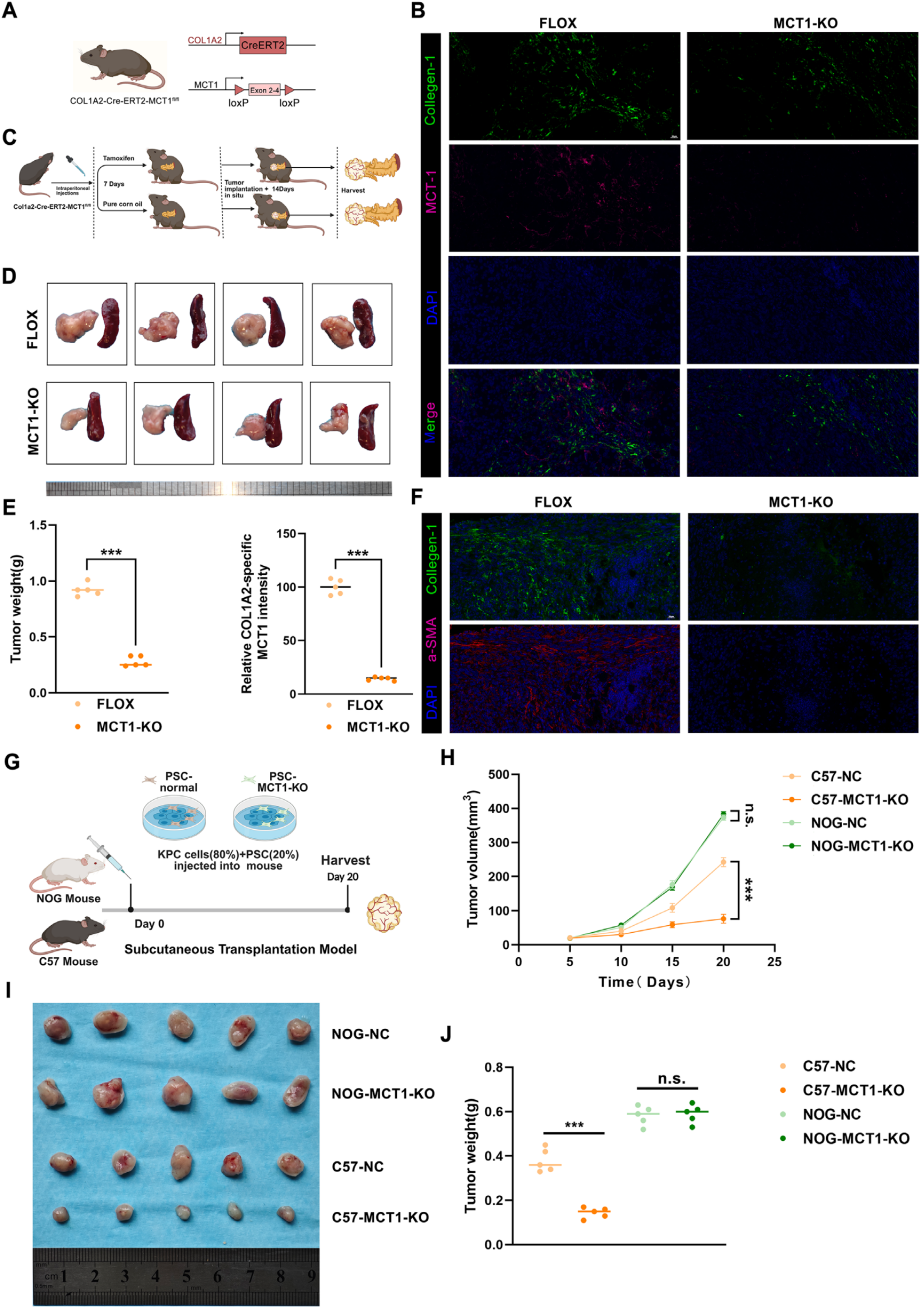
Development of orthotopic pancreatic cancer was impaired in PSC‐MCT1^−/−^ mice. (A) Gene Construction Strategy for COL1A2‐Cre‐ERT2‐MCT1^−/−^ Mice. (B) Double Immunofluorescence Staining of Collagen I and MCT1 in MCT1^−/−^ and MCT1^fl/fl^ mice. Nuclei are stained with DAPI. Scale bar: 20 µm. Relative MCT1 fluorescence intensity of Collagen I positive area. (C) Strategy for Constructing Conditional MCT1^−/−^ mice with PC in situ. Tamoxifen or corn oil is injected for 7 days, followed by the injection of KPC cells into the pancreas. In situ tumors were harvested 14 days later. (D–F) Representative Images of in situ tumors (*n* = 4) in Figure 6C (D), tumor weight statistics (*n* = 5) (E), and double immunofluorescence observation of Collagen I and α‐SMA in tumors, with nuclei stained by DAPI (*n* = 3) (F). Scale bar: 20 µm. (G) Diagram of the in vivo experimental regimen. (H–J) Tumor volumes (H) and representative Images of in situ tumors (*n* = 5) (I) in Figure 6G and tumor weight statistics (*n* = 5) (J). Data are mean and ± SEM. ^***^ *p* < 0.001; n.s., not significant. Statistical significance was determined by one‐way ANOVA or two‐sided Student's t‐test as appropriate.

To further investigate the specific effects of lactate‐activated PSCs on PC cells, Pdx1‐cre/LSL‐Kras G12D/P53(R172H) (KPC) cells were co‐cultured with PSCs pretreated with lactate (PSCs^LAC^) (Figure S4A). The results showed that PSCs^LAC^ significantly enhanced the migratory and invasive abilities of KPC cells, which was significantly inhibited by MCT1 knockdown in PSCs^LAC^ (Figure S4B). Nevertheless, the colony formation assays revealed the proliferation of KPC cells was not affected by PSCs^LAC^ (Figure S4C). To further reveal the effect of lactate‐activated PSCs on PC in vivo, we utilized the Cre‐Lox recombination system to generate conditional MCT1 knockout mice targeting PSCs (PCSs‐MCT1^−/−^ mice) (Figure 6A). *Col1a2* was chosen as it is a marker gene for PSCs in the CellMarker database (Figure S4D).

After constructing the model, we extracted DNA from mouse tails and performed DNA gel electrophoresis to verify the expected genotype (Figure S4E). After 4 weeks, mice were sacrificed to extract primary PSCs for Western blot and PCR analysis, confirming significantly reduced protein and RNA levels of MCT1 in PSCs (Figure S4F,G). Additionally, immunofluorescence co‐staining for Collagen I and MCT1 in pancreatic tissue sections visually demonstrated the specific knockout of MCT1 in PSCs (Figure 6B).

Upon confirming the successful construction of the MCT1 conditional knockout model, we orthotopically injected KPC cells into the pancreases of knockout and control mice to establish an orthotopic cancer model (Figure 6C). Two weeks after injection, the tumors in PSC‐MCT1^−/−^ mice were significantly smaller and lighter than those in flox mice. (Figure 6D,E). Immunofluorescence staining for Collagen I and α‐SMA, as well as immunohistochemistry, indicated significantly reduced PSCs activation in the pancreatic section of PSC‐MCT1^−/−^ mice (Figure 6F and Figure S4H), which was further verified by Sirius Red and Masson's trichrome staining. Moreover, the liver metastasis of PSC‐MCT1^−/−^ mice was markedly inhibited compared to that in flox mice (Figure S4I).

In vivo experiments demonstrated that MCT1^−/−^ mice could significantly slow tumor growth and suppress liver metastasis of PC. These results are consistent with the effect of PSCs on the invasion and migration ability of PC cells in the in vitro co‐culture test, but not with the effect of PSCs on the proliferation ability of PC. Therefore, we hypothesized that active PSCs might not directly enhance the proliferation of tumor but indirectly via modulating the immune TME. To confirm this presumption, we established subcutaneous co‐xenograft tumor models mixing KPC cells and PSCs treated with or without si‐MCT1 in immunocompetent C57 mice or immunodeficient NOG mice, respectively (Figure 6G). Compared to mixing with normal PSCs, the growth of implanted tumor mixing with MCT1‐ knockdown PSCs was distinctively inhibited in C57 mice, but not in NOG mice (Figure 6H–J). These results further suggest that lactate‐activated PSCs contributes to the tumorigenesis of PC mainly via manipulating the immune TME.

In summary, we demonstrated that PSCs‐specific MCT1 knockout significantly reduces the malignancy and fibrosis levels in a mouse model of orthotopic PC by regulating the immune TME.

### 2.7 Activated PSCs Facilitates Immunoevasion TME via Inducing PD‐1 Expression of CD8^+^ T Cells

**FIGURE 7:**
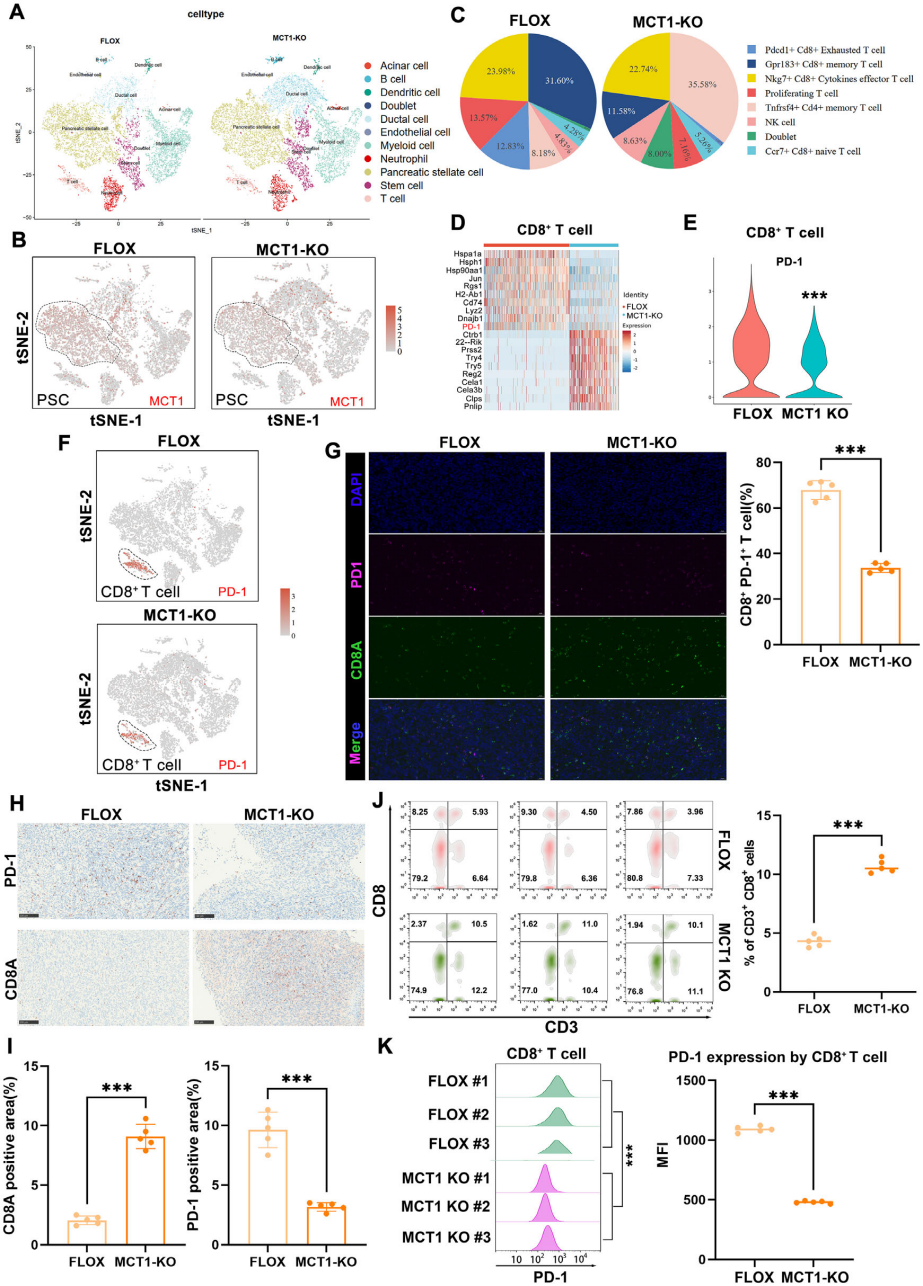
Activated PSCs facilitates immunoevasion TME via inducing PD‐1 expression of CD8^+^ T cells. (A) Single‐cell sequencing analysis of unsorted live cell mixtures from in situ tumors of MCT1^−/−^ and MCT1^fl/fl^ mice. (B) T‐SNE plots of MCT1 expression levels by single‐cell analysis. (C) Proportional composition of T cell subpopulations in two groups from single‐cell analysis. (D) Heatmap of differential gene analysis in CD8^+^ T cells. (E) Relative expression levels of PD‐1 in CD8^+^ T cells in the two samples. (F) T‐SNE plots of PD‐1 expression levels by single‐cell analysis. (G) Dual immunofluorescence labeling of PD‐1 and CD8A in mouse PC samples with statistical analysis of CD8^+^ PD‐1^+^ cell numbers (*n* = 5). Scale bars: 20 µm. (H‐I) Immunohistochemical analysis of PD‐1 and CD8A levels in mouse PC samples (H) with statistical analysis of positive areas (*n* = 5) (I). Scale bars: 100 µm. (J) Flow cytometric analysis of CD3^+^ CD8^+^ cell numbers in the two samples with statistical analysis (*n* = 5). (K) Flow cytometric analysis of PD‐1 expression levels in CD8^+^ T cell populations sorted from in situ tumor samples with statistical analysis (n = 5). Data are mean and ± SEM. ^***^ *p* < 0.001. Statistical significance was determined by one‐way ANOVA or two‐sided Student's t‐test as appropriate.

To further investigate the mechanism by which activated PSCs regulate the immune TME, we performed single‐cell RNA sequencing (scRNA‐seq) on dispersed primary PC samples. After cell type annotation using marker genes, we classified over 10 000 cells (Figure S5A) and visualized them using t‐SNE plots and bar graphs (Figure 7A and Figure S5B). The t‐SNE plots revealed that the MCT1 expression was significantly lower in PSCs from PSCs‐MCT1^−/−^ mice compared to those from flox mice, confirming successful model construction and MCT1 specificity in PSCs as previously described (Figure 7B and Figure S5C).

We then focused on immune cell interactions. Recent studies have demonstrated that CAFs in PC secrete CXCL5 following Collagen I deposition to recruit MDSCs and suppress CD8^+^ T cells [5]. Moreover, HIF2 knockout in CAFs significantly reduces tumor fibrosis and markedly decreases the infiltration of immunosuppressive M2 macrophages and regulatory T cells [6]. Based on these findings, we hypothesized that PSCs might exert their effects through multiple immune cell populations.

Analysis of the proportion of T cell subpopulations showed that, compared to the PSCs‐MCT1^−/−^ mice, the number of different PD1^+^ CD8 T^+^ cells in the control group mice was significantly increased (Figure 7C and Table S5). We further compared immune cell‐specific gene expression differences between the two groups (Tables S3 and S4). Notably, the expression level of immune checkpoint including programmed death‐1 (PD‐1) and Signal regulatory protein alpha (SIRPα) in PSCs‐MCT1^−/−^ mice was significantly decreased in CD8^+^ T cells (Figure 7D–F) and myeloid cells (Figure S5D,E) separately. Pathway analysis of differentially expressed genes further revealed functional alterations in CD8^+^ T cells (Figure S5F, Tables S6 and S7). Furthermore, we prioritized CD8^+^ T cell analysis with IF, IHC, and flow cytometry, which confirmed the increased CD8^+^ T cell infiltration and decreased PD‐1 expression in PSCs‐MCT1^−/−^ group (Figure 7G–K). Further flow cytometry analysis of CD8^+^ T cell cytotoxicity revealed that the number of effector CD8^+^ T cells expressing GzmB^+^ and IFN‐γ^+^ was significantly increased in the PSCs‐MCT1^−/−^ group (Figure S5G).

Meanwhile, bioinformatic analysis using the TIMER2.0 database revealed a negative correlation between COL1A1/ACTA2 and CD8^+^ T cell infiltration, but a positive correlation with M2 macrophage accumulation (Figure S5H). This means clinical data further supported this association between PSCs activation and immune cell dynamics.

Collectively, these results indicate that the lactate‐activated PSCs might induce an immunosuppressive TME via increasing PD‐1 expression on CD8^+^ T cells.

### 2.8 Activated PSCs Upregulates PD‐1 Expression in CD8^+^ T Cells via CXCL9/CXCL10‐CXCR3‐STAT3 Axis

**FIGURE 8:**
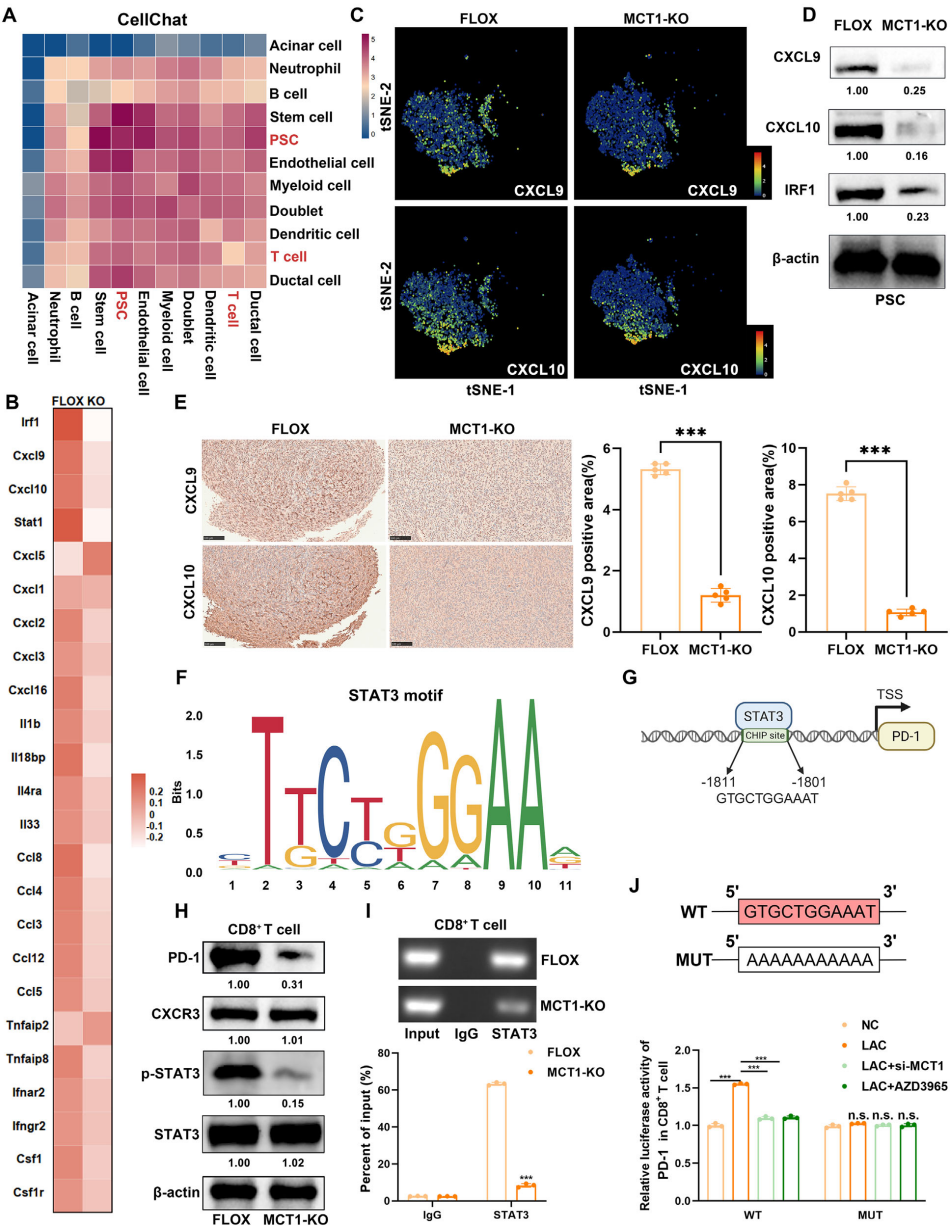
Activated PSCs upregulates PD‐1 expression in CD8^+^ T cells via the CXCL9/CXCL10‐CXCR3‐STAT3 axis. (A)The number of statistically significant ligand receptor expressions between various cell populations after single‐cell analysis. (B) Heatmap of differential gene expression in PSCs from single‐cell sequencing. (C) T‐SNE plot of CXCL9 and CXCL10 expression levels in the PSCs population by single‐cell analysis. (D) Western blot analysis of CXCL9, CXCL10, and IRF1 expression levels in mice PC samples (*n* = 3). (E) Immunohistochemical detection of CXCL9 and CXCL10 in mice PC samples (*n* = 5). Scale bars: 100 µm. (F,G) Schematic diagram of the potential binding sites for the STAT3 motif (F) in the promoter region of PD‐1 (G) by using the JASPAR database. (H) Western blot analysis of PD‐1, CXCR3, p‐STAT3, and STAT3 expression levels in CD8^+^ T cells from two sample groups (*n* = 3). (I) ChIP‐PCR assay with STAT3 antibody or IgG was conducted to explore the binding between STAT3 protein and binding sites of PD‐1 promoter in CD8^+^ T cells from two sample groups, and visualization on 2% agarose gel (*n* = 3). (J) Schematic representation of the mutant sequence. Luciferase reporter assays show the relative activity of PD‐1 promoter in CD8^+^ T cells containing WT or MUT reporter vector. These CD8^+^ T cells were derived from co‐culture with different treated PSC (with or without lactate, AZD3965 treated and si‐MCT1 transfected) (*n* = 3). Data are mean and ± SEM. ^***^ *p* < 0.001. Statistical significance was determined by one‐way ANOVA or two‐sided Student's t‐test as appropriate.

As previously established, fibroblast activation can influence immune cell function through multiple mechanisms including cytokine signaling, ligand‐receptor communication, and extracellular matrix remodeling [25]. To investigate cell communication in this context, we analyzed interaction strength between major cell types using ligand‐receptor expression profiles derived from single‐cell sequencing data. Our findings revealed relatively strong communication between PSCs and various cell populations, with PSCs exhibiting particularly high interaction intensities with T cells (Figure 8A and Tables S8 and S9).

Furthermore, differential gene expression analysis of cytokines in PSCs identified significant upregulation of Irf1 and its downstream cytokines CXCL9 and CXCL10 in the control group (Figure 8B and Table S10). This phenomenon was more pronounced in t‐SNE plot visualization (Figure 8C) and confirmed through Western blot of extracted PSCs and IHC analysis of mouse tumor tissues (Figure 8D,E).

Recent studies have demonstrated that CXCL9 and CXCL10 are recognized by CXCR3 on T cells [26], activating phosphorylation of STAT family members [27]. Notably, phosphorylated STAT3 has been shown to regulate PD‐1 expression in T cells through promoter binding, driving CD8^+^ T cell exhaustion [28, 29]. Additionally, analysis using the JASPAR database predicted a STAT3 binding site (MA0144.2) at positions −1811 to −1801 of the PD‐1 transcription start site (Figure 8F,G). Consistently, both mRNA and protein level of Irf1, CXCL9 and CXCL10 in primary PSCs were significantly increased under lactate supplementation, which was markedly inhibited by pretreatment with si‐MCT1 or Vps34 knockout followed by overexpressed of Vps34^K356/781R^ (Figure S6A,B). Secreted CXCL9 and CXCL10 levels in PSCs supernatants showed similar trends (Figure S6C). Therefore, we hypothesized that the lactate‐activated PSCs regulate PD‐1 expression on CD8^+^ T cells through the CXCL9/CXCL10‐CXCR3‐STAT3 axis. We then performed flow cytometric sorting of CD8^+^ T cells from model mice followed by Western blot and ChIP‐PCR analysis. The results showed a significant reduction of p‐STAT3 and PD‐1 protein levels in the MCT1‐knockout group, while CXCR3 and total STAT3 levels remained unaffected (Figure 8H). Furthermore, the ChIP assay revealed that p‐STAT3 was enriched on the PD‐1 promoter in flox mice, an effect that was significantly decreased in PSCs‐ MCT1^−/−^ mice (Figure 8I).

To further verify the effects of lactate‐activated PSCs on CD8^+^ T cells, the CD8^+^ T cells were co‐cultured with PSCs pretreated by lactate, accompanied with si‐MCT1 or Vps34 knockout followed by overexpressed of Vps34^K356/781R^ (Figure S6D). After co‐cultured with pretreated PSCs, CD8^+^ T cells were transfected with a luciferase reporter plasmid containing wild type (WT) or mutant (MUT) promoter sequence of PD‐1 separately. The results demonstrated the luciferase activity in WT CD8^+^ T cells was significantly enhanced by co‐culture with lactate‐pretreated normal PSCs, which was significantly inhibited by accompanying with si‐MCT1 or inhibitor AZD3965. Nevertheless, co‐culture with pretreated‐PSCs did not affect luciferase activity in CD8^+^ T transfected with MUT (Figure 8J). Coincidently, lactate‐activated PSCs remarkably upregulated PD‐1, p‐STAT3 protein levels and luciferase activity of PD1 in CD8^+^ T cells, which were reversibly abrogated by MCT1 knockdown or Vps34 knockout followed by overexpressed of Vps34^K356/781R^ (Figure S6E,F). Furthermore, to verify the effect of autophagy factors other than Vps34 on the activation function of PSCs, we knocked down Atg5 and Atg7 and treated PSCs with lactate or rapamycin, we found that compared with the group treated with lactate and rapamycin, the pretreatment of knocking down ATG5 and ATG7 could significantly downregulate the expression of CXCL9, CXCL10, and IRF1in PSCs. (Figure S6G). Co‐culturing these treated PSCs with CD8^+^ T cells also showed that inhibiting the autophagy pathway can eliminate the effect of activated PSCs on PD1 upregulation in CD8^+^ T cells (Figure S6H). To verify the downstream pathways in CD8^+^ T cells, we performed knockdowns of CXCR3 and STAT3, and found that the effects of lactate‐activated PSCs on CD8^+^ T cells were reversed by the knockdown of either CXCR3 or STAT3 (Figure S6I,J). In addition, we used human‐derived PSCs and CD8^+^ T cells for functional co‐culture assays, and the results were highly consistent with those of the mouse‐derived cell lines (Figure S7A–J), further indicating the possibility of clinical translation for this finding.

Collectively, these findings intensively indicate that lactate‐activated PSCs increased PD‐1 expression in CD8^+^ T cell via the CXCL9/CXCL10‐CXCR3‐STAT3 axis. This mechanism represents a critical pathway through which lactate‐activated PSCs modulate the immunosuppressive tumor microenvironment.

### 2.9 AZD3965 Sensitizes Orthotopic Pancreatic Cancer to PD‐1 Blockade to Restrain Tumor Progression in Mice

**FIGURE 9:**
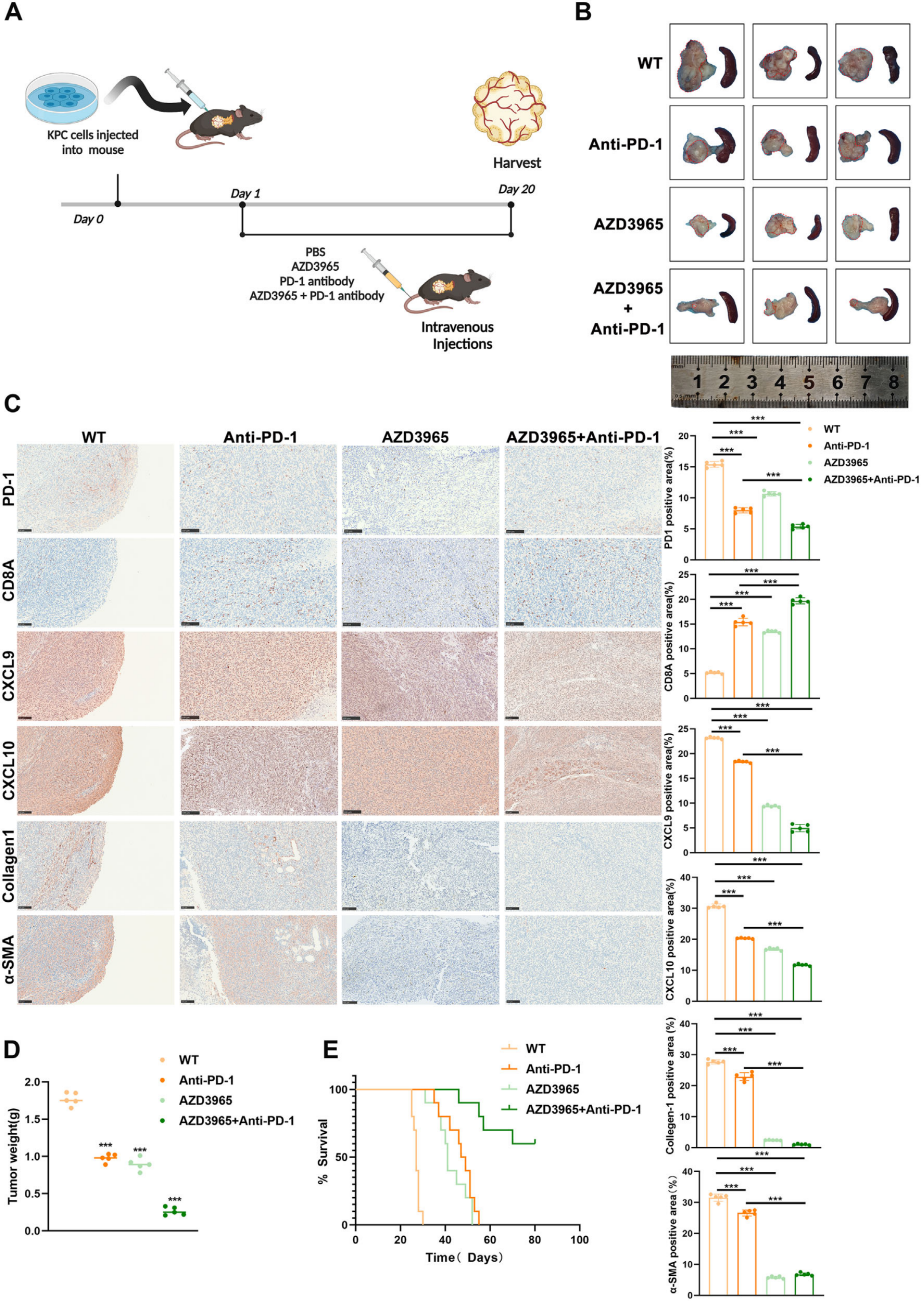
AZD3965 sensitizes orthotopic pancreatic cancer to PD‐1 blockade to restrain tumor progression in mice. (A–D) Strategy for constructing and treating the pancreatic cancer mouse model in situ (A). KPC cells are injected into the pancreas, followed by daily tail vein injections of PBS, AZD3965 (100 mg/kg, once every three days), Anti‐mouse PD‐1 antibody (10 mg/kg, once every three days), or AZD3965 + Anti‐mouse PD‐1 antibody. Tumors are harvested after 20 days (B). Immunohistochemical analysis of PD‐1, CD8A, CXCL9, CXCL10, Collagen I, and α‐SMA expression levels with statistical analysis (*n* = 5) (C). Tumor weights are measured (*n* = 5) (D). Scale bars: 100 µm. (E) Survival curves of mice with in situ pancreatic cancer under different treatment conditions (*n* = 10). Data are mean and ± SEM. ^***^ *p* < 0.001. Statistical significance was determined by one‐way ANOVA or two‐sided Student's t‐test as appropriate.

The above experimental results indicate that lactate‐induced activation of PSCs not only contributes to dense stromal deposition in PC tissue but also promotes the secretion of cytokines that inhibit CD8^+^ T cells. The former creates a fibrotic physical barrier that hinders drug delivery, while the latter directly fosters an immunosuppressive microenvironment in PC. Therefore, we investigated whether the MCT1‐specific inhibitor AZD3965 could sensitize PC to anti‐PD‐1 antibody therapy to suppress tumor growth. KPC cells were orthotopically injected into mice pancreas and followed by treatment with PBS, AZD3965, Anti‐mouse PD‐1 antibody, a combination of AZD3965 and Anti‐mouse PD‐1 antibody, respectively (Figure 9A). After 3 weeks, the tumors were harvested for further evaluation. The results demonstrated that tumor growth was significantly inhibited by AZD3965 or Anti‐mouse PD‐1 antibody monotherapy, but further restrained by AZD3965 and Anti‐mouse PD‐1 antibody (Figure 9B). Compared to the control group, the IHC analysis showed that Anti‐mouse PD‐1 antibody decreased PD‐1 positive area, but increased CD8^+^ T cell infiltration, without effects on tissue fibrosis and CXCL9/CXCL10 expression. Meanwhile, AZD3965 alone markedly increased CD8^+^ T cell infiltration, as well as decreased tissue fibrosis and CXCL9/CXCL10 levels, which were further enhanced by combination with Anti‐mouse PD‐1 antibody (Figure 9C). Coincidently, AZD3965 sensitized the tumors to Anti‐mouse PD‐1 antibody, significantly inhibiting tumor growth and improved survival of mice with PC (Figure 9D,E).

In summary, these results indicate that AZD3965 sensitizes tumors to Anti‐mouse PD‐1 antibody, effectively inhibiting PC progression via modulating the fibrotic and immunosuppressive TME.

## 3 Discussion

Although research had revealed that lactate in TME facilitate the immune escape of various tumors, however, the precise role of lactate in immunosuppressive TME of PC has not fully elucidated yet. Innovatively, the present study dissected that the MCT1‐mediated influx of lactate induced autophagy and activation of PSCs via lactylation of the key autophagic protein Vps34, which consequently increased PD‐1 expression in CD8^+^ T cells via activating the CXCL9/CXCL10/CXCR3/STAT3 pathway. Importantly, we identified that the MCT1 inhibitor relieved the immunosuppressive TME in PC mice, thereby sensitizing the tumors to PD‐1 blockade to effectively inhibit PC growth. These findings advocate for the potential development of future clinical trials that incorporate MCT1 inhibitors in conjunction with immunotherapy for the treatment of PC patients.

As a monocarboxylate transporter, MCT1 primarily facilitates the influx of lactate into cells [30]. Current research on MCT1 in PC focuses mainly on its role in PC cells, such as lactate taken up by MCT1 could enhance PC cells viability by maintaining cellular redox homeostasis under conditions of nutrient stress [31]; lactate administration under glucose starvation developed drug resistance and induced the expression of the stemness marker Nestin and reprogramming factors (Oct4, KLF4, Nanog). This effect was abrogated by the MCT1/lactate‐uptake inhibitor 7ACC2 or MCT1 knock‐down [32]; and lactate transport mediated by MCT1 and CD147 complexes could control the malignant potential of PC cells associated with the Warburg effect [33]. However, we found that some single‐cell data reveal that MCT1 is primarily expressed in PSCs in PC, and unlike the relatively low proportion of PSCs in normal pancreatic tissue, activated PSCs occupy a significant proportion in PC. This indicates the need for further research on the regulatory mechanisms of MCT1‐mediated lactate uptake and its effect on PSCs in PC. Our research found that MCT1 is related to the remodeling of the immune microenvironment and is specifically highly expressed in PSCs. Our experiments in vitro confirmed that MCT1 mediates PSCs autophagy activation by promoting lactate flux.

Upon further co‐culturing activated PSCs with KPC cells, we realized that PSCs might not directly influence the proliferation of KPC cells. Consequently, we developed PSCs‐specific MCT1^−/−^ mice and constructed an in‐situ PC model by injecting KPC cells. The results indicated significant inhibition of pancreatic tumors upon conditional knockout of MCT1. To further investigate the mechanisms by which activated PSCs promote in situ tumor growth, we conducted single‐cell sequencing on dissociated cell suspensions from the in situ tumor model. The results suggested that, compared to the PSCs‐MCT1^−/−^ tumor model, the control group exhibited an immunosuppressive microenvironment. Key immune microenvironment components, CD8^+^ T cells and myeloid cells, showed increased expression of the immune checkpoint PD‐1 and phagocytic checkpoint SIRP‐α. This not only leads to their reduced vitality and progression towards exhaustion [34, 35] but also weakens their direct cytotoxic and phagocytic abilities against PC cells, ultimately resulting in uncontrolled proliferation and metastasis of PC to other organs.

Nearly all multi‐drug chemotherapy regimens, and even the latest immune checkpoint blockade (ICB) therapies, have failed to provide promising treatment solutions for PC patients [36, 37]. Tumor stromal therapies have been explored, including physical disruption of hypoxic stromal components by inhibiting the Sonic Hedgehog protein [38], selective fibroblast depletion [39], and recombinant human hyaluronidase [40], among others. These interventions effectively reduce stromal content, but the observed outcomes before and after clinical application have been completely opposite. From the perspective of tumor invasion, overly dense tumor stroma acts as a limiting factor for tumor invasion and metastasis [41]. Thus, targeting the stroma alone may accelerate cancer cell proliferation and spread. Combining stromal‐targeted therapies with immune‐targeted drugs is a logical approach: eliminating the extracellular matrix can enhance the penetration and complete infiltration of targeted drugs into all parts of the tumor tissue (especially areas with poor blood supply and high density), while also inhibiting the paracrine immunosuppressive effects of activated PSCs on the immune microenvironment.

Currently, a study is evaluating the combination therapy of RO6874281 (a FAP‐blocking antibody) with atezolizumab (a PD‐L1 antibody) across different solid tumors, with promising results observed in cervical cancer patients (NCT02627274) [42]. Another study demonstrated that constructing in situ PC tumors using RIK2‐knockdown KPC cells alleviated the fibrotic immunosuppressive microenvironment compared to the control group, significantly improving the efficacy of PD‐1 antibody therapy [43]. These findings demonstrate the inseparable relationship between the alleviation of the immunosuppressive microenvironment and the efficacy of ICB therapy. A phase I clinical trial of AZD3965 in patients with advanced tumors is currently underway, showing that AZD3965 is tolerable at doses that engage its target [44]. To assess the efficacy of AZD3965 alone and in combination with ICB therapy in PC, we conducted further in vivo mouse experiments. The results demonstrated that the combination of AZD3965 and PD‐1 blockade significantly reduced intertumoral ECM deposition and the expression levels of CXCL9 and CXCL10, while also markedly decreasing PD‐1 expression and enhancing CD8^+^ T cell infiltration. Ultimately, this resulted in significant tumor volume reduction and prolonged survival in the model mice. Although our single‐cell data analysis indicates that MCT1 is predominantly expressed by PSCs within the pancreatic cancer microenvironment, we must acknowledge the limited specificity of systemic pharmacologic MCT1 inhibition. Because MCT1 is also broadly expressed across multiple cellular compartments, the enhanced efficacy observed with systemic AZD3965 treatment in combination with PD‐1 blockade cannot be considered exclusively stromal‐mediated. Recent pharmacokinetic and systems biology studies indicate that AZD3965 also exerts direct tumor‐cell‐intrinsic effects; pharmacologic inhibition of MCT1 directly disrupts metabolic homeostasis and cellular function in tumor cells [45, 46]. Furthermore, lactate broadly modulates various immune populations beyond the PSCs‐CD8^+^ T cell axis. As previously reported, a highly glycolytic tumor microenvironment with abundant lactate promotes PD‐1 expression in regulatory T cells, further dampening anti‐tumor immunity [47]. Therefore, while our PSC‐specific MCT1 knockout model robustly isolates and validates the critical contribution of stromal MCT1 to the immunosuppressive niche, we do not rule out the direct effects of AZD3965 on other cell types. Instead, the overall therapeutic benefit of systemic AZD3965 in sensitizing tumors to PD‐1 blockade likely stems from a multifaceted mechanism that directly disrupts the metabolic homeostasis of malignant cells, mitigates lactate‐induced suppression in immune cells such as Tregs, and dismantles the supportive fibrotic stroma.

In conclusion, our findings reveal the critical role of lactate‐mediated autophagy activation in PSCs via MCT1 in regulating immune evasion responses and promoting PC progression. This may provide new insights into the causes of the immunosuppressive microenvironment in PC and potential therapeutic interventions.

## 4 Methods and Materials

### 4.1 Human Samples

All patients were from Union Hospital, Tongji Medical College, Huazhong University of Science and Technology, Wuhan, China. Informed consent was obtained from all patients prior to the experiment. Cancer and adjacent non‐cancerous tissues were collected from patients diagnosed with PC. All research procedures involving human samples were approved by the Ethics Committee of the Academic Medical Center of Huazhong University of Science and Technology and complied with the Declaration of Helsinki.

### 4.2 Mice

Col1a2‐CreERT2‐MCT1^−/−^ mice were purchased from Cyagen, and SPF‐grade C57BL/6 (8‐week‐old) mice were obtained from Beijing Vital River Laboratory Animal Technology Co., Ltd. Based on the characteristics of ERT2 activation and previous reports, we administered intraperitoneal injections of tamoxifen to the mice for 5–6 consecutive days before performing orthotopic tumor implantation, with each mouse receiving a dose of 40 mg/kg per injection (Tamoxifen dissolved in corn oil, 20 µg/ml). After the injection cycle, primary PSCs were extracted from the mice for DNA gel electrophoresis, Western blot analysis, PCR, and in situ immunofluorescence (red for MCT1, green for Col1) to assess the conditional knockout level of MCT1. Subsequently, pre‐prepared KPC cells (1 × 10^6^) were mixed into 40 µL of unpolymerized Matrigel and injected under the pancreatic capsule after laparotomy. Successful injection was indicated by the formation of a bleb. The mice were sacrificed 2–3 weeks post‐construction, and orthotopic tumors were photographed and collected. All experiments were conducted using male mice.

### 4.3 Singal Cell RNA‐seq Data Analysis of Human Pancreatic Cancer

The scRNA‐seq dataset PRJCA001063 of human PC was downloaded from the NGDC database. And through the Single Cell Portal (https://singlecell.broadinstitute.org/single_cell) to realize the visualization of gene expression in different Cell types.

### 4.4 Clinical Data Analysis of the Lactate Generation Pathway

The scoring analysis of the lactate production pathway (GOBP_LACTATE_METABOLIC_PROCESS) in PDAC and its relationship with immune cell infiltration were completed by the website (https://guolab.wchscu.cn/GSCA), and the patient data were derived from the TCGA database.

### 4.5 Lactate‐Mediated Intercellular Interaction Score

The single‐cell expression matrix and cell cluster annotation information for dataset PRJCA001063 were downloaded from the NGDC database. The genes related to the lactate synthesis pathway and the lactate transmembrane transport pathway were derived from two datasets in the GSEA database, namely GOBP_LACTATE_METABOLIC_PROCESS and GOBP_LACTATE_TRANSPORT. The AUCell algorithm [48] was adopted to calculate the scores of lactic acid synthesis activity and lactic acid transmembrane transport activity for each cell type. The score of the lactate generation pathway for each cell population was 𝜇𝑥 𝑠, the score of the lactate transport pathway was 𝜇𝑦 𝑟, and the cellular communication intensity score of lactate between the two cell populations was Sc, Sc = 𝜇𝑥 𝑠 ∗ 𝜇𝑦 𝑟. To ascertain the statistical significance of this score, the cell cluster annotation labels of each cell cluster were randomly shuffled 1000 times, and the lactate communication strength score between cell clusters was recalculated. These 1000 scores were utilized to establish a null distribution, and the *p*‐value of the arginine communication strength score was determined using the Permutation test [49].

### 4.6 Isolation of Primary PSCs

Primary PSCs were isolated from the previously described mice. Briefly, after 40 min of digestion in a digestion solution containing collagenase I and IV (5 mg/ml), DNase I (5 mg/ml), and 0.25% Trypsin‐EDTA, the pancreas was pipetted and filtered via a 70 µm cell mesh. After centrifugation(335.4xg, 3 min), the supernatant was taken for further culture in DMEM/F‐12 medium containing 20% fetal bovine serum (FBS). Continue changing the medium for three days until all the suspended cells are eliminated, leaving only the adherent cells. The purity of primary PSCs was assessed by vitamin A autofluorescence under a fluorescence microscope. Primary PSCs were cultured in 12‐well plates until confluent and then transferred to T25 flasks with DMEM/F‐12 medium containing 20% FBS, 100 U/mL penicillin, and 100 mg/mL streptomycin, which was changed every 3 days.

### 4.7 Cell Culture

Primary PSCs were obtained using the extraction method described above, and KPC cells were donated by the Pancreatic Surgery Research Group at Wuhan Union Hospital. Mouse CD8^+^ T cells were negatively selected from mouse spleen blood using magnetic beads. The principle involves labeling non‐target cells (non‐CD8^+^ T cells) with different biotin‐labeled monoclonal antibodies, followed by removal of these non‐target cells using streptavidin‐labeled magnetic beads, thereby isolating mouse CD8^+^ T cells. SPF‐grade C57BL/6 (8‐week‐old) mice were used. Detailed procedures can be found in the Mouse CD8^+^ T Cell Isolation Kit (Selleck China) manual. Human pancreatic stellate cells and peripheral blood CD8^+^ T cells were purchased from Shanghai Zhong Qiao Xin Zhou Biotechnology Co., Ltd. (Shanghai, China).

### 4.8 Cell Treatments

Primary PSCs were cultured in DMEM/F‐12 containing 20% FBS at 37°C in a humidified incubator with 5% CO_2_. KPC cells were cultured in DMEM containing 10% FBS at 37°C in a humidified incubator with 5% CO_2_. Specific siRNA and scrambled siRNA were synthesized by GenePharma (Shanghai, China) and transfected into PSCs using Lipofectamine 2000 according to the provided instructions. Lactate (10–20 µmol/L, MedChemExpress) or TGF‐β (MedChemExpress) was added to the PSCs culture medium to assess PSCs activation markers. To inhibit MCT1, PSCs were treated with AZD3965 (5 nmol/L, MedChemExpress) for 24 h. To evaluate the relationship between lactate and autophagy, primary PSCs were treated with chloroquine (20 µmol/L, MedChemExpress) or rapamycin (100 nmol/L, MedChemExpress) for 24 h. To study the role of Vps34 lactylation in regulating autophagy in vivo, recombinant adeno‐associated virus packaging Vps34^K356,781R^ (Vigenebio, China) was used to generate primary Vps34^K356,781R^ PSCs.

### 4.9 Western Blot Analysis

Ice‐cold PBS was employed to wash the cells 2–3 times. Then the RIPA lysis buffer supplemented with protease inhibitor Cocktail (1:50) and phenylmethyl sulfonyl fluoride (PMSF, 1:100) was added to lyse the cells on ice for 30 min. After that, the lysate was collected and centrifuged at 12 000 g for 15 min, and the supernatant was transferred into a new centrifuge tube. An appropriate amount of 5× sodium dodecyl sulfate‐polyacrylamide gel electrophoresis (SDS‐PAGE) protein loading buffer was added to the protein sample in a 1:4 ratio. Boil water for 10 min to denaturant and store at −20°C. Protein was separated using SDS‐PAGE gel, and subsequently transferred onto polyvinylidene fluoride membranes (PVDF, Millipore, USA). Membranes were blocked in 5% non‐fat milk for 1 h at room temperature. Then incubated with specific primary antibodies overnight at 4°C followed by the incubation of anti‐mouse or rabbit horseradish peroxidase (HRP)‐conjugated secondary antibodies (#7076S/7074S, CST, dilution 1:3000). Afterwards, the enhanced chemiluminescence assay (ECL, 34095, Pierce) was implemented to visualize the band signals, collected by the ChemiDoc XRS molecular imager system (Bio‐Rad, USA). Various primary antibodies are listed in the key resources table (KRT) of the supplemental materials.

### 4.10 Determination of Intracellular Lactate and Triglyceride Contents

Primary PSCs from each treatment group were collected and counted, and the culture medium was discarded. After washing with PBS, the cell pellets were resuspended in PBS and subjected to ultrasonic disruption in an ice‐water bath until the solution became clear. Subsequently, the homogenate was centrifuged at 1006.2xg for 10 min at 4°C, and the supernatant was collected for further analysis. The lactate and TG (triglyceride) contents in the supernatant were determined using commercial assay kits from Nanjing Jiancheng Bioengineering Institute(see KRT. All procedures were performed strictly according to the manufacturer's instructions, and absorbance was measured at 546 nm (Lactate) and 500 nm (TG) using a microplate reader. Finally, the intracellular levels of lactate and TG were normalized to the corresponding cell counts, and the results were expressed as nmol/10,000 cells and as fold changes relative to the control group.

### 4.11 Flow Cytometry

After harvesting orthotopic PC tumors, the tissue was digested and dissociated using trypsin, collagenase I, and IV to prepare a single‐cell suspension. The cells were then fixed with 1% paraformaldehyde for 30 min, washed, and permeabilized with 0.1% Triton X‐100 for 10 min. After washing, the cells were incubated with various flow cytometry antibodies (see KRT) at room temperature for 1 h. Finally, after washing, the cells were analyzed by flow cytometer (Sony ID7000).

### 4.12 Confocal Microscopy

A lentiviral vector containing the green fluorescent protein (GFP)‐red fluorescent protein (RFP)‐LC3B plasmid was constructed by GeneChem (Shanghai, China). PSCs transfected with GFP‐RFP‐LC3B were cultured in 24‐well plates with cover slips and then treated with different reagents for 24 h. After fixation with formalin (10%), the cover slips were mounted over a microscope slide. Confocal microscopy (Zeiss LSM 780) was used to examine changes in LC3B localization and expression. Images were obtained using confocal microscopy and analyzed with Zeiss confocal software.

### 4.13 Primary PSCs Immunofluorescence

Primary PSCs were cultured on 24‐well slides for 24 h to allow for adhesion, followed by treatment under different conditions. The cells were then fixed in 4% paraformaldehyde at 4°C for 30 min, permeabilized with Triton X‐100, and washed with PBS. After blocking with 1% BSA solution for 60 min, the cells were incubated overnight at 4°C with α‐SMA antibody (1:200). Following washing with TBST, the cells were incubated with Cy3‐conjugated secondary antibody (dilution 1:1000) for 60 min. Nuclei were stained with DAPI. Finally, images were observed and captured using a fluorescence microscope (Olympus).

### 4.14 Reverse Transcription Quantitative Polymerase Chain Reaction (RT‐qPCR)

TRIzol Reagent (Invitrogen) was purposed to extract total RNA from PC cells and tissues. PrimeScript RT Master Mix (RR036A, Takara) was utilized for the RNA reverse‐transcribed into cDNA according to provided directions. Quantitative PCR (qPCR) was performed using TB Green Premix Ex Taq (RR420A, Takara) based on the manufacturer's recommendations. β‐actin acted as a control for cellular RNA. The primer sequences used in this research were placed in Table S1.

### 4.15 Plate Colony Formation

After digesting the KPC cells, they were mixed with DMEM complete medium (10% serum) and seeded at a density of 1×10^2^cells/2 mL in the lower chamber of a 6‐well transwell plate (0.4 µm pore size, Corning). Different treatment conditions of PSCs were added to the upper chamber at a density of 1×10^4^ cells/1 mL, after co‐culturing for one week, the upper chamber was removed, and the medium in the lower chamber was aspirated. The cells were gently washed twice with PBS. Finally, 2 mL of 4% paraformaldehyde was added to each well for fixation for 30 min. After washing once with PBS, 2 mL of crystal violet solution was added for staining for 10 min. The wells were then washed with PBS until no residue remained, and photographs were taken.

### 4.16 Transwell Assay

KPC cells (2×10^3^) suspended in 200 µL of serum‐free DMEM medium were added to the upper chamber of a transwell 6‐well plate (8 µm pore size; Corning). The lower chamber contained 600 µL of DMEM medium with 30% FBS and PSCs (2×10^4^) treated under different conditions. After incubating in the transwell system for 24 h, the KPC cells that had migrated to the bottom of the membrane were fixed and stained with crystal violet. The cells were then counted and analyzed under a microscope in five different fields of view.

### 4.17 Electron Microscopy

After washing the differently treated primary PSCs twice with PBS, they were fixed in 3% glutaraldehyde in 0.1 mol/L sodium cacodylate buffer (pH 7.2) at 4°C for 12 h, followed by fixation in 1% osmium tetroxide in 0.1 mol/L sodium cacodylate buffer at 4°C for 30 min. After washing with distilled water, the pellets or coverslips were dehydrated through a graded ethanol series, embedded, and polymerized in epoxy resin (TAAB Laboratories Equipment Ltd). Semi‐thin sections were stained with 1% toluidine blue for field selection. Ultra‐thin sections, approximately 60 nm thick, were cut and placed on 300‐mesh copper grids, then stained with uranyl acetate and lead citrate. The sections were observed using a Jeol 100 CX‐II transmission electron microscope at an accelerating voltage of 80 kV.

### 4.18 Immunohistochemistry and Immunofluorescence

PC specimens were dissected and fixed in 10% formalin, then embedded in paraffin and sectioned into 4 mm slices. These specimens were stained with Hematoxylin and Eosin (H&E) and Masson staining. For immunohistochemistry (IHC), tissue sections were placed at 58°C overnight, then washed in xylene, graded ethanol, and Tris‐buffered saline (TBS). Endogenous peroxidase activity was quenched with hydrogen peroxide, and antigen retrieval was performed using citrate buffer (pH 6.0). The sections were incubated overnight at 4°C with the primary antibody. After washing three times with TBS, the sections were incubated for 1 h with HRP‐conjugated secondary antibodies. Visualization was achieved using 3,3’‐diaminobenzidine (DAB), and nuclei were counterstained with hematoxylin. Finally, the sections were dehydrated, mounted with neutral balsam, and evaluated pathologically. For immunofluorescence (IF) staining of PC tissue, sections were fixed in methanol/acetone. The MCT1 antibody was diluted 1:250 in BSA, and anti‐collagen I was diluted 1:250 in BSA to label PSCs in PC tissue. For immunofluorescence staining of PSCs, cells were cultured in 24‐well plates for 24 h and fixed with 10% formalin. The cells were permeabilized with Triton X‐100 and blocked with 1% BSA solution for 60 min. They were then incubated overnight at 4°C with α‐SMA antibody (1:200). Cy3‐conjugated secondary antibody (1:1000) was used to recognize the α‐SMA antibody for 60 min. Nuclei were stained with DAPI, and images were captured using a fluorescence microscope (Olympus).

### 4.19 Co‐Immunoprecipitation (Co‐IP)

The Co‐IP assay for p‐ERK, PD‐1 and Vps34 was conducted via lysing PSCs with Pierce IP lysis buffer (#87787, Thermo Fisher Scientific) containing protease inhibitor cocktails and PMSF on ice for 30 min followed by sonication (power 10 for three cycles, 10s on and 50 s for rest) to release nuclear proteins completely. One‐tenth of the lysate was extracted as input. The remaining lysate was then incubated with anti‐p‐ERK, anti‐PD‐1, or anti‐Vps34 antibodies overnight at 4°C followed by the incubation of Protein A/G Magnetic Beads (Selleck China) at 4°C for 2 h. Then the protein/agarose complex was isolated and resuspended in loading buffer, degenerated, and further analyzed by Western blot analysis.

### 4.20 ELISA Detection

Extract 200 µL of cell supernatant from different sources and use various ELISA kits to detect different indicators. Detailed ELISA kit indicators are listed in Table S1, and the specific detection methods for ELISA are provided in the kit instructions.

### 4.21 Oil Red O Staining

Remove the supernatant from PSCs in 6‐well plates under different treatment conditions, wash with PBS, and fix with 4% paraformaldehyde for 10 min. Wash again with PBS and then 1 mL of prepared Oil Red O staining working solution and incubate for 10 min. Gently wash with PBS to remove excess stain. Finally, counterstain the nuclei with hematoxylin staining solution, wash, and then proceed with imaging and analysis.

### 4.22 Chromatin Immunoprecipitation (ChIP)

Chromatin immunoprecipitation was performed using the EZ‐ChIP (Sigma–Aldrich, 41105331) according to the manufacturer's instructions. Cells were seeded in 15 cm plates and subjected to cross‐linking with 1% formaldehyde for 20 min. Sonicate the collected cells to break most of the DNA into 200–1000 bp. 20 ul samples were taken as input for subsequent detection. The rest of the samples were incubated with anti‐STAT3 antibody or negative control antibody IgG at 4°C overnight. Protein G agarose beads were added to precipitate the protein‐DNA complex. After washing the complex, the protein was digested with protease K, and the remaining supernatant was purified. Enriched DNA fragments were amplified by PCR reaction and analyzed by 2% agarose gel electrophoresis. Primers for ChIP‐qPCR are listed in Table S1.

### 4.23 Luciferase Assay

Briefly, the sequence of the PD‐1 promoter (2 kb sequence upstream of the transcription initiation site) was constructed into pGL3‐based vectors and then transfected into cell lines. Luciferase activity was measured using a luciferase assay kit (Promega, USA, E1910). Firefly luciferase activity was normalized to the Renilla luciferase activity.

### 4.24 Single Cell RNA‐seq Cell Preparation

After tumor harvested, tissues were washed in ice‐cold RPMI1640 and dissociated using multi‐tissues dissociation kit 2 (Miltenyi) from Miltenyi Biotec as instructions. Cell count and viability was estimated using fluorescence Cell Analyzer (Countstar Rigel S2) with AO/PI reagent after removal erythrocytes (Miltenyi 130‐094‐183) and then debris and dead cells removal was decided to be performed or not (Miltenyi 130‐109‐398/130‐090‐101). Finally, fresh cells were washed twice in the RPMI1640 and then resuspended at 1×10^6^ cells per ml in 1×PBS and 0.04% bovine serum albumin.

### 4.25 Single Cell RNA‐seq Library Construction and Sequencing

Single‐cell libraries were constructed using the 10x Genomics Chromium platform or SeekOne DD kit. Cell viability and concentration were measured with an automated fluorescence counter, and samples were adjusted to 1000 cells/µL. Libraries were sequenced using paired‐end, 150 bp reads on an Illumina NovaSeq 6000 platform.

### 4.26 Processing the Single Cell RNA Sequencing Data

The raw sequencing data was processed by Fastp firstly to trim primer sequence and low‐quality bases. And then we used SeekOneTools to process the sequence data and aligned to human GRCh38 in order to obtain a gene expression matrix. We used Seurat (version 4.0.0) to filter low quality cells, cells with number of detected genes <200 or >5000 were omitted, and use Median absolute deviation (MAD) variance normal to remove cells affected by mitochondrial genes. And the remaining cells were used for the following analysis.

### 4.27 Canonical Correlation Analysis (CCA), Dimensionality Reduction, and Clustering

After quality control and filtering, library‐size normalization to each cell was performed by NormalizeData of Seurat (version 4.0.0). The variable genes were calculated by FindVariableGenes. Then, all libraries were combined together using FindIntegrationAnchors and IntegrateData with defaut parameters, and using ScaleData to regress out the variability of the numbers of Unique molecular identifiers (UMIs). Then the RunPCA and RunUMAP was used to reduce dimensions. FindClusters was used to cluster cells using the 20 dims at a resolution of 0.8.

### 4.28 Differentially Expressed Genes Analysis

The FindMarkers function was used to identify Differentially Expressed Genes (DEG) across the conditions, and the default Wilcoxon rank test was used. Genes were ranked by absolute log2FC, and those with *p*‐values > 0.05 (adjusted for multiple comparisons) and log2FC <0.25 were removed.

### 4.29 Enrichment Analysis

GO enrichment analysis of DEG was implemented by the clusterProfiler R package. GO terms with corrected *p*‐value less than 0.05 were considered significantly enriched by DEG. KEGG is a database resource for understanding high‐level functions and utilities of the biological system, such as the cell, the organism, and the ecosystem, from molecular‐level information, especially large‐scale molecular datasets generated by genome sequencing and other high‐through put experimental technologies (http://www.genome.jp/kegg/). We used clusterProfiler R package to test the statistical enrichment of DEG in KEGG pathways.

### 4.30 CellChat Analysis

CellPhoneDB Python package (version 2.1.7) was used to detect ligand‐receptor interactions and predict communications among different cell types. CellPhoneDB is a publicly available repository of curated receptors and ligands and their interactions. To narrow down the most relevant interactions, we looked for specific interactions classified by ligand/receptor expression in more than 10% of cells within a cell type. Pairwise comparisons were performed between the included cell types. We first randomly permuted the cluster labels of all cells 1000 times to determine the mean of the average receptor and ligand expression levels of the interacting clusters. This generated a null distribution for each receptor–ligand pair. By calculating the proportion of the means that were higher than the actual mean, a *p* value for the likelihood of the cell type specificity of the corresponding receptor–ligand complex was obtained. We then selected interactions that were biologically relevant.

### 4.31 Statistical Analysis

GraphPad Prism7 (GraphPad Software, San Diego, California, USA), R version 4.1.0, and Excel software were used for statistical analyses. The details were provided in the figure legends, *p* values < 0.05 were considered statistically significant.

All the antibodies, reagents, and compounds mentioned in this article can be found in the KRT of the supplementary materials.

## Author Contributions

**Yuhang Hu**: conceptualization, methodology, funding acquisition. **Wenfeng Zhuo**: conceptualization, methodology, data curation, writing – original draft, visualization, investigation, validation. **Rong Hu**: validation, formal analysis, visualization, methodology. **Shengbo Han**: methodology, validation, formal analysis. **Guozheng Lv**: formal analysis, data curation, visualization. **Yan Huang**: investigation, methodology, validation. **Yang Li**: investigation, validation, formal analysis. **Ping Hu**: methodology, data curation, investigation. **Yingsong Zhao**: investigation, visualization. **Zhu Zeng**: funding acquisition, investigation, methodology. **Hongda Wang**: data curation, formal analysis. **Eryang Zhao**: investigation, validation. **Guangyu Zhao**: validation, investigation. **Yong Zhao**: investigation, validation, formal analysis. **Gang Zhao**: funding acquisition, writing – review and editing, project administration, conceptualization.

## Funding

This study was supported by grants from the National Natural Science Foundation of China (82472710, 82273322, 82072755 to Gang Zhao, 82203532 to Zhu Zeng, 82303357 to Yuhang Hu).

## Ethics Statement

Approval of the research protocol by an institutional review board: The Ethics Committee of the Academic Medical Center of Huazhong University of Science and Technology approved the procedure of human specimen collection (Permission No: 2013, S199). All patients provided signed informed consent. The Ethics Committee of the Academic Medical Center of Huazhong University of Science and Technology (IACUC) approved the procedure of animal studies.

## Consent

All contributing authors agreed to the publication of this article.

## Conflicts of Interest

The authors declare no conflicts of interest.

## Supporting information




**Supporting File 1**: advs76008‐sup‐0001‐SuppMat.docx.

**Supporting File 2**: advs76008‐sup‐0002‐westernblotoriginaldata.pptx.

## Data Availability

All data needed to evaluate the conclusions in the article are present in the article and/or the Supplementary materials. The PC patients single‐cell expression matrix and cell cluster annotation information for dataset PRJCA001063 were downloaded from the NGDC database (https://ngdc.cncb.ac.cn/?lang=zh). Our mouse Single‐cell RNA‐seq data have been deposited at GEO (GSE293004). The scoring analysis of the lactate metabolic pathway (GOBP_LACTATE_METABOLIC_PROCESS) in PC and its relationship with immune cell infiltration were completed by GSCA (https://guolab.wchscu.cn/GSCA). Cell line marker gene selection was derived from CELL MAKER 2.0 (http://117.50.127.228/CellMarker/index.html). Genetic correlation analysis of pancreatic ductal adenocarcinoma patients and disease‐free survival and overall survival analysis derived from Gene Expression Profiling Interactive Analysis (GEPIA, http://gepia.cancer‐pku.cn/). The correlation analysis of specific genes with immune cell infiltration was derived from TIMER2.0 (http://timer.cistrome.org/). The transcription factor prediction of binding with PD‐1 promoter was perform on the JASPAR (https://jaspar.elixir.no/).
