# Supplementary material for: Targeting Lactate‐Driven Stromal Autophagy via MCT1 Disrupts the Immunosuppressive Niche and Sensitizes Pancreatic Cancer to PD‐1 Blockade
Source: Adv Sci (Weinh). 2026 Jun 9:e76008. Online ahead of print. doi: 10.1002/advs.76008 (PMC13336809; doi:10.1002/advs.76008)
Supplement: Supplementary file 1 — Supporting File 1: advs76008‐sup‐0001‐SuppMat.docx. [file ADVS-9999-e76008-s002.docx]

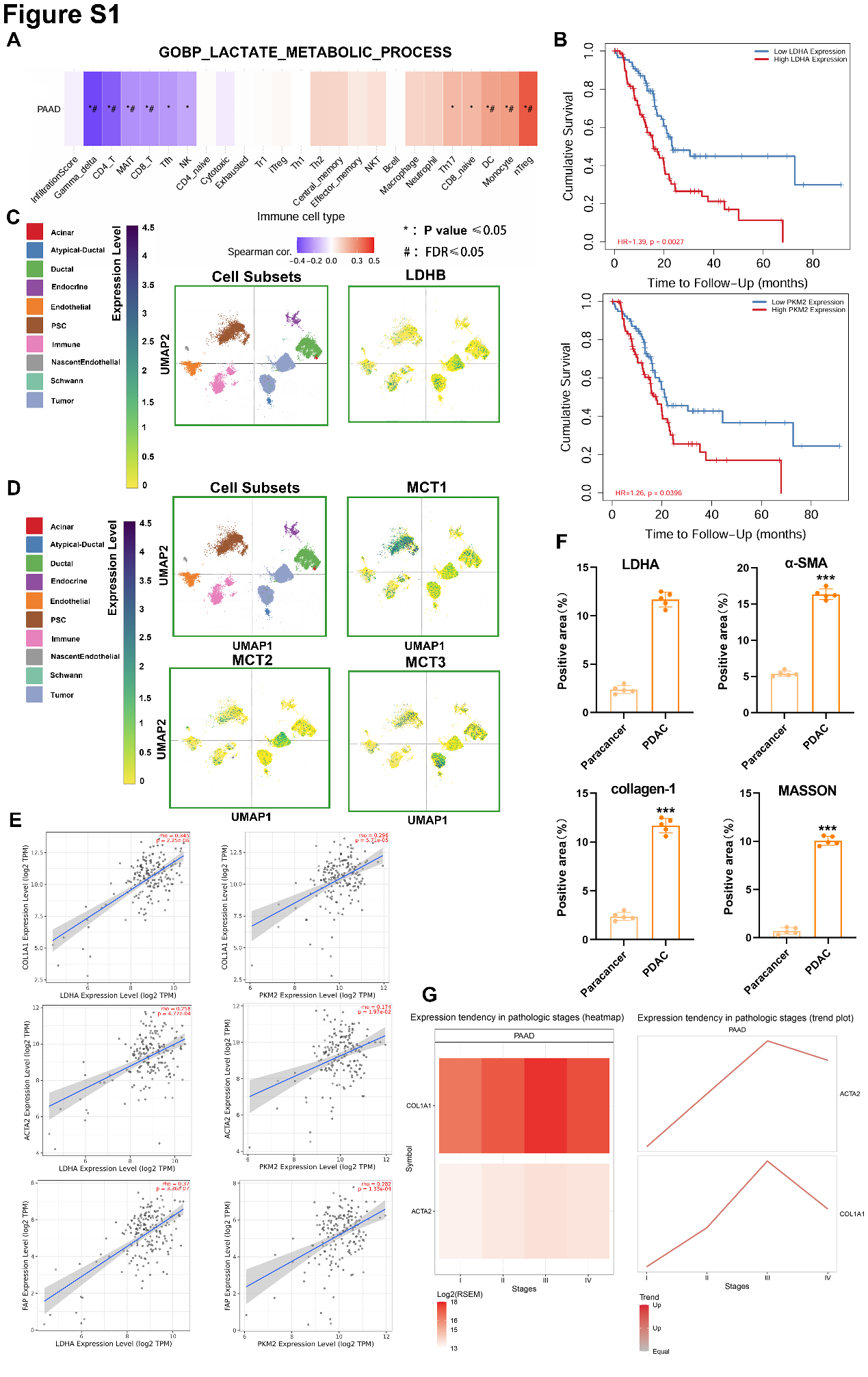


**Figures S1**

1. Correlation analysis of lactate metabolism dataset and immune cell infiltration in PC patients based on the TCGA database. (B) Prognostic analysis of high LDHA/PKM2 expression group and low LDHA/PKM2 expression group in PC patients based on the TCGA database. (C) Single-cell atlas of LDHB expression in human PC patients. (D) Single-cell atlas of MCT1, MCT2 and MCT3 expression in human PC patients. (E) Gene correlation analysis of LDHA/PKM2 with COL1A1/ACTA2/FAP in PC patients based on the TIMER2.0 database. (F) Statistical plot of positive regions for LDHA/Collagen1/a-SMA expression in immunohistochemistry and Masson’s staining (n=5). (G) Expression levels of COL1A1/ACAT2 in PC patients at different stages based on the TCGA database. Data are mean and ± SEM. *** P < 0.001. Statistical significance was determined by one-way ANOVA or two-sided Student's t-test as appropriate.


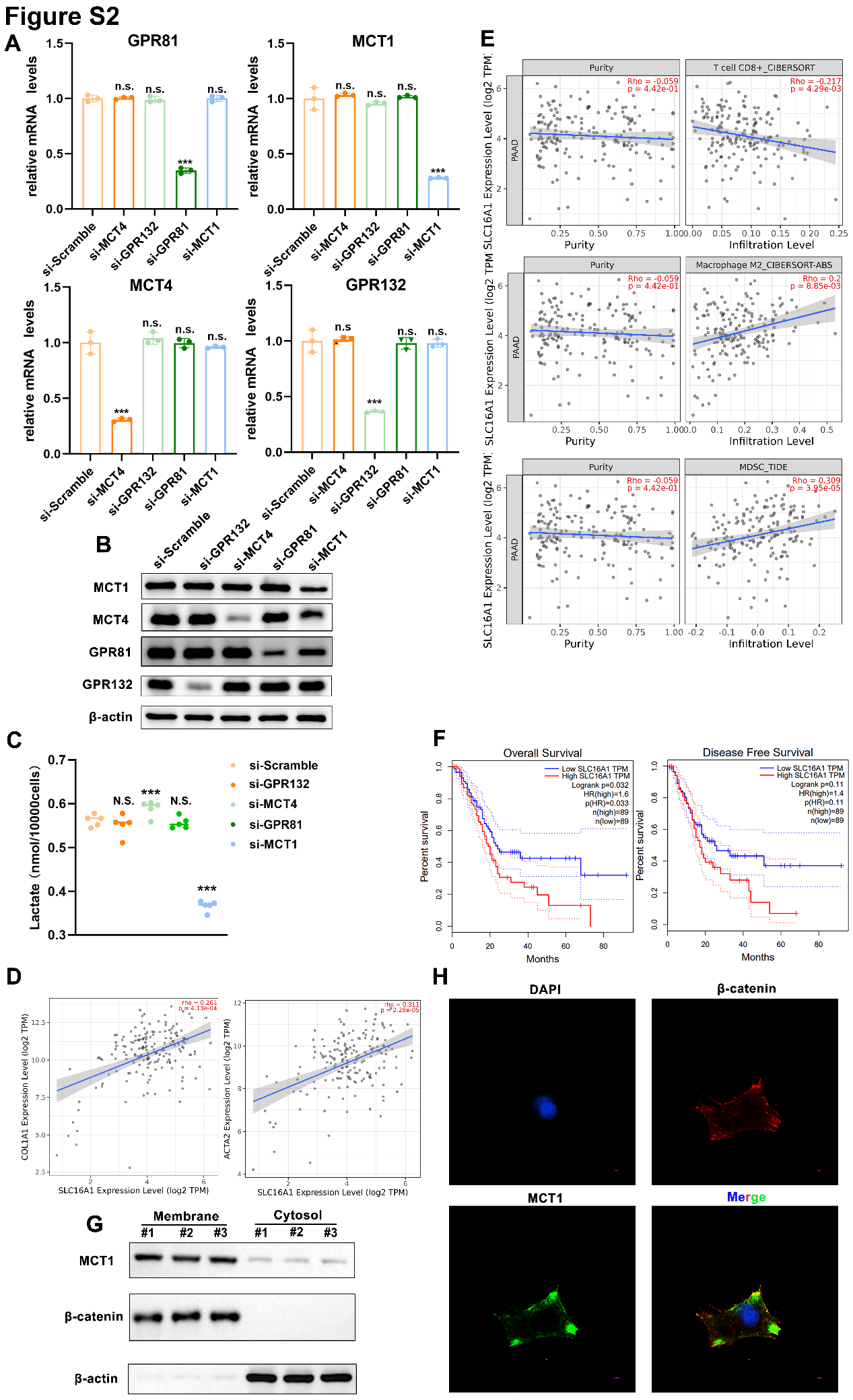


**Figure S2**

1. B) The mRNA and protein levels of MCT4, GPR132, GPR81, and MCT1 in PSCs (n=3) with si-Scramble, si-MCT4, si-GPR132, si-GPR81, or si-MCT1 transfected were measured by quantitative RT-PCR assays (A) and western blot (B). (C) Intracellular lactate concentration in PSCs (n=5). (D) Correlation analysis of MCT1 with immune cell infiltration in PDAC in TIMER2.0 database. (E) Gene correlation analysis of MCT1 and COL1A1/ACTA2 in PC patients by GEPIA. (F) Survival analysis of PDAC patients with high or low MCT1 expression by GEPIA. (G) The protein levels of MCT1 in PSCs (n=3) were measured by western blot. (H) Representative confocal microscopy images of the β-catenin (Red) and MCT1(Green) in PSCs (n=3). PSCs Nuclei was stained with DAPI. Scale bar: 5 μm. Data are mean and ± SEM. *** P < 0.001; n.s., not significant. Statistical significance was determined by one-way ANOVA or two-sided Student's t-test as appropriate.


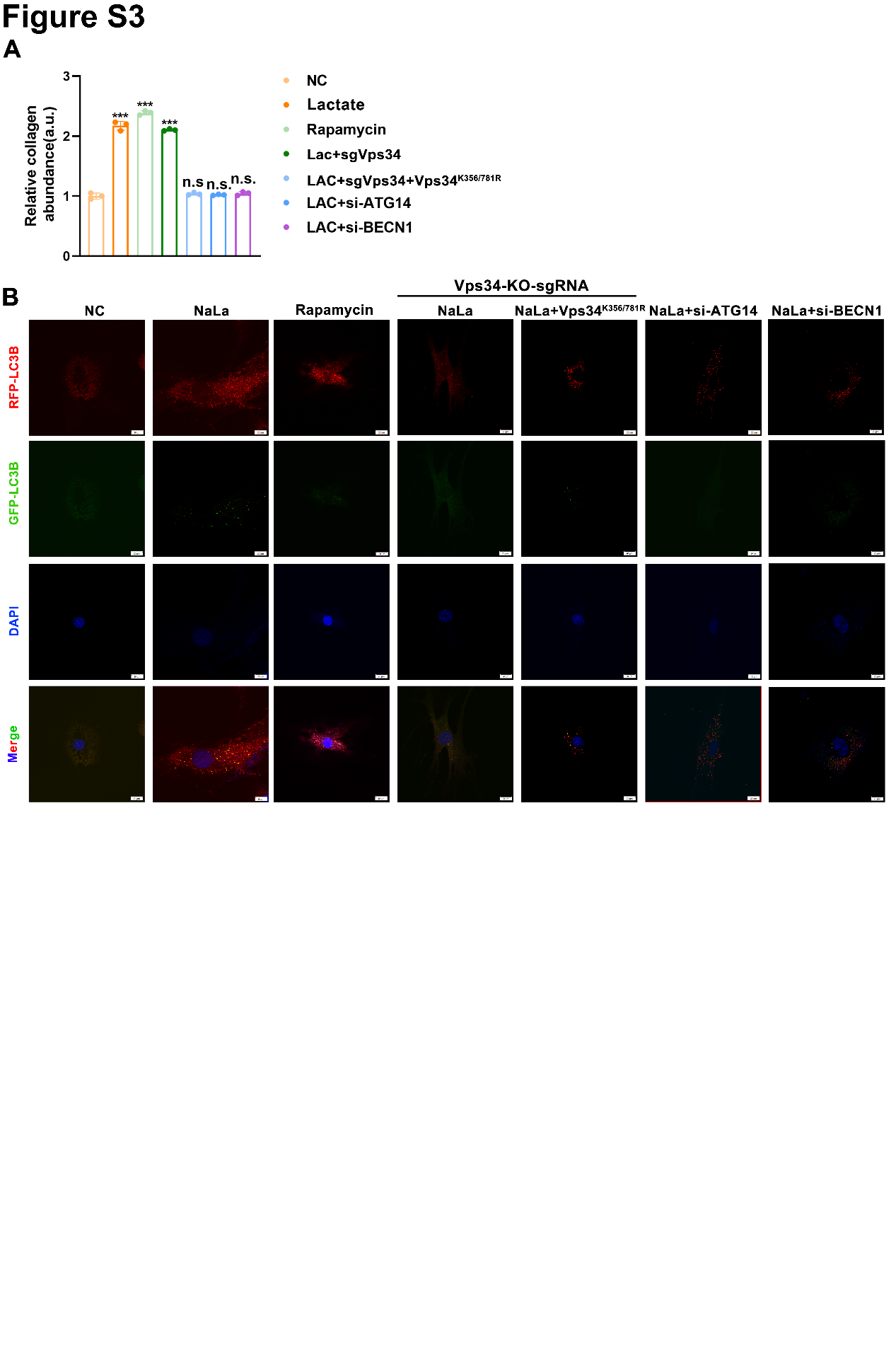


**Figure S3**

1. Collagen-1 in the supernatant of PSCs culture medium was detected using the ELISA kit (n=3). (B) LC3 double fluorescence in PSCs with or without with or without NaLa and rapamycin treated, si-Vps34, Vps34-K356/K781R, si-ATG14, si-BECN1 transfected were observed by confocal microscopy (n=3). Nuclei was stained with DAPI. Scale bar: 20 µm. Data are mean and ± SEM. *** P < 0.001; n.s., not significant. Statistical significance was determined by one-way ANOVA or two-sided Student's t-test as appropriate.


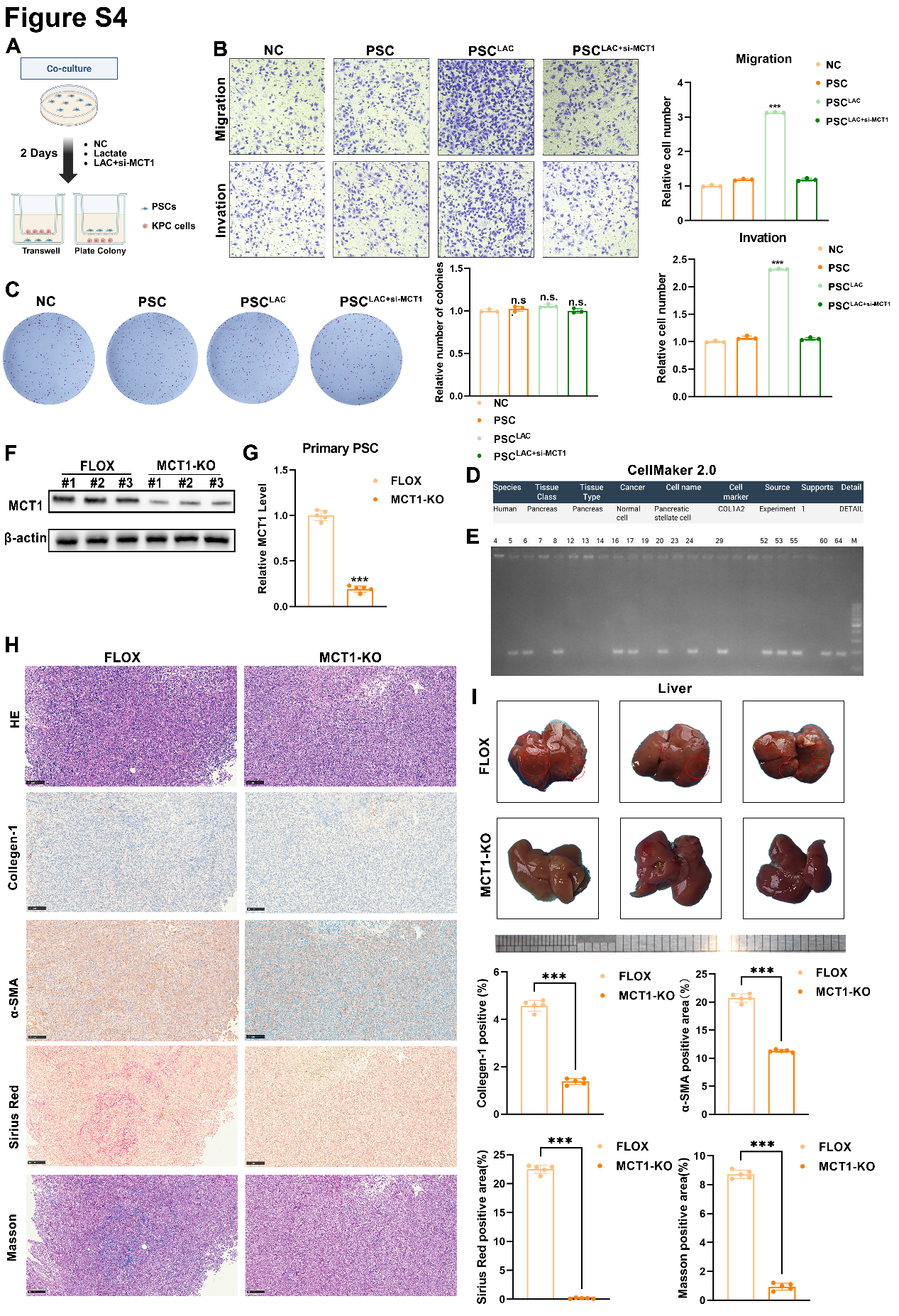


**Figure S4**

1. Schematic diagram of co-culture, after 2 days of different treatments (normal condition, 15 mM Lactate or 15 mM Lactate+si-MCT1), PSCs were moved to the bottom or upper of KPC cells for co-culture. (B) Transwell assays to assess the migration and invasion abilities of KPC cells (under normal culture, co-culture with PSC, co-culture with lactate-treated PSC, or co-culture with lactate-treated siMCT1-PSC), followed by statistical analysis (n=3). (C) Colony formation assays to assess the proliferation ability of above KPC cells, followed by statistical analysis (n=3). (D) COL1A2 as a Marker Gene for PSCs in the Cellmaker2.0 Database. (E) DNA gel electrophoresis for mouse genotyping. (F-G) The protein and mRNA levels of MCT1 in in primary PSCs from mice with or without PSCs-specific MCT1^-/-^ were measured by western blot analysis (n=3) (F) and quantitative RT-PCR assays(G) (n=5). (H) Immunohistochemical analysis of Collagen-1 and α-SMA levels in mice PC samples, along with Sirius Red staining, Masson’s trichrome staining, and H&E staining, followed by statistical analysis of positive area (n=5). Scale bars: 100 µm. (I) Representative images of liver metastasis in mice with KPC cells orthotopic injected in pancreas (n=3). Data are presented as the mean and standard deviation (error bars). *** P < 0.001; n.s., not significant.

**
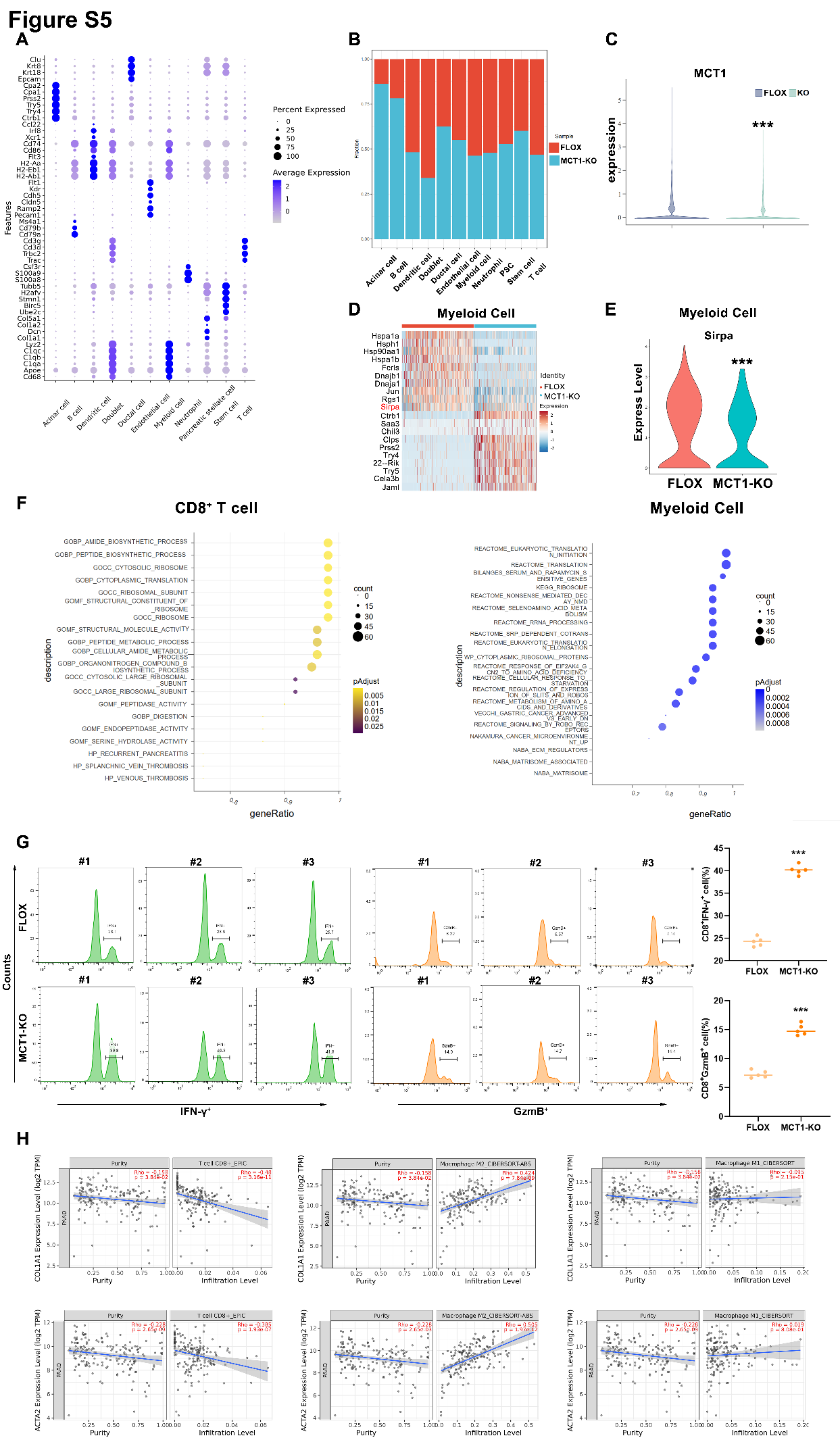
**

**Figure S5**

1. Cell marker genes used in single-cell analysis. (B) Single-cell analysis of MCT1 knockout levels in PSCs in the two samples. (C) Comparison of the proportions of various cell populations in above two samples. (D) Differential gene analysis in myeloid cells after standardization, displayed by using heatmap. (E) Relative Sirpa expression levels of myeloid cells in the two samples. (F) GO pathway enrichment analysis of CD8^+^ T cells and myeloid cells from single-cell analysis. (G) Flow cytometric analysis of IFN-γ^+^ and GzmB^+^ cells in CD8^+^ T cell populations sorted from in situ tumor samples with statistical analysis (n=5). (H) Correlation analysis of COL1A1 and ACTA2 genes with immune cell infiltration in PDAC patients by using TIMER2.0 database. Data are mean and ± SEM. *** P < 0.001. Statistical significance was determined by one-way ANOVA or two-sided Student's t-test as appropriate.


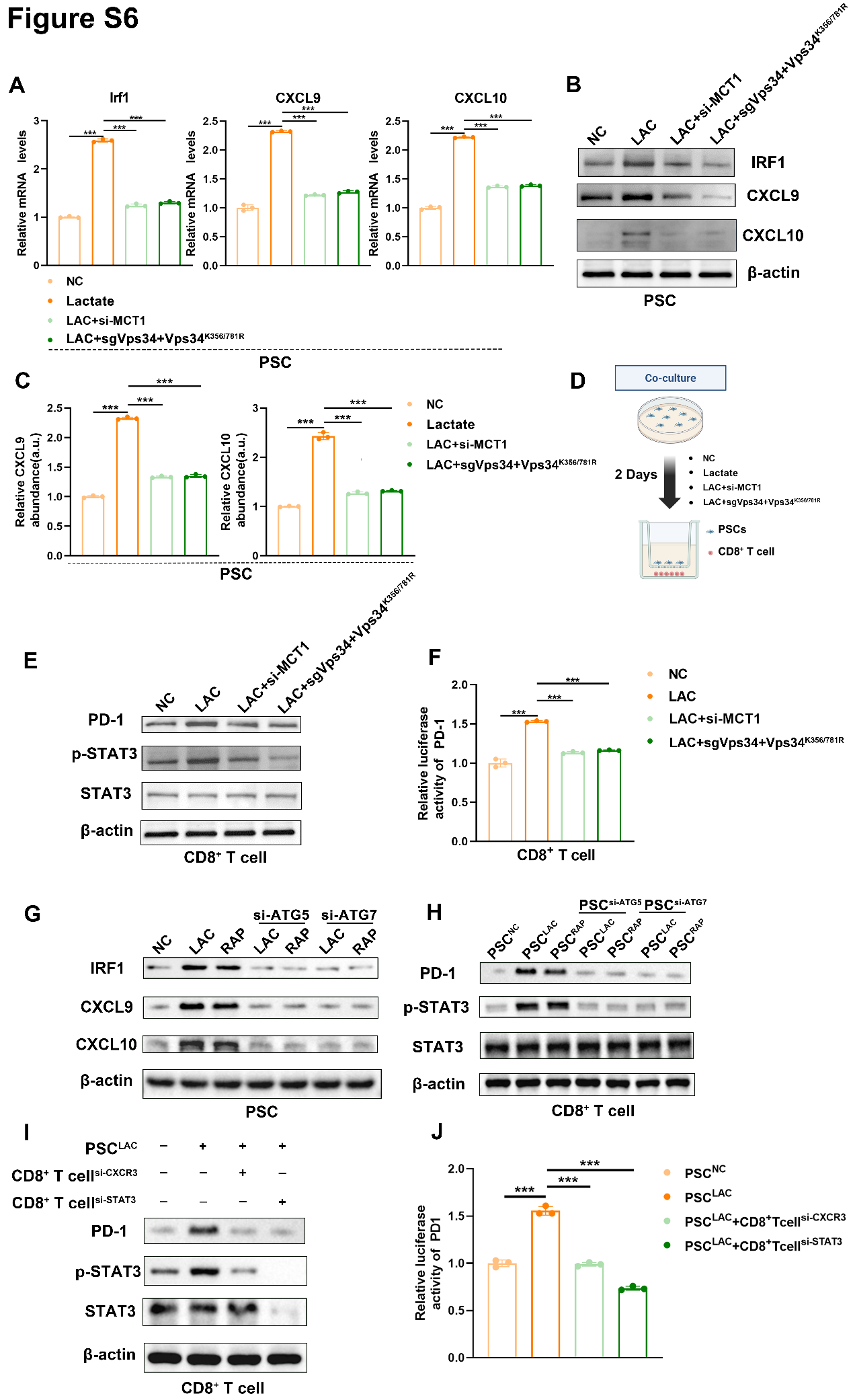


**Figure S6**

(A-B) The mRNA and protein levels of IRF1, CXCL9, and CXCL10 in primary mouse PSCs (treated with nothing, 15 mM Lactate, 15 mM Lactate+si-MCT1 or 15 mM Lactate + Vps34-knockout-sgRNA + Vps34-K^356/K781R^) were measured by quantitative RT-PCR assays (A) (n=3) and western blot analysis (n=3) (B). (C) CXCL9 and CXCL10 in the supernatant of the culture medium of primary mouse PSCs (treated with nothing, 15 mM Lactate, 15 mM Lactate+si-MCT1 or 15 mM Lactate + Vps34-knockout-sgRNA + Vps34-K^356/K781R^) were detected by using ELISA kits (n=3). (D) Schematic diagram of co-culture, after 2 days of different treatments (normal condition, 15 mM Lactate, 15 mM Lactate+si-MCT1 or 15 mM Lactate + Vps34-knockout-sgRNA + Vps34-K^356/K781R^), primary mouse PSCs were moved to the upper chamber of mouse CD8^+^ T cells for co-culture. (E) The protein levels of PD-1, p-STAT3 and STAT3 in above mouse CD8^+^ T cells were measured by western blot analysis (n=3). (F) Luciferase reporter assays show the relative activity of PD1 promoter in above mouse CD8^+^ T cells (n=3). Data are mean and ± SEM. *** P < 0.001. Statistical significance was determined by one-way ANOVA or two-sided Student's t-test as appropriate. (G) The protein levels of IRF1, CXCL9, and CXCL10 in primary mouse PSCs (treated with nothing, 15 mM Lactate, rapamycin, si-ATG5+LAC, si-ATG5+RAP, si-ATG7+LAC, si-ATG7+RAP) were measured by western blot analysis (n=3). (H) The protein levels of PD-1, p-STAT3 and STAT3 in mouse CD8^+^ T cells (co-cultured with PSCs in Figure 6G) were measured by western blot analysis (n=3). (I) After co-cultured with primary mouse PSCs (treated with 15mM lactate or not), the protein levels of PD-1, p-STAT3 and STAT3 in mouse CD8^+^ T cells (treated with si-CXCR3 or si-STAT3) were measured by western blot analysis (n=3). (J) Luciferase reporter assays show the relative activity of PD1 promoter in above mouse CD8^+^ T cells (n=3). Data are mean and ± SEM. *** P < 0.001. Statistical significance was determined by one-way ANOVA or two-sided Student's t-test as appropriate.

**
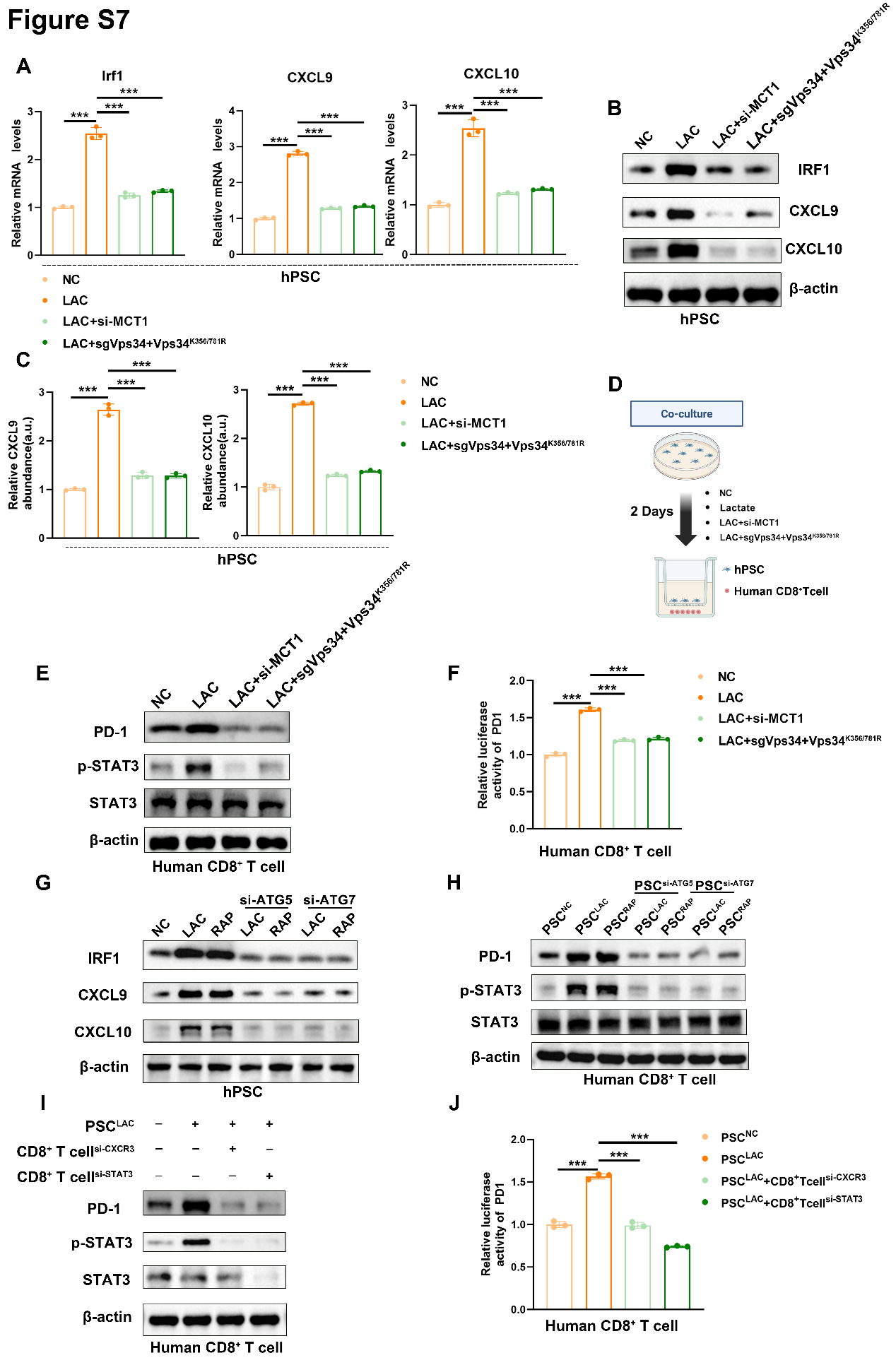
**

**Figure S7**

(A-B) The mRNA and protein levels of Irf1, CXCL9, and CXCL10 in human PSC (treated with nothing, 15 mM Lactate, 15 mM Lactate+si-MCT1 or 15 mM Lactate + Vps34-knockout-sgRNA + Vps34-K^356/K781R^) were measured by quantitative RT-PCR assays (A) (n=3) and western blot analysis (n=3) (B). (C) CXCL9 and CXCL10 in the supernatant of the culture medium of human PSCs (treated with nothing, 15 mM Lactate, 15 mM Lactate+si-MCT1 or 15 mM Lactate + Vps34-knockout-sgRNA + Vps34-K^356/K781R^) were detected by using ELISA kits (n=3). (D) Schematic diagram of co-culture, after 2 days of different treatments (normal condition, 15 mM Lactate, 15 mM Lactate+si-MCT1 or 15 mM Lactate + Vps34-knockout-sgRNA + Vps34-K^356/K781R^), human PSCs were moved to the upper chamber of human CD8^+^ T cells for co-culture. (E) The protein levels of PD-1, p-STAT3 and STAT3 in above human CD8^+^ T cells were measured by western blot analysis (n=3). (F) Luciferase reporter assays show the relative activity of PD1 promoter in above human CD8^+^ T cells (n=3). Data are mean and ± SEM. *** P < 0.001. Statistical significance was determined by one-way ANOVA or two-sided Student's t-test as appropriate. (G) The protein levels of IRF1, CXCL9, and CXCL10 in human PSCs (treated with nothing, 15 mM Lactate, rapamycin, si-ATG5+LAC, si-ATG5+RAP, si-ATG7+LAC, si-ATG7+RAP) were measured by western blot analysis (n=3). (H) The protein levels of PD-1, p-STAT3 and STAT3 in human CD8^+^ T cells (co-cultured with PSCs in Figure 6G) were measured by western blot analysis (n=3). (I) After co-cultured with human PSCs (treated with 15mM lactate or not), the protein levels of PD-1, p-STAT3 and STAT3 in human CD8^+^ T cells (treated with si-CXCR3 or si-STAT3) were measured by western blot analysis (n=3). (J) Luciferase reporter assays show the relative activity of PD1 promoter in above human CD8^+^ T cells (n=3). Data are mean and ± SEM. *** P < 0.001. Statistical significance was determined by one-way ANOVA or two-sided Student's t-test as appropriate.

**Table S1. Primers and siRNAs sequences of related genes appeared in the article**

**Table S2. Cell population and number statistics in single cell sequencing**

**Table S3. Differential expressed genes (DEG) of CD8^+^T cells in single cell sequencing**

**Table S4. DEG of myeloid cells in single cell sequencing**

**Table S5. Gene Ontology (GO) pathway enrichment analysis of DEG of CD8^+^T cells in single cell sequencing**

**Table S6. GO pathway enrichment analysis of DEG of myeloid cells in single cell sequencing**

**Table S7. Percentage of T cell subsets in the total population in single-cell sequencing**

**Table S8.** **RNA expression of various ligand receptor in different populations of cells**

**Table S9.** **Number of pairs with statistically different expression levels of ligand receptor in different populations of cells**

**Table S10. Representative cytokine differential genes of pancreatic stellate cell population in single cell sequencing**

**Table S1. Primers and siRNAs sequences of related genes appeared in the article**

| GENE Symbol | Forward Primer | Reverse Primer |
| --- | --- | --- |
| ACTA2 | CCCAGACATCAGGGAGTAATGG | TCTATCGGATACTTCAGCGTCA |
| COL1A1 | GCTCCTCTTAGGGGCCACT | ATTGGGGACCCTTAGGCCAT |
| FAP | GTCACCTGATCGGCAATTTGT | TCGTAGATGTAGTATGTCGCTGT |
| LDHA | CAAAGACTACTGTGTAACTGCGA | TGGACTGTACTTGACAATGTTGG |
| MAPK1 | GGTTGTTCCCAAATGCTGACT | CAACTTCAATCCTCTTGTGAGGG |
| PD-1 | CAGCTTGTCCAACTGGTCG | GCTCAAACCATTACAGAAGGCG |
| CXCR3 | GGTTAGTGAACGTCAAGTGCT | CCCCATAATCGTAGGGAGAGGT |
| CXCL10 | CCAAGTGCTGCCGTCATTTTC | GGCTCGCAGGGATGATTTCAA |
| CXCL9 | GGAGTTCGAGGAACCCTAGTG | GGGATTTGTAGTGGATCGTGC |
| IRF1 | ATGCCAATCACTCGAATGCG | CCTGCTTTGTATCGGCCTGT |
| MCT1 | GGTGGGCAGTGTTAGTCGG | GATAGGACCTCCAGCATACATGA |
| MCT4 | TGGGACGTTCCTTGTCTCTG | TGTAGAAAGGCCCAATCGTTTTT |
| GPR132 | TGCCTGTCTACAGTCAACAGT | TCTGGACACTTCTTGCCGAGA |
| GPR81 | GCTTACCCCTTCGGACAGAC | ATGCTCCCGGCCCTATTCA |
|  |  |  |
| siRNAs Name | Sense | Antisense |
| si-MCT1 (Mouse) | UCUAGUAUCGUUAUGUGUGUC | CACACAUAACGAUACUAGAUU |
| si-MCT1 (Human) | UCCAAAUAUCGUUAUAUGCGC | GCAUAUAACGAUAUUUGGAUU |
| si-GPR81 | AUGACUUUUCUUUUUCUGGUU | CCAGAAAAAGAAAAGUCAUCU |
| si-GPR132 | UGUAGAUGAUCCACAAUGGCA | CCAUUGUGGAUCAUCUACAUC |
| si-MCT4 | ACGUUACUCUCUUUCUAAGGC | CUUAGAAAGAGAGUAACGUUU |
| si-ATG14 | UCGAAAUAGACGAAAUCACCG | GUGAUUUCGUCUAUUUCGACG |
| si-BECN1 | AAGCUAUUAGCACUUUCUGUA | CAGAAAGUGCUAAUAGCUUCA |
| si-CXCR3 (Mouse) | UCUCGUUUUCCCCAUAAUCGU | GAUUAUGGGGAAAACGAGAGC |
| si-CXCR3 (Human) | AGAAGUUGAUGUUGAAGAGGG | CUCUUCAACAUCAACUUCUAC |
| si-STAT1 (Mouse) | UUGAGAUUCCAAAUGCUUCCG | GAAGCAUUUGGAAUCUCAAGG |
| si-STAT1 (Human) | AGCAAAUGAAACUUUUCUGCG | CAGAAAAGUUUCAUUUGCUGU |
| si-ATG5 (Mouse) | UCUUUGUCAUCUGUCAUUCUU | GAAUGACAGAUGACAAAGAUG |
| si-ATG5 (Human) | AUUCUUAUUUCAACCAAAGCC | CUUUGGUUGAAAUAAGAAUUU |
| si-ATG7 (Mouse) | AGUUUCUUGCCAGAUUGUCAC | GACAAUCUGGCAAGAAACUUG |
| si-ATG7 (Human) | AAGAUACAGGAAUUGGAUCCG | GAUCCAAUUCCUGUAUCUUCA |
| CHIP | Forward Primer | Reverse Primer |
| PD-1 | TATGGTTGTGAGGTGCCG | TTCCAGAGGACCCAGATT |

**Table S2. Cell population and number statistics in single cell sequencing**

| Cell Cluster | FLOX | MCT1-KO |
| --- | --- | --- |
| Acinar cell | 23 | 145 |
| B cell | 67 | 240 |
| Dendritic cell | 116 | 108 |
| Doublet | 37 | 19 |
| Ductal cell | 1020 | 1825 |
| Endothelial cell | 106 | 130 |
| Myeloid cell | 2543 | 2195 |

**Table S7. Percentage of T cell subsets in the total population in single-cell sequencing**

| Cell Type | FLOX | MCT1-KO |
| --- | --- | --- |
| Pdcd1+ Cd8+ Exhausted T cell | 12.83% | 1.05% |
| Gpr183+ Cd8+ memory T cell | 31.60% | 11.58% |
| Nkg7+ Cd8+ Cytokines effector T cell | 23.98% | 22.74% |
| Proliferating T cell | 13.57% | 7.16% |
| Tnfrsf4+ Cd4+ memory T cell | 8.18% | 35.58% |
| NK cell | 4.83% | 8.63% |
| Doublet | 0.74% | 8.00% |
| Ccr7+ Cd8+ naive T cell | 4.28% | 5.26% |

**Table S10. Representative cytokine differential genes of pancreatic stellate cell population in single cell sequencing**

| GENE Symbol | FLOX | MCT1-KO |
| --- | --- | --- |
| Irf1 | 0.2844 | -0.2542 |
| Cxcl9 | 0.1898 | -0.1697 |
| Cxcl10 | 0.1724 | -0.1541 |
| Stat1 | 0.2692 | -0.2406 |
| Cxcl5 | -0.1676 | 0.1498 |
| Cxcl1 | 0.0198 | -0.0177 |
| Cxcl2 | 0.13 | -0.1162 |
| Cxcl3 | 0.0798 | -0.0713 |
| Cxcl16 | 0.1507 | -0.1347 |
| Il1b | 0.1147 | -0.1025 |
| Il18bp | 0.1808 | -0.1617 |
| Il4ra | 0.1053 | -0.0941 |
| Il33 | 0.0971 | -0.0868 |
| Ccl8 | 0.1919 | -0.1715 |
| Ccl4 | 0.1504 | -0.1344 |
| Ccl3 | 0.1279 | -0.1143 |
| Ccl12 | 0.1213 | -0.1104 |
| Ccl5 | 0.0973 | -0.0881 |
| Tnfaip2 | -0.0747 | 0.0668 |
| Tnfaip8 | 0.1292 | -0.1155 |
| Ifnar2 | 0.0834 | -0.0745 |
| Csf1 | 0.1147 | -0.1025 |
| Csf1r | 0.0831 | -0.0743 |

**KEY RESOURCES TABLE**

| **REAGENT OR RESOURCE** | **SOURCE** | **IDENTIFIER** |
| --- | --- | --- |
| **Antibodies** | | |
| Anti-a-SMA | Proteintech (China) | 80008-1-RR |
| Anti-Collagen-1 | Proteintech (China) | 14695-1-AP |
| Anti-FAP | abcam | ab314456 |
| Anti-β-actin | Proteintech (China) | 66009-1-Ig |
| Anti-MCT-4 | abcam | ab308528 |
| Anti-MCT-1 | Proteintech (China) | 20139-1-AP |
| Anti-GPR132 | ThermoFisher | PA5-102062 |
| Anti-GPR81 | ThermoFisher | PA5-114741 |
| Anti-LDHA | Proteintech (China) | 19987-1-AP |
| Anti-LC3B | abcam | ab192890 |
| Anti-BECN1 | abcam | ab207612 |
| Anti-P62 | abcam | ab109012 |
| Anti-L-Lactyl Lysine | PTM-biolab | PTM-1401RM |
| Anti-VPS34 | abcam | ab124905 |
| Anti-ATG14 | Proteintech (China) | 28021-1-AP |
| Cy3 conjugated Goat Anti-Rabbit IgG (H+L) | Servicebio (China) | GB21303 |
| FITC conjugated Goat Anti-Mouse IgG (H+L) | Servicebio (China) | GB22301 |
| APC Anti-Mouse CD8a | Proteintech (China) | APC-65069 |
| FITC Plus Anti-Mouse CD3 | Proteintech (China) | FITC-65060 |
| FITC Plus Anti-Mouse PD-1 | Proteintech (China) | FITC-65142 |
| Anti-CXCL9 | abcam | ab320827 |
| Anti-CXCL10 | abcam | ab306587 |
| Anti-IRF1 | abcam | ab232861 |
| Anti-PD-1 | abcam | ab300425 |
| Anti-STAT3 | abcam | Ab68153 |
| Anti-STAT3 (phospho Y705) | abcam | Ab267373 |
| Anti-CXCR3 | abcam | ab288437 |
| Anti-mouse horseradish peroxidase (HRP)-conjugated secondary antibodies | CST | 7076S |
| Anti-rabbit horseradish peroxidase (HRP)-conjugated secondary antibodies | CST | 7074S |
| **Chemicals, peptides and reagent consumables** | | |
| DAPI(Solution) | Servicebio (China) | G1012-10ML |
| DMEM Medium | GIBCO | C11995500BT |
| Lipofectamine 2000 | ThermoFisher | [11668030](https://www.thermofisher.cn/order/catalog/product/cn/en/11668030) |
| Lactate | MedChemExpress | [HY-B2227](https://www.medchemexpress.cn/lactate.html) |
| TGF-B1 | MedChemExpress | [HY-P70648](https://www.medchemexpress.cn/recombinant-proteins/tgf-beta-1-tgfb1-protein-mouse-rat-hek293.html) |
| chloroquine | MedChemExpress | HY-17589A |
| Mouse Collagen Type I ELISA Kit | abcam | ab285314 |
| Oil red O staining kit | Beyotime (China) | C0157S |
| Hematoxylin | MedChemExpress | HY-N0116 |
| Fetal Bovine Serum, Premium Plus | GIBCO | A5669701 |
| AZD3965 | MedChemExpress | HY-12750 |
| stubRFP-sensGFP-LC3 Lentivirus | Genechem (China) | GE166.2 |
| Rapamycin | MedChemExpress | AY-22989 |
| Mouse CXCL9 ELISA Kit | abcam | ab137792 |
| Mouse CXCL10 ELISA Kit | abcam | ab260067 |
| Cycloheximide | MedChemExpress | HY-12320 |
| Luciferase assay kit | Promega (USA) | E1910 |
| Anti-Mouse PD-1 | MedChemExpress | HY-P99144 |
| Tamoxifen | MedChemExpress | HY-13757A |
| Corn oil | MedChemExpress | HY-Y1888 |
| Lactic Acid assay kit | Nanjing Jiancheng Bioengineering Institute (China) | A019-2-2 |
| Triglyceride assay kit | Nanjing Jiancheng Bioengineering Institute (China) | A110-1-1 |
| TRIzol | Invitrogen | 15596026CN |
| EZ-ChIP | Sigma-Aldrich | 41105331 |
| PrimeScript RT Master Mix 41105331 | Takara | RR036A |
| TB Green® Premix Ex Taq™ | Takara | RR420A |
| Cocktail | MedChemExpress | HY-K0021 |
| RIPA Lysis Buffer (Strong) | Servicebio (China) | G2002-30ML |
| PMSF | Servicebio (China) | G2008-1ML |
| SDS-PAGE gel | Servicebio (China) | G2037-50T |
| PVDF | Millipore | IPFL00010 |
| ECL | Pierce | 34095 |
| Protein A/G Magnetic Beads | Selleck（China） | B23201 |
| Pierce IP lysis buffer | ThermoFisher | #87787 |
| paraformaldehyde | Beyotime (China) | P0099-100ml |
| crystal violet solution | Beyotime (China) | C0121-100ml |
| Type VI collagenase | MedChemExpress | HY-E70005I |
| Type I collagenase | MedChemExpress | HY-O0004 |
| Recombinant DNase I | MedChemExpress | HY-108882A |
| 0.25% Trypsin-EDTA (1x), phenol red | MedChemExpress | HY-K3007 |
| 70µm cell mesh | Corning | 431751 |
| DMEM/F-12 | GIBCO | 11320033 |
| transwell 6-well plate（0.4um） | Corning | 3412 |
| transwell 6-well plate（8um） | Corning | 3428 |
