# Supplementary material for: Targeting Lactate‐Driven Stromal Autophagy via MCT1 Disrupts the Immunosuppressive Niche and Sensitizes Pancreatic Cancer to PD‐1 Blockade
Source: Adv Sci (Weinh). 2026 Jun 9:e76008. Online ahead of print. doi: 10.1002/advs.76008 (PMC13336809; doi:10.1002/advs.76008)
Supplement: Supplementary file 2 — Supporting File 2: advs76008‐sup‐0002‐westernblotoriginaldata.pptx. [file ADVS-9999-e76008-s001.pptx]

## Slide 1
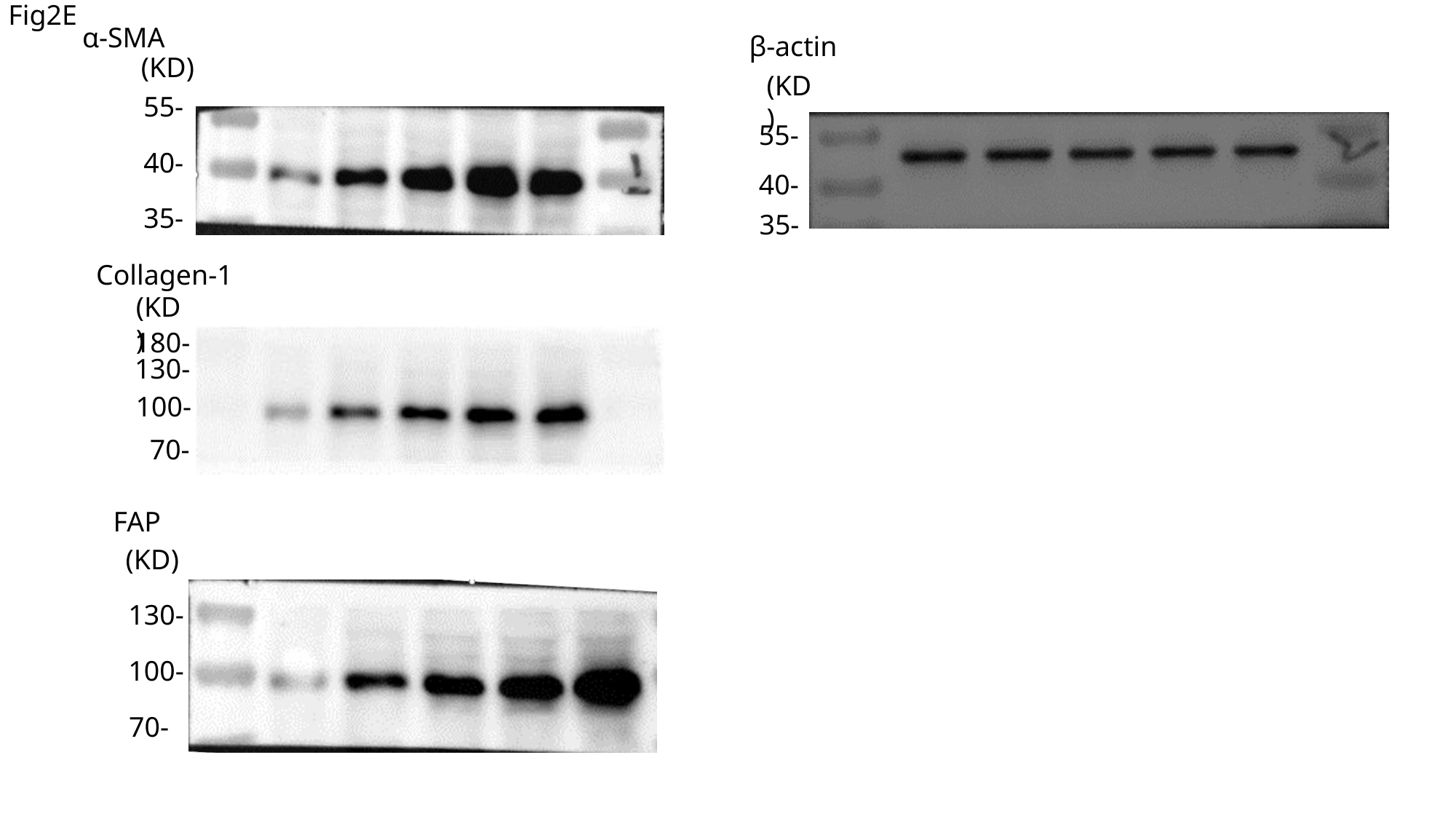

Fig2E
α-SMA
β-actin
(KD)
(KD)
55-
55-
40-
40-
35-
35-
Collagen-1
(KD)
180-
130-
100-
70-
FAP
(KD)
130-
100-
70-

## Slide 2
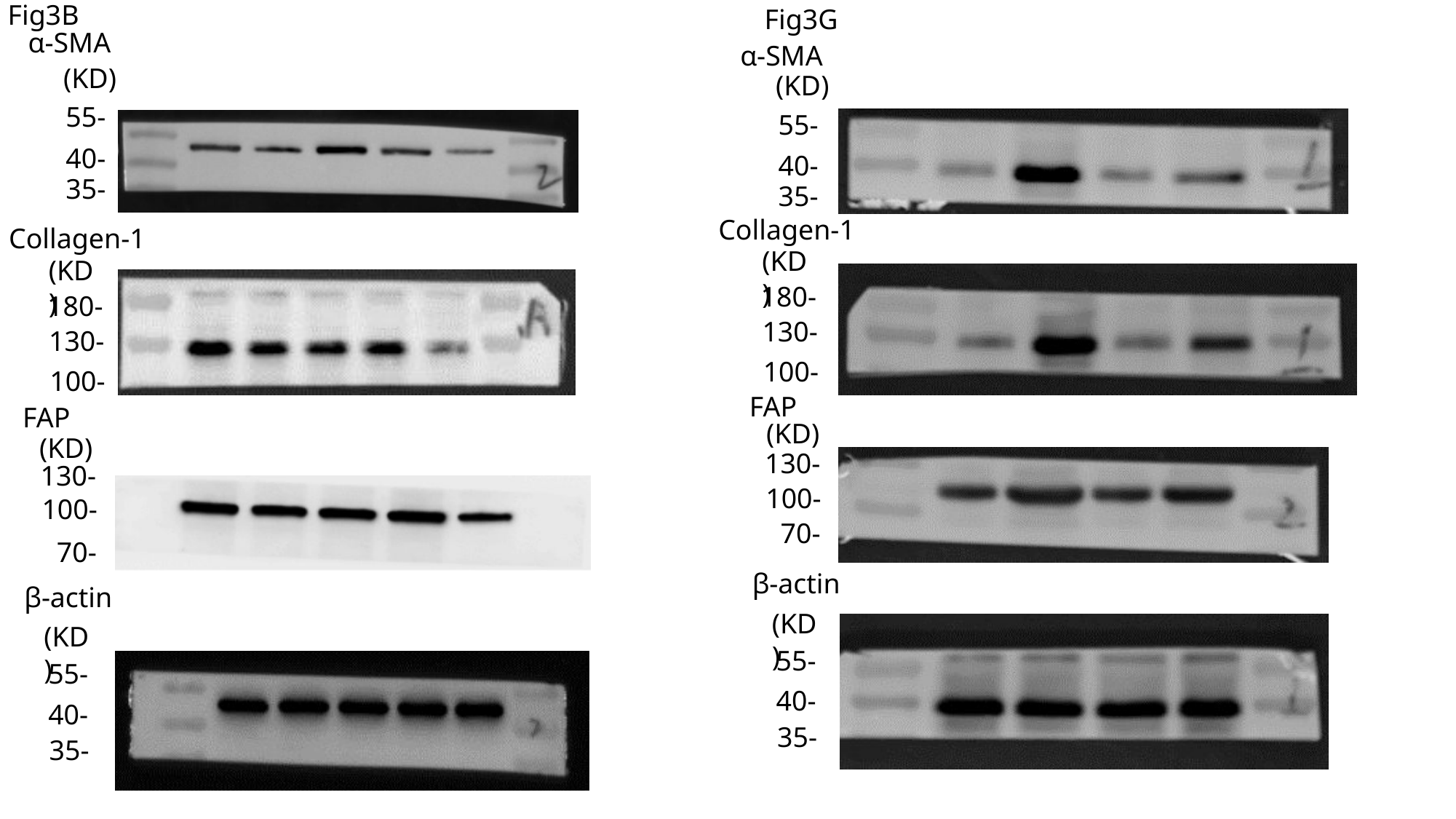

Fig3B
Fig3G
α-SMA
α-SMA
(KD)
(KD)
55-
55-
40-
40-
35-
35-
Collagen-1
Collagen-1
(KD)
(KD)
180-
180-
130-
130-
100-
100-
FAP
FAP
(KD)
(KD)
130-
130-
100-
100-
70-
70-
β-actin
β-actin
(KD)
(KD)
55-
55-
40-
40-
35-
35-

## Slide 3
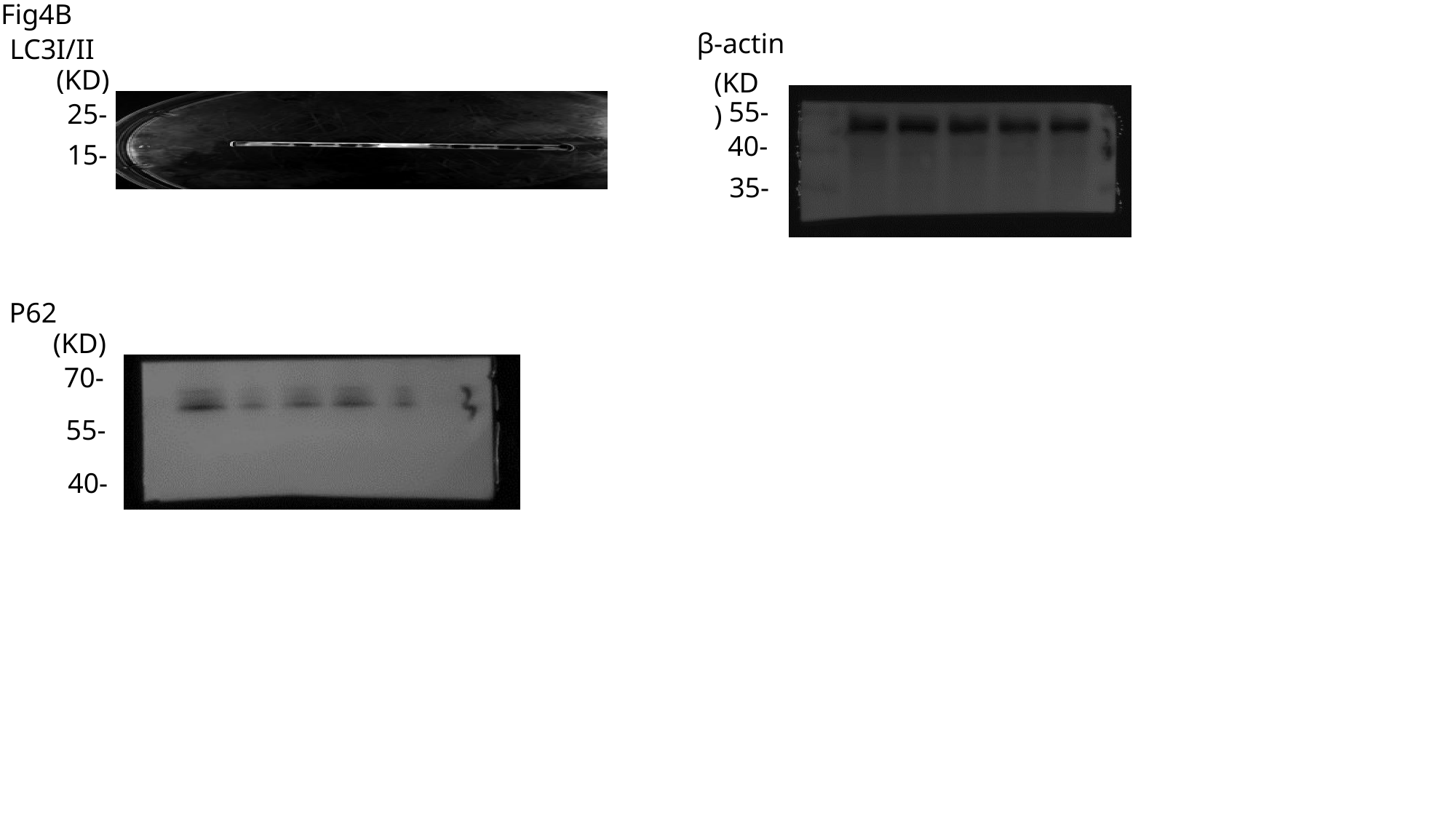

Fig4B
β-actin
LC3I/II
(KD)
(KD)
55-
25-
40-
15-
35-
P62
(KD)
70-
55-
40-

## Slide 4
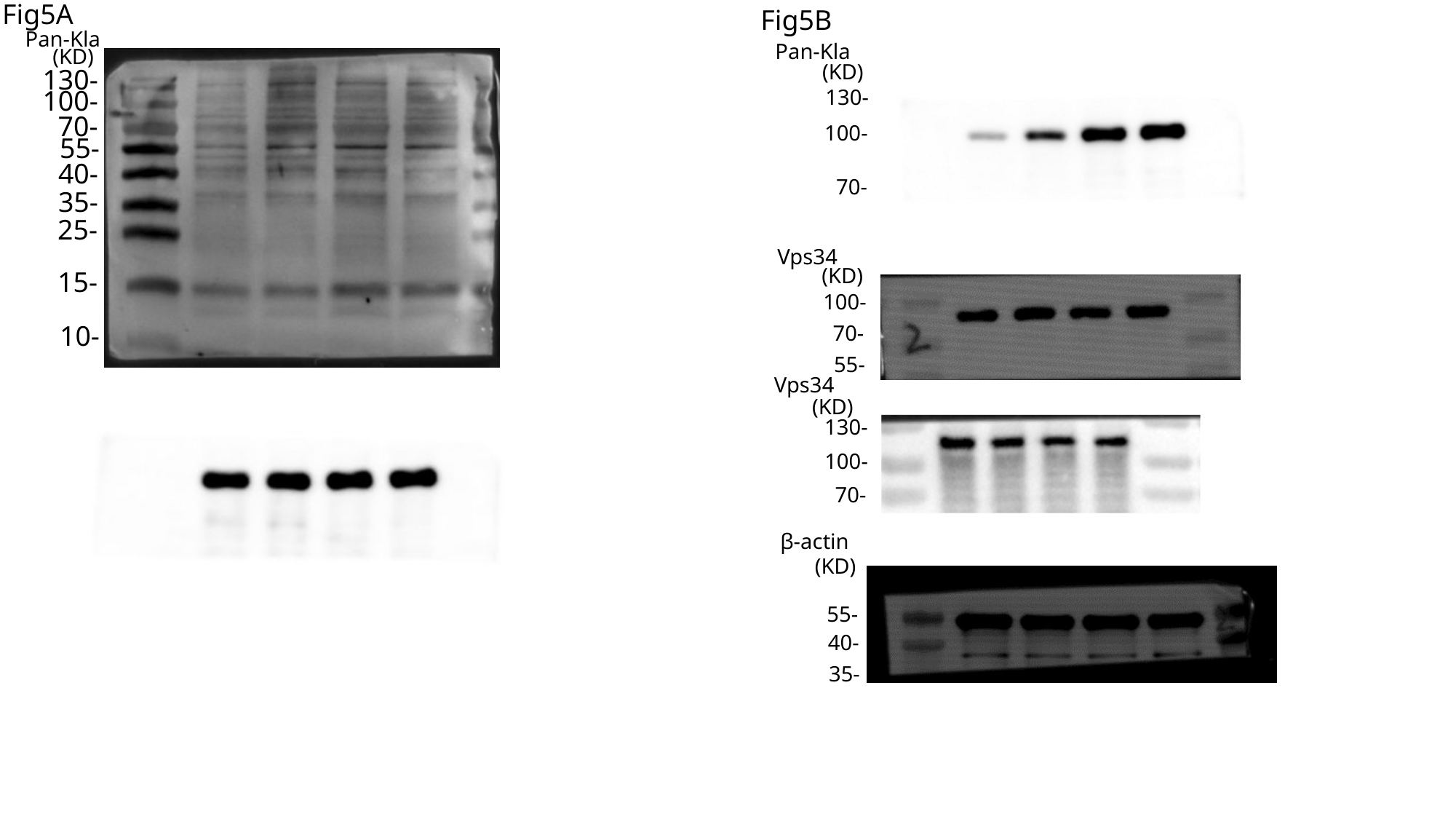

Fig5A
Fig5B
Pan-Kla
Pan-Kla
(KD)
(KD)
130-
100-
130-
70-
100-
55-
40-
70-
35-
25-
Vps34
(KD)
15-
100-
10-
70-
55-
Vps34
(KD)
130-
100-
70-
β-actin
(KD)
55-
40-
35-

## Slide 5
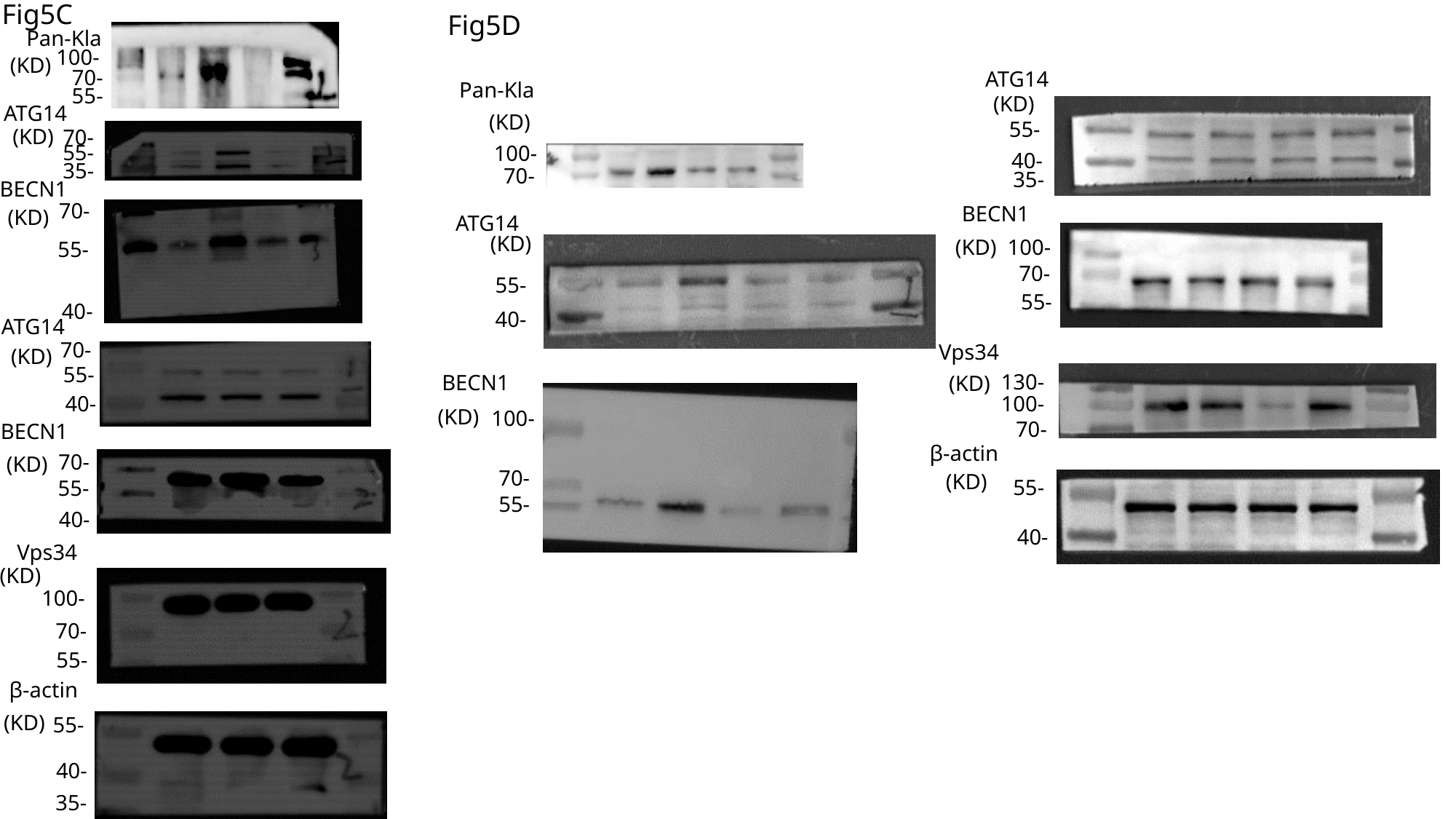

Fig5C
Fig5D
Pan-Kla
100-
(KD)
70-
ATG14
Pan-Kla
55-
(KD)
ATG14
(KD)
55-
(KD)
70-
55-
100-
40-
35-
70-
35-
BECN1
70-
BECN1
(KD)
ATG14
(KD)
(KD)
100-
55-
70-
55-
55-
40-
40-
ATG14
70-
Vps34
(KD)
55-
130-
BECN1
(KD)
40-
100-
(KD)
100-
70-
BECN1
β-actin
70-
(KD)
70-
(KD)
55-
55-
55-
40-
40-
Vps34
(KD)
100-
70-
55-
β-actin
(KD)
55-
40-
35-

## Slide 6
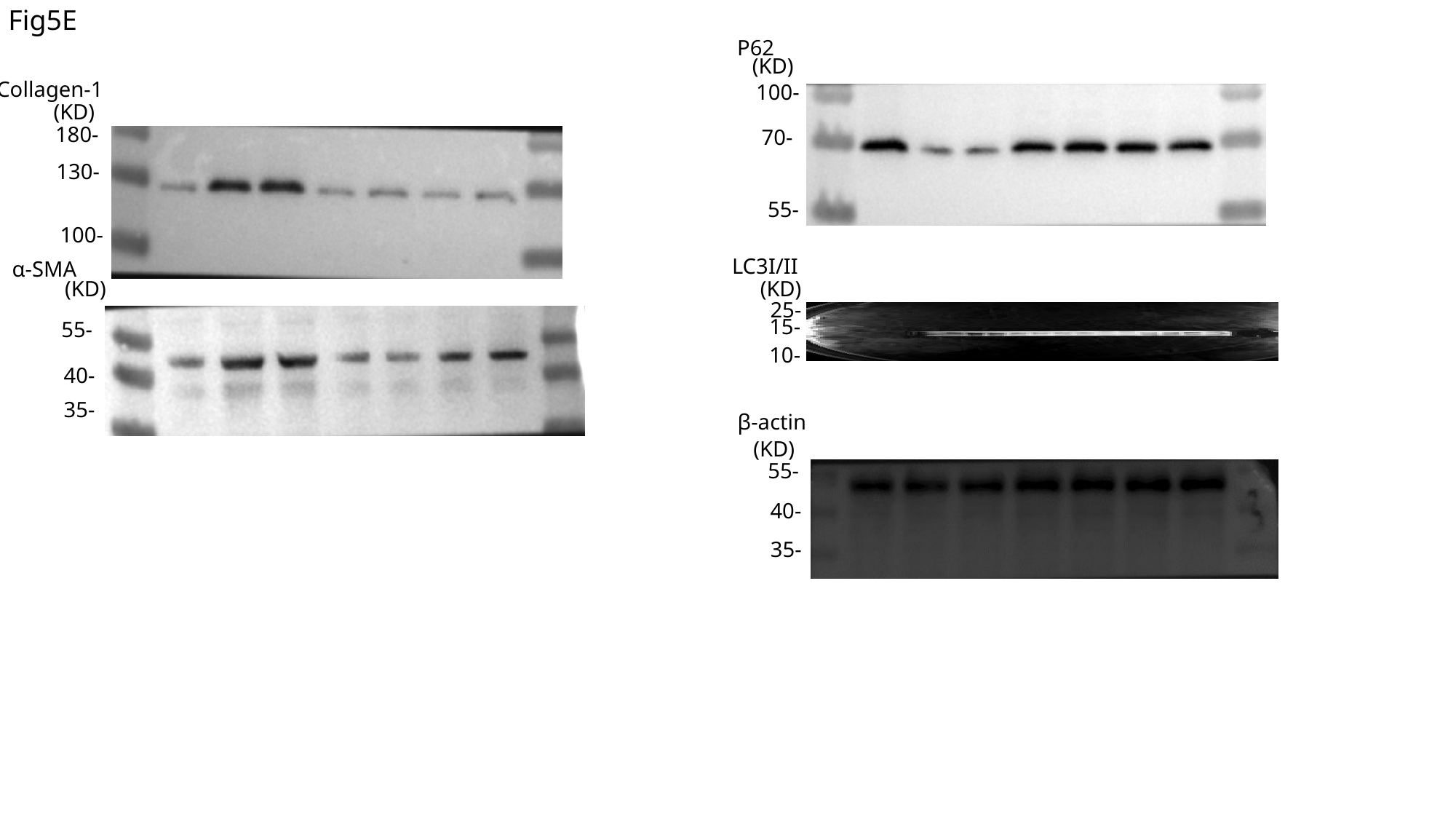

Fig5E
P62
(KD)
Collagen-1
100-
(KD)
180-
70-
130-
55-
100-
LC3I/II
α-SMA
(KD)
(KD)
25-
15-
55-
10-
40-
35-
β-actin
(KD)
55-
40-
35-

## Slide 7
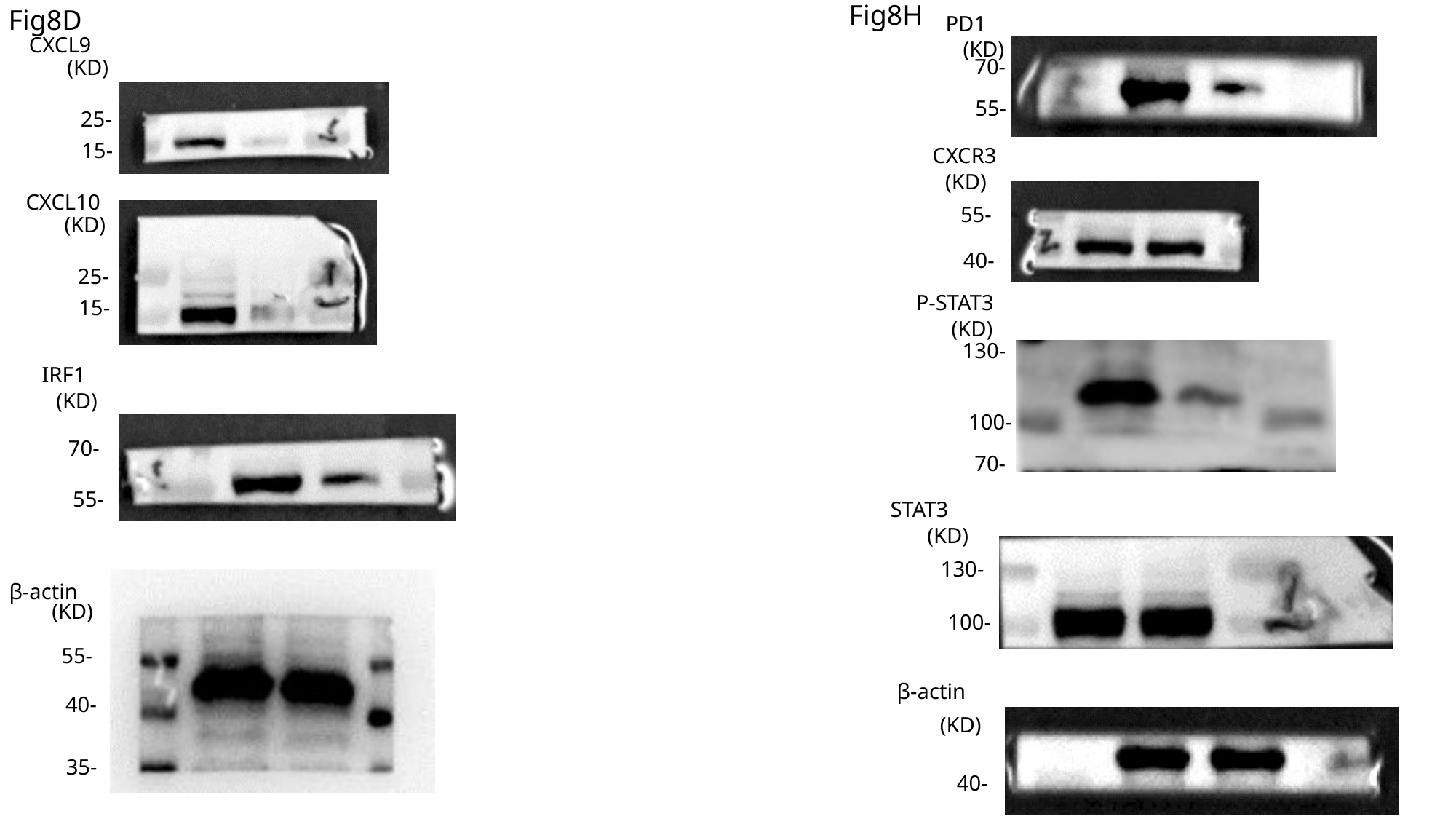

Fig8H
Fig8D
PD1
CXCL9
(KD)
70-
(KD)
55-
25-
15-
CXCR3
(KD)
CXCL10
55-
(KD)
40-
25-
P-STAT3
15-
(KD)
130-
IRF1
(KD)
100-
70-
70-
55-
STAT3
(KD)
130-
β-actin
(KD)
100-
55-
β-actin
40-
(KD)
35-
40-

## Slide 8
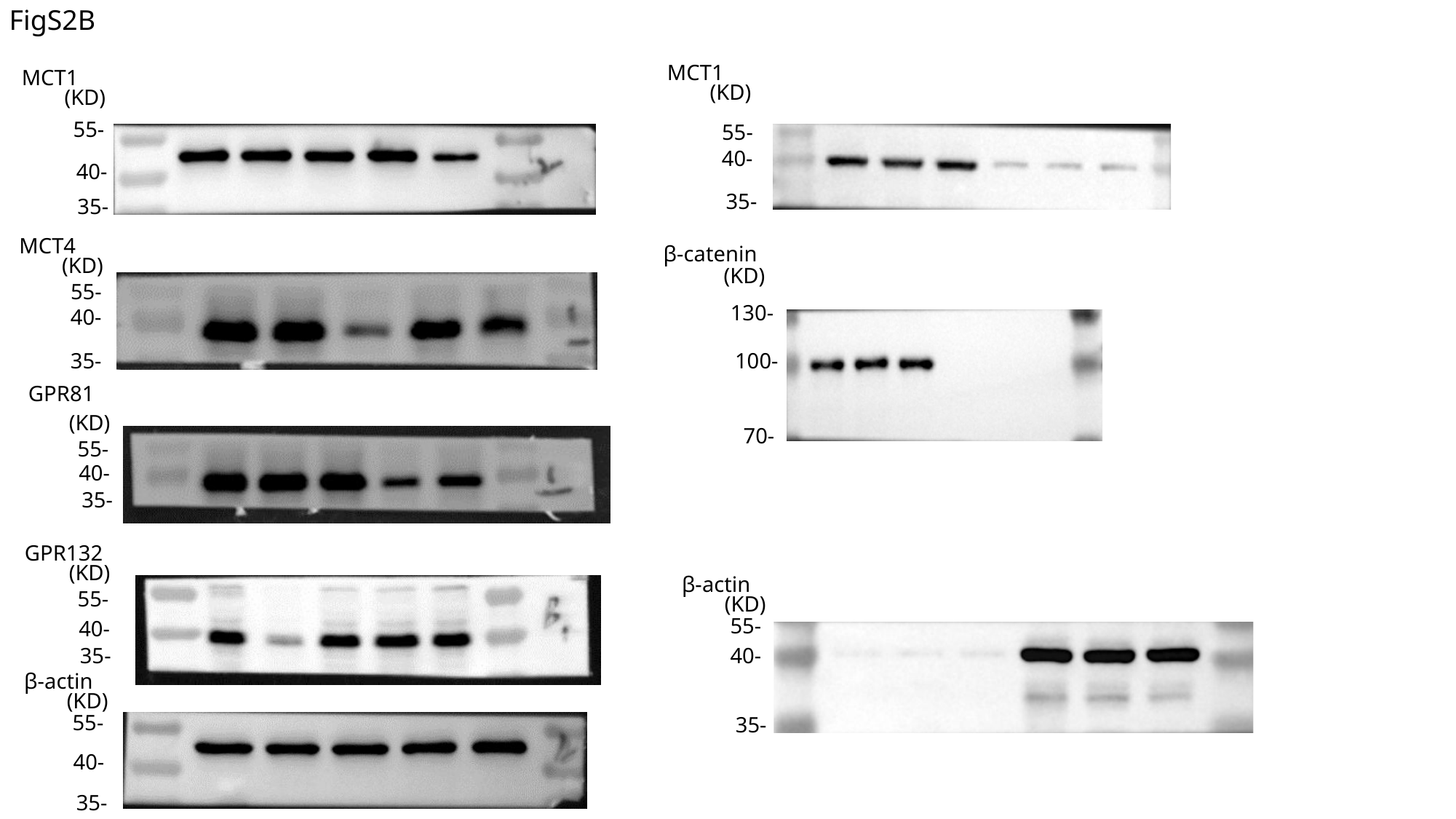

FigS2B
MCT1
MCT1
(KD)
(KD)
55-
55-
40-
40-
35-
35-
MCT4
β-catenin
(KD)
(KD)
55-
130-
40-
35-
100-
GPR81
(KD)
70-
55-
40-
35-
GPR132
(KD)
β-actin
55-
(KD)
55-
40-
40-
35-
β-actin
(KD)
55-
35-
40-
35-

## Slide 9
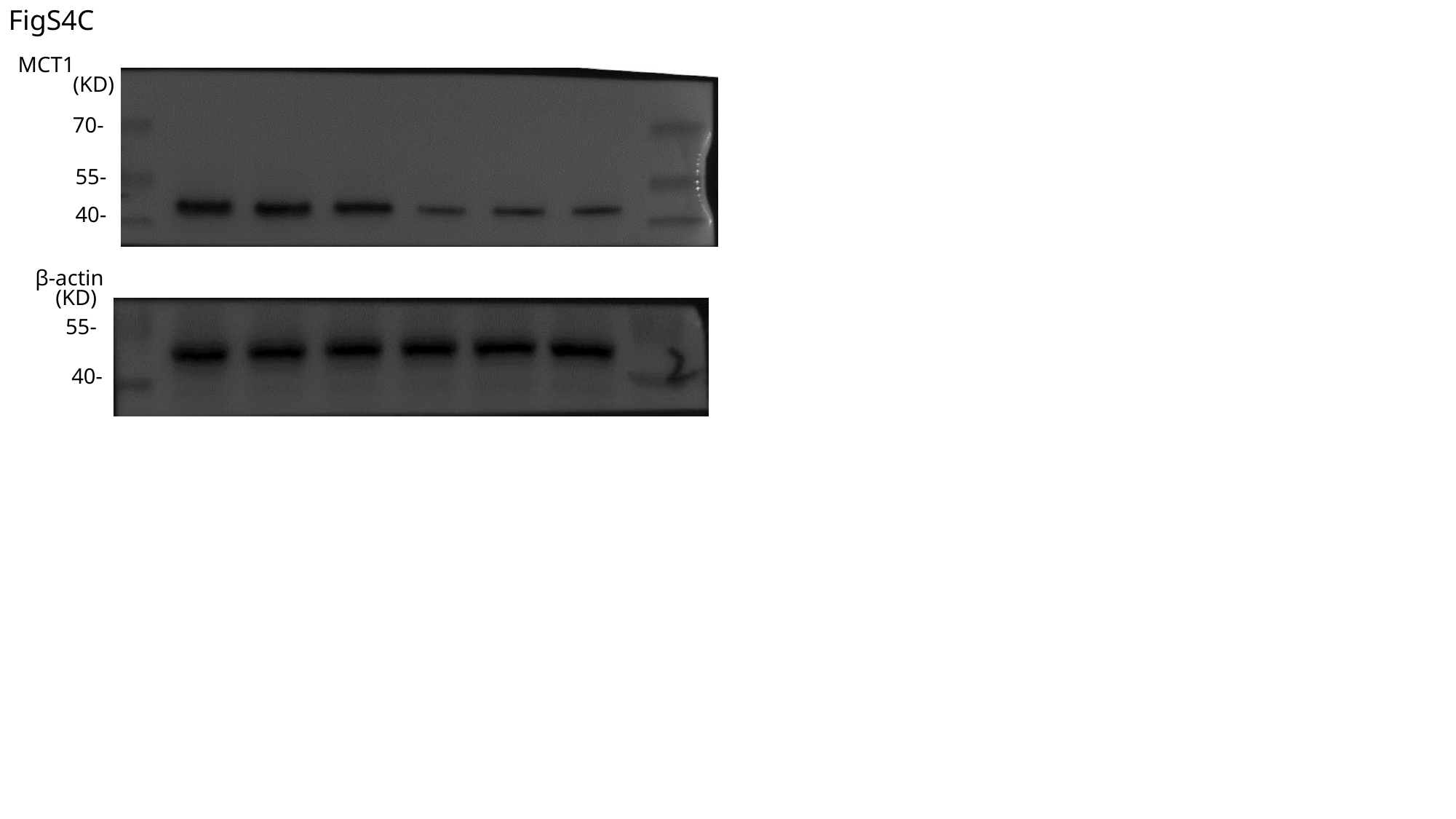

FigS4C
MCT1
(KD)
70-
55-
40-
β-actin
(KD)
55-
40-

## Slide 10
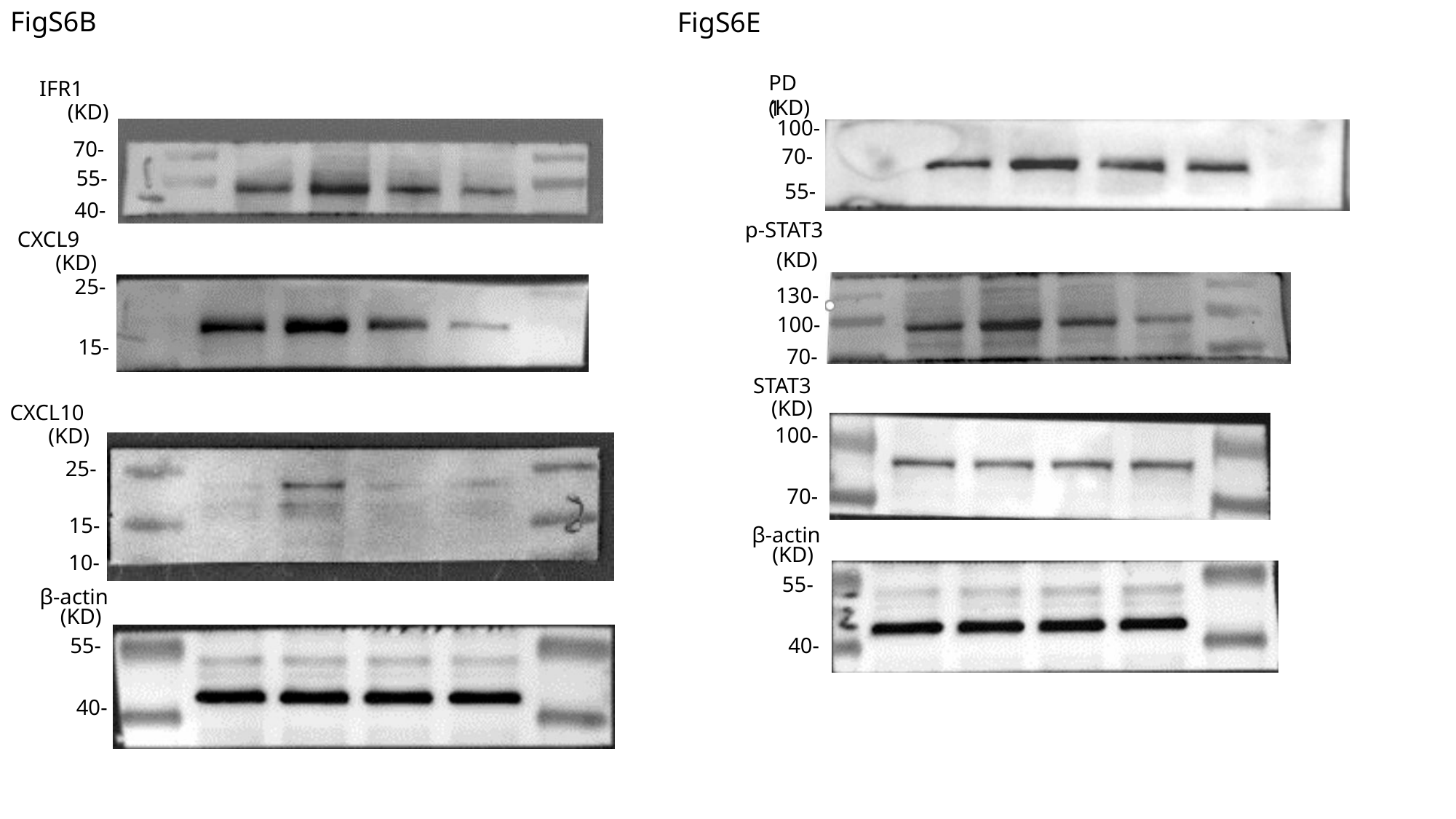

FigS6E
FigS6B
PD1
IFR1
(KD)
(KD)
100-
70-
70-
55-
55-
40-
p-STAT3
CXCL9
(KD)
(KD)
25-
130-
100-
15-
70-
STAT3
(KD)
CXCL10
100-
(KD)
25-
70-
15-
β-actin
(KD)
10-
55-
β-actin
(KD)
40-
55-
40-

## Slide 11
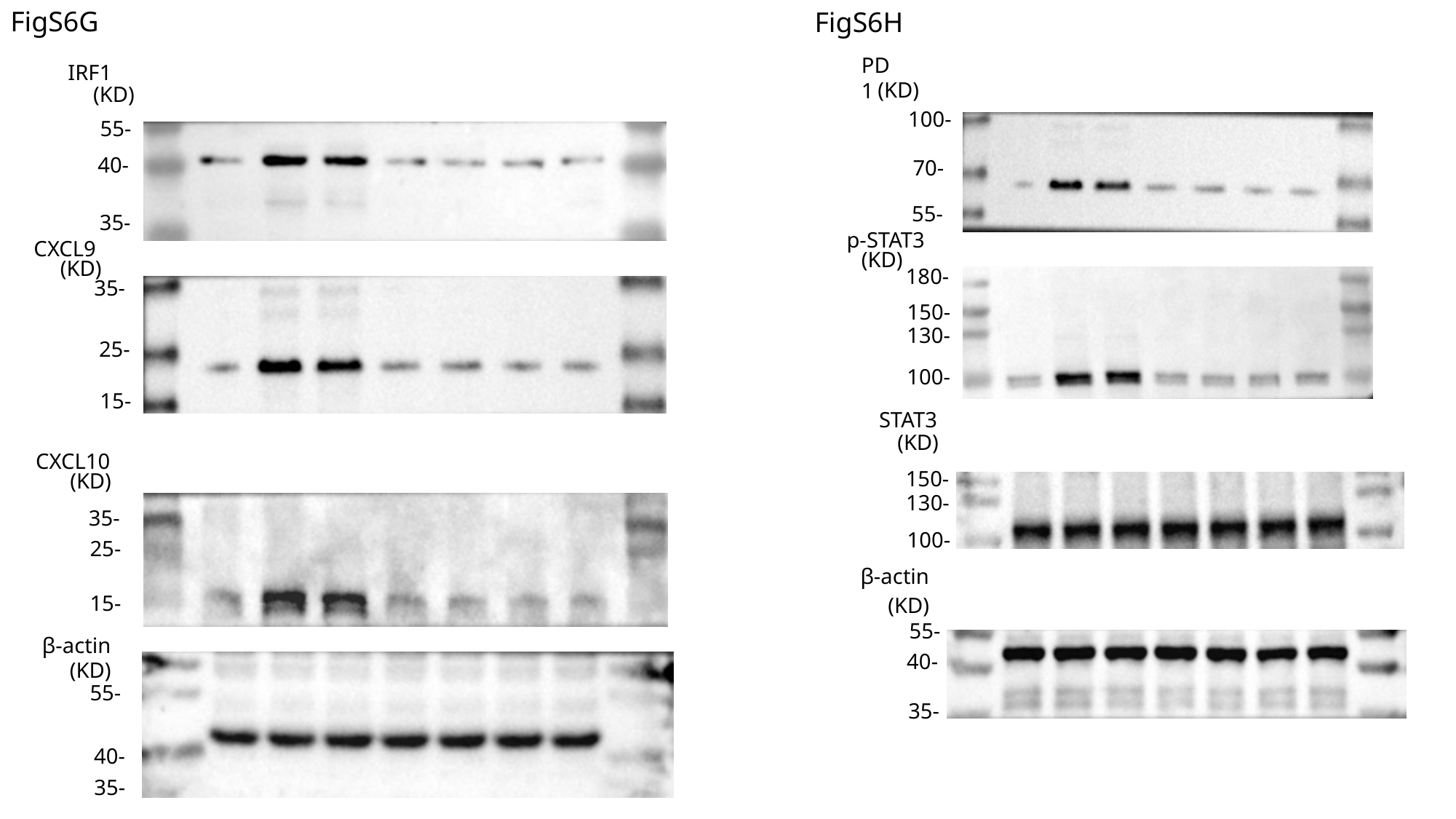

FigS6G
FigS6H
PD1
IRF1
(KD)
(KD)
100-
55-
40-
70-
55-
35-
p-STAT3
CXCL9
(KD)
(KD)
180-
35-
150-
130-
25-
100-
15-
STAT3
(KD)
CXCL10
150-
(KD)
130-
35-
100-
25-
β-actin
15-
(KD)
55-
β-actin
40-
(KD)
55-
35-
40-
35-

## Slide 12
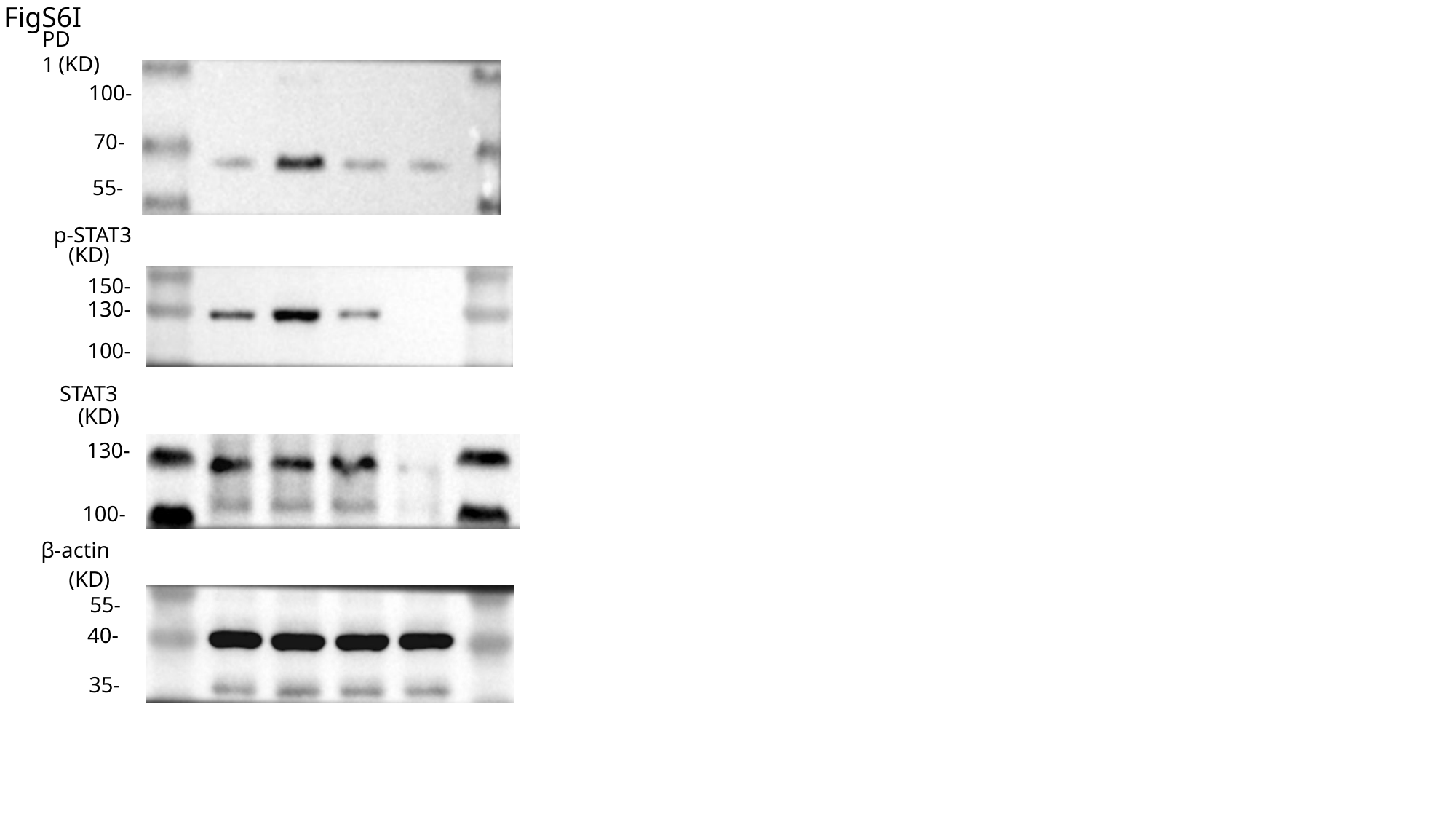

FigS6I
PD1
(KD)
100-
70-
55-
p-STAT3
(KD)
150-
130-
100-
STAT3
(KD)
130-
100-
β-actin
(KD)
55-
40-
35-

## Slide 13
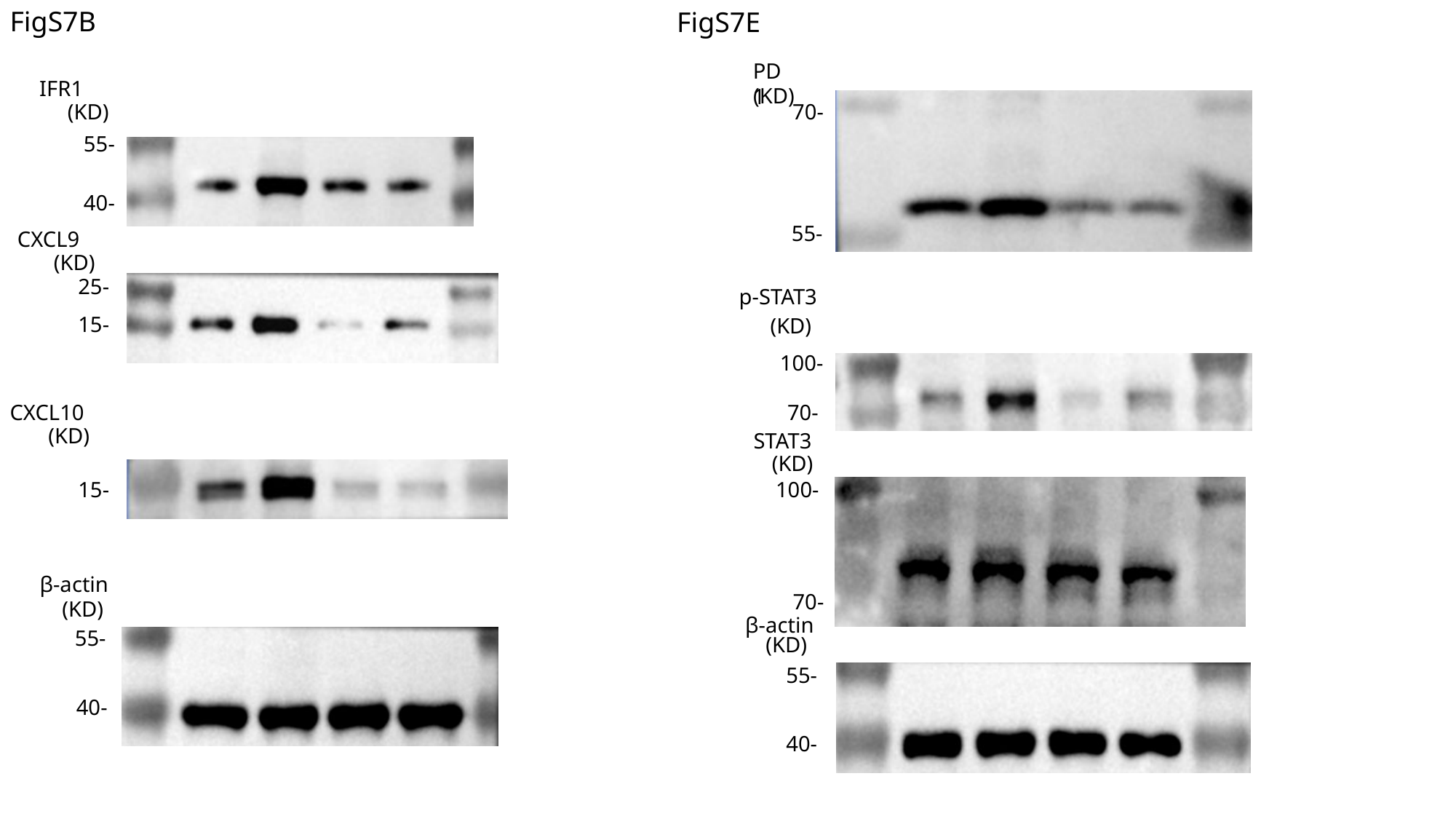

FigS7E
FigS7B
PD1
IFR1
(KD)
(KD)
70-
55-
40-
55-
CXCL9
(KD)
25-
p-STAT3
15-
(KD)
100-
CXCL10
70-
(KD)
STAT3
(KD)
15-
100-
β-actin
70-
(KD)
β-actin
55-
(KD)
55-
40-
40-

## Slide 14
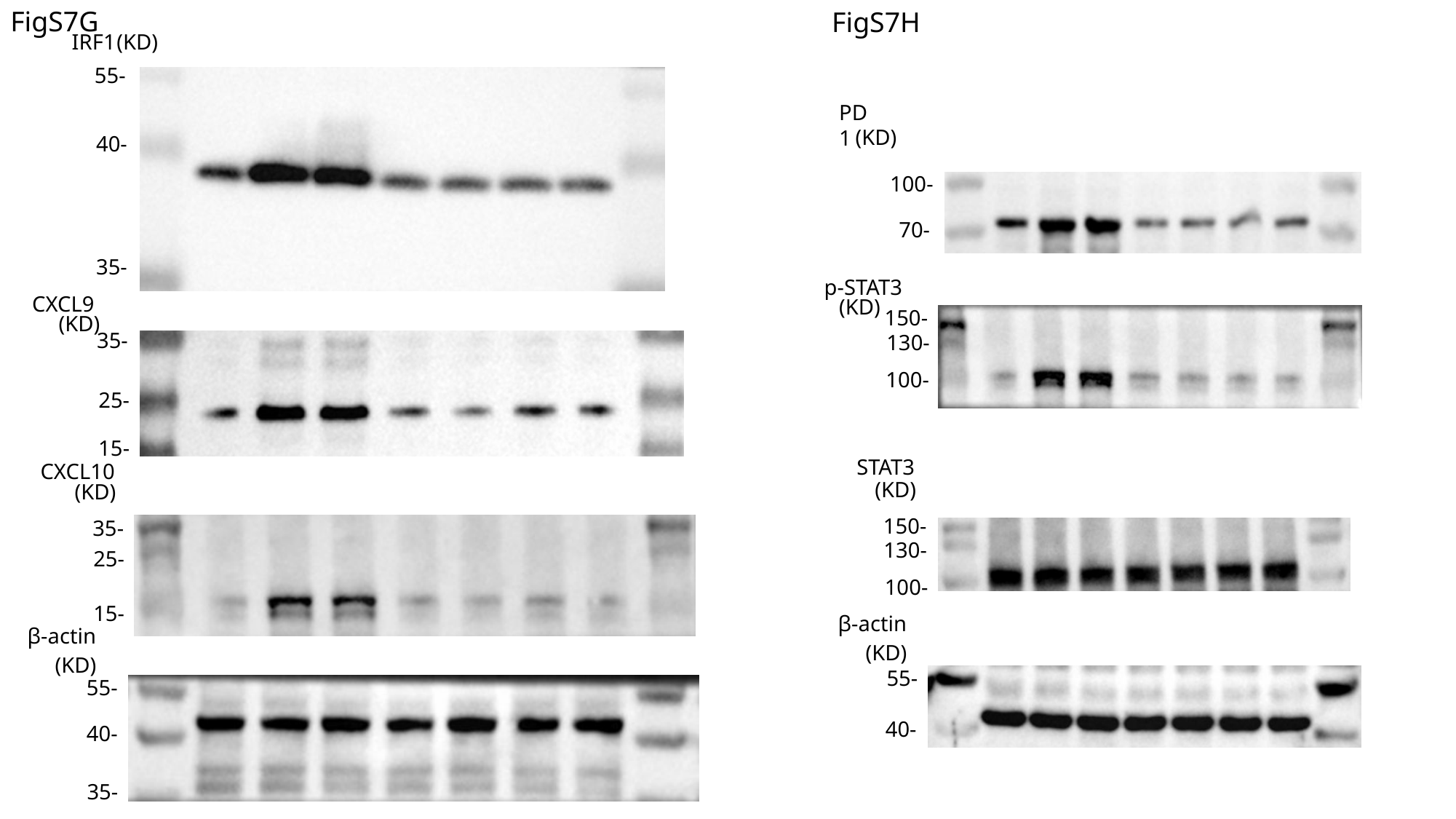

FigS7G
FigS7H
(KD)
IRF1
55-
PD1
(KD)
40-
100-
70-
35-
p-STAT3
CXCL9
(KD)
150-
(KD)
35-
130-
100-
25-
15-
STAT3
CXCL10
(KD)
(KD)
150-
35-
130-
25-
100-
15-
β-actin
β-actin
(KD)
(KD)
55-
55-
40-
40-
35-

## Slide 15
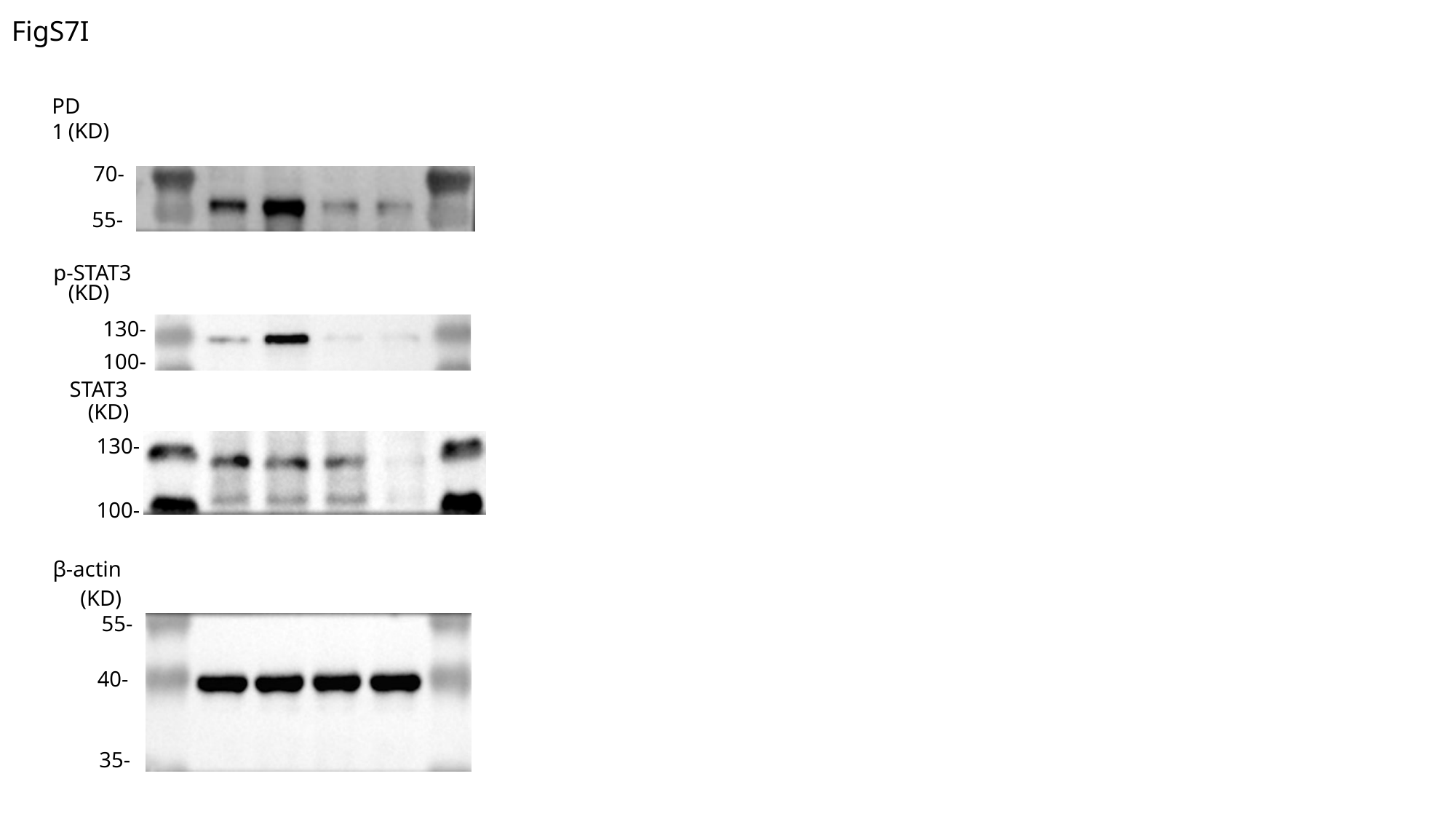

FigS7I
PD1
(KD)
70-
55-
p-STAT3
(KD)
130-
100-
STAT3
(KD)
130-
100-
β-actin
(KD)
55-
40-
35-
